# Reducing branched-chain amino acids improves cardiac stress response in mice by decreasing histone H3K23 propionylation

**DOI:** 10.1172/JCI169399

**Published:** 2023-11-15

**Authors:** Zhi Yang, Minzhen He, Julianne Austin, Danish Sayed, Maha Abdellatif

**Affiliations:** Department of Cellular Biology and Molecular Medicine, Rutgers University-New Jersey Medical School, Newark, New Jersey, USA.

**Keywords:** Cardiology, Metabolism, Cardiovascular disease, Epigenetics, Transcription

## Abstract

Identification of branched-chain amino acid (BCAA) oxidation enzymes in the nucleus led us to predict that they are a source of the propionyl-CoA that is utilized for histone propionylation and, thereby, regulate gene expression. To investigate the effects of BCAAs on the development of cardiac hypertrophy and failure, we applied pressure overload on the heart in mice maintained on a diet with standard levels of BCAAs (BCAA control) versus a BCAA-free diet. The former was associated with an increase in histone H3K23-propionyl (H3K23Pr) at the promoters of upregulated genes (e.g., cell signaling and extracellular matrix genes) and a decrease at the promoters of downregulated genes (e.g., electron transfer complex [ETC I–V] and metabolic genes). Intriguingly, the BCAA-free diet tempered the increases in promoter H3K23Pr, thus reducing collagen gene expression and fibrosis during cardiac hypertrophy. Conversely, the BCAA-free diet inhibited the reductions in promoter H3K23Pr and abolished the downregulation of ETC I–V subunits, enhanced mitochondrial respiration, and curbed the progression of cardiac hypertrophy. Thus, lowering the intake of BCAAs reduced pressure overload–induced changes in histone propionylation–dependent gene expression in the heart, which retarded the development of cardiomyopathy.

## Graphical abstract

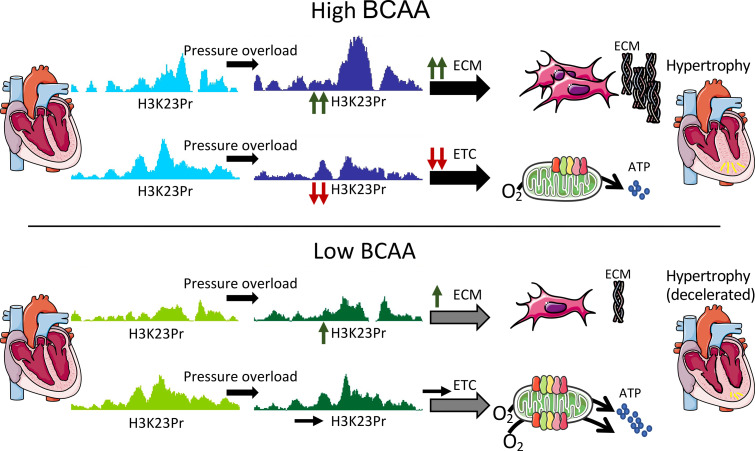

## Introduction

Transcriptional homeostasis is fundamental to our health and adaptability, as its disruption underlies the pathogenesis of a broad range of diseases. The codes that regulate transcriptional initiation, pausing, the elongation rate, silencing, or memory are written by a highly intricate set of histone posttranslational modifications that have yet to be fully deciphered. To put this in perspective, histone H3 has 13 Lys residues, with 11 known modifiers so far (1), potentially resulting in over 34 × 10^9^ permutations of modified H3. In general, studies have focused on histone acetylation and methylation as modifiers of a gene’s transcriptional activity since they were the first discovered and are highly abundant. During the past decade, however, more modifiers have been identified by mass spectrometry, including several short-chain fatty acid (SCFA) modifications (1), such as propionylation, butyrylation, and crotonylation, whose role in gene expression remain to be investigated.

We recently identified branched-chain amino acid (BCAA) oxidation enzymes in both human and rodent nuclei (2), which suggested that they may be a source of localized propionyl-CoA that can be utilized for histone modification, particularly since CoA metabolic intermediates are not readily exported from the mitochondria. This was an unbiased discovery that we uncovered by ChIP using anti-H2A.Z, followed by mass spectrometry of the immunocomplex, which revealed the association of the TCA cycle, fatty acids, and BCAA oxidation enzymes with chromatin (2). These findings are not unprecedented, as others have also reported that pyruvate dehydrogenase and oxoglutarate dehydrogenase complexes are present in the nucleus, where they produce acetyl-CoA and succinyl-CoA, respectively, for the corresponding histone modifications (3, 4). SCFAs, including propionate, are also produced in the intestine from the digestion of dietary fibers by *Bacteroidetes* and *Firmicutes* (5). Propionate absorbed from the intestine can potentially be converted to propionyl-CoA (the form necessary for protein modification) via acyl-CoA short-chain synthase 1/2 (ACSS1/2), as extrapolated from a study showing that it catalyzes the conversion of butyrate to butyryl-CoA (6). Other sources of propionyl-CoA include oxidation of methionine and threonine, and odd-chain fatty acids (7). Alternatively, we predicted that propionyl-CoA is produced as an intermediate of BCAA (isoleucine and valine) oxidation in the nucleus and is required for histone propionylation.

The dogma is that a diet low in protein improves metabolic health and that its benefits can be reproduced by restricting dietary BCAAs (8–12). In support of these findings, high levels of circulating BCAAs are linked to glucose intolerance and type 2 diabetes (13). Also, high dietary BCAA results in hyperphagia, obesity, and a shortened lifespan in mice (14), as well as obesity in humans (15), and is associated with the development of specific cancers (16) and cardiometabolic diseases (17). Accordingly, limiting dietary BCAAs protects against many of these pathologies (13). However, although low dietary BCAAs maybe beneficial, defects in their oxidation may be detrimental (18–20). This is not absolute, though, since deletion of branched chain amino acid aminotransferase, mitochondrial (BCATm) increases insulin sensitivity in the heart (21). On the other hand, extracardiac catabolism of BCAA has a cardioprotective effect via the mediation of hypotension (22).

Recently, Trefely et al. reported that propionyl-CoA is substantially enriched in the nucleus compared with the cytosol (23). Using stable isotope labeling of essential nutrients, they were able to trace the source of propionyl-CoA to isoleucine, which contributed to the propionylation of H2AK5, H4K16, and H3K23, but not H2K14 (23). However, the effect of dietary BCAAs on the dynamics of propionylated histones or their influence on transcriptional regulation in health or disease remains largely unexplored. There is evidence, however, that high levels of propionyl-CoA or propionate may be pathogenic, since genetic defects in propionyl-CoA carboxylase (PCC) results in the accumulation of propionyl-CoA and propionic acidemia, which is accompanied by a high incidence of cardiomyopathy (24). Also, high propionate produced by gut *Bacteroidetes* has been linked to Alzheimer’s disease (5). Thus, high levels of BCAAs, propionyl-CoA, or propionate, all of which can potentially increase histone propionylation, have adverse effects on our health.

In this study, we show that a diet containing standard levels of BCAAs (equivalent to those found in conventional rodent chow) was associated with work overload–induced increases in histone H3K23–propionyl (H3K23Pr) within the promoters of upregulated genes (e.g., ECM and Ki67 genes in myofibroblasts), or decreases within the promoters of downregulated genes (e.g., metabolic and ETC genes). These changes were reduced or even completely abolished by feeding the mice a BCAA-free diet. The suppression of pressure overload–induced upregulation of H3K23Pr and gene expression by the BCAA-free diet was counteracted by supplementing it with propionate, suggesting the involvement of histone propionylation in mediating the effects of BCAA on gene expression.

## Results

### BCAA is required for histone propionylation.

**Figure 1:**
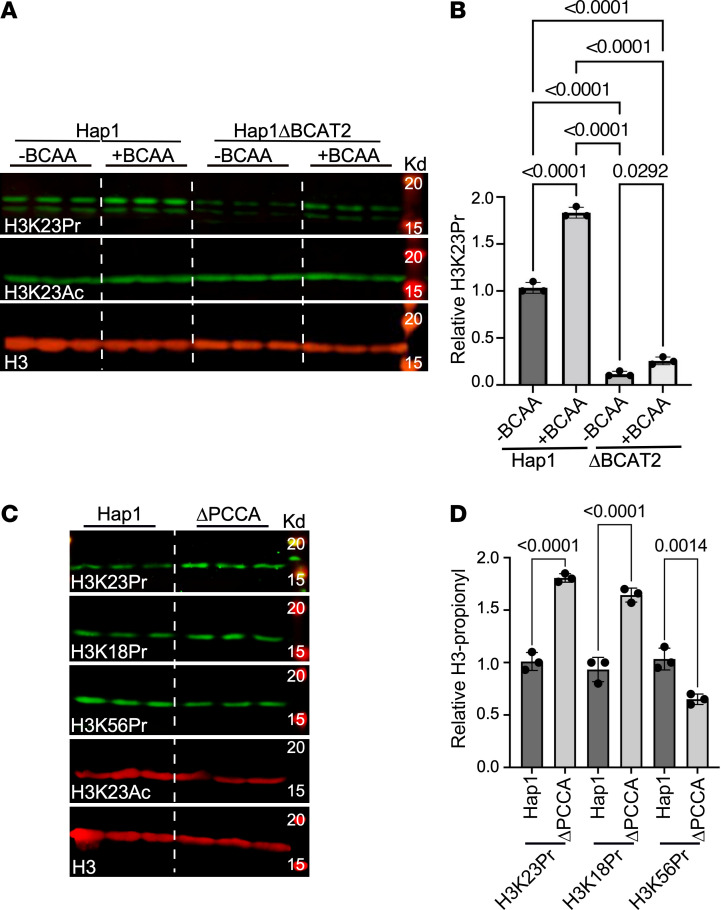
BCAT2 and PCCA regulate H3 propionylation. (**A**) Hap1 cells and those with a deletion in the *BCAT2* gene (Hap1Δ*BCAT2*), were cultured in either full DMEM medium (+BCAA) or medium lacking BCAA (–BCAA, 0 mM), with no FBS. After 16 hours, histones were extracted and analyzed by WB with the antibodies listed on the left of each panel. (**B**) The H3K23Pr signals observed on the Western blot were quantitated, normalized to H3, and plotted as relative values after adjusting the signal of –BCAA samples to 1 (*n* = 3). Data were analyzed by 1-way ANOVA, and *P* values of 0.05 or less are indicated on the graph. (**C**) Similarly, Hap1 cells and those with a deletion of the *PCCA* gene (Hap1Δ*PCCA*), were cultured in medium with reduced BCAAs (0.1× BCAA, i.e., one-tenth of its concentration in the standard DMEM), with no FBS. After 16 hours, histones were extracted and analyzed by WB with the antibodies listed on each panel. (**D**) The signals observed on the Western blot were quantitated, normalized to H3, and plotted as relative values after adjusting the signal of one of the Hap1 samples to 1 (*n* = 3). The data were analyzed by 2-tailed, equal-variance Student’s *t* test for Hap1 versus Δ*PCCA* values, and *P* values of 0.05 or less are shown on the graph.

We have previously reported the presence of metabolic oxidation enzymes, including those for BCAAs, in the nuclei of both rodent and human cells (2) (Supplemental Figure 1; supplemental material available online with this article; https://doi.org/10.1172/JCI169399DS1). This led us to speculate that oxidation of isoleucine and valine produces the intermediate propionyl-CoA, which could be utilized for histone modification. Histone H3 lysine 23–propionyl (H3K23Pr) was the modification of choice for monitoring the effect of BCAAs on histone propionylation, since our initial experiments suggested its correlation with gene expression. Using human Hap1 cells, we show that culturing cells in DMEM with the standard amount of BCAA (+BCAA) versus a BCAA-free (–BCAA) DMEM medium for 16 hours, without serum, had significantly higher H3K23Pr levels relative to insignificant changes in acetylation (Figure 1, A and B). Notably, however, the BCAA-free medium did not completely abolish the H3-propionyl signal, plausibly due to propionyl-CoA produced from threonine or methionine or from protein turnover. The deletion of BCAT2 (Supplemental Figure 2, A–C) — the enzyme that catalyzes the first step in BCAA oxidation — further reduced H3K23 propionylation (Figure 1, A and B), supporting the latter possibility. These results support the hypothesis that BCAA oxidation was directly responsible for histone propionylation in human cells. To confirm this, we examined whether deletion of propionyl-CoA carboxylase (PCCA) — the enzyme that catalyzes the carboxylation of propionyl-CoA to methylmalonyl-CoA —would result in an increase histone propionylation. Consistently, we found that knockout of PCCA (Supplemental Figure 2, D and E) increased H3K23Pr and H3K18Pr, but not H3K56Pr (Figure 1, C and D), although this confirmed the role of BCAA oxidation in histone propionylation, as it also revealed its spatial specificity.

### A BCAA-free diet decelerates work overload–induced cardiac hypertrophy.

**Figure 2:**
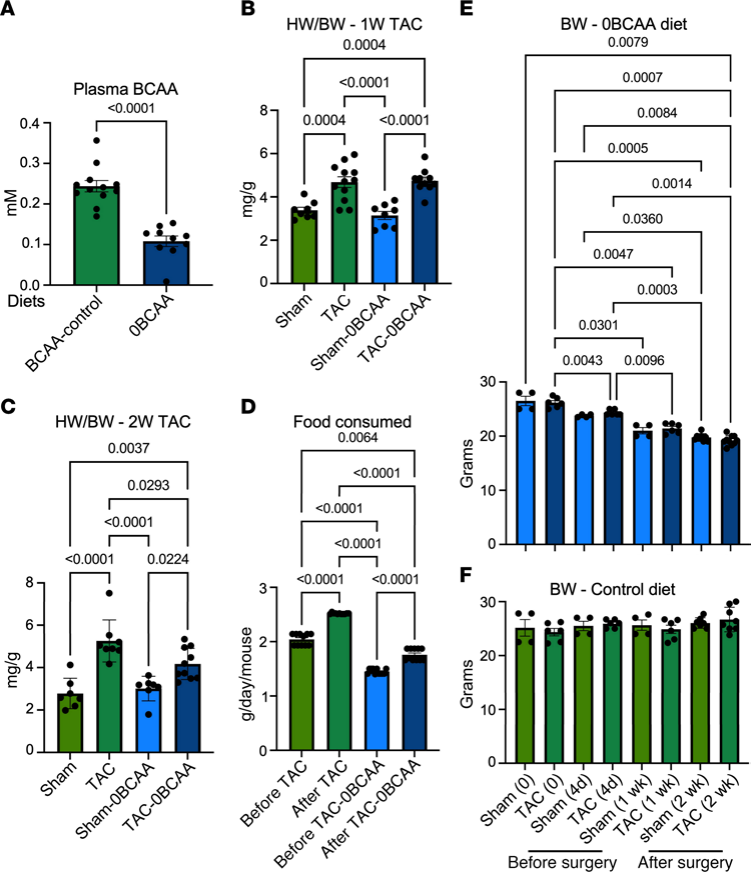
A BCAA-free diet slows the progression of cardiac hypertrophy, inhibits promoter H3K23Pr content, and diminishes changes in mRNA expression. (**A**) Twelve-week-old mice were fed either a BCAA control diet or a BCAA-free (0BCAA) diet for 11 days, after which serum was collected from the left ventricle and assayed for BCAA levels. The results were graphed and analyzed by Student’s *t* test (*n* = 10–12); *P* values are shown above the brackets encompassing the bars. (**B**–**F**) Twelve-week-old mice were fed either a BCAA control diet or a BCAA-free (0BCAA) diet for 4 days before being subjected to sham or TAC surgery (*n* = 8–12, each group). The mice were then maintained on the same diets for either 1 week (1W) or 2 weeks (2W). (**B** and **C**) The mice were assessed by echocardiography (**B)** 1 week (*n* = 7–12) or (**C**) 2 weeks (*n* = 10–12) after TAC, and the corrected HW/BW (mg/g) was calculated and graphed. (**D**) The food consumed (g/day/mouse) was calculated and graphed (*n* = 10, 5 mice/cage). (**E** and **F**) BW (g) was measured and graphed. The results for the data in **B**–**F** were analyzed by 1-way ANOVA, and the *P* values of those that are 0.05 or less are given above the brackets encompassing the bars.

To test the effect of dietary BCAAs on cardiac hypertrophy, we fed mice a control diet (with all essential aa, including BCAA), or a BCAA-free (0BCAA) diet (with all essential aa except for BCAA and 0BCAA). After 4 days of feeding, workload was imposed on the heart by transverse aortic constriction (TAC) or sham surgery. One week after surgery, during which the mice were maintained on the same diets, serum BCAA concentrations were measured. The levels fell by an average of 59% in mice on the BCAA-free diet (Figure 2A). While food is the major source of BCAAs, these essential aa are also supplied by the gut microbiota (25). We confirmed the presence of *Verrucomicrobia*, *Bacteroidetes*, and *Firmicutes* in these mice, using shotgun sequencing of the intestine. The results showed that these were more prevalent with the BCAA-free diet (Supplemental Figure 3), plausibly because of a feedback mechanism to sustain the body’s requirements for BCAAs.

Mice were also assessed by echocardiography, the results of which show that both the control diet and BCAA-free diets exhibited equivalent increases (an average of 25%) in heart weight/body weight (HW/BW) within 1 week of TAC (1W TAC, Figure 2B). Two weeks after TAC, however, the HW/BW continued to increase (an average of 35%) in mice on the control diet, while plateauing in mice on the BCAA-free diet (Figure 2C). Note, the BCAA-free diet resulted in reduced food intake (Figure 2D) and weight loss (Figure 2E) versus the BCAA control diet (Figure 2F), consistent with previous reports (26), but it did not affect the mice’s apparent general health or activity. These results suggest that lowering dietary BCAAs decelerates the progression of pressure overload–induced hypertrophy.

### A BCAA-free diet blunts work overload–induced increases in promoter H3K23Pr and moderates changes in gene expression.

**Figure 3:**
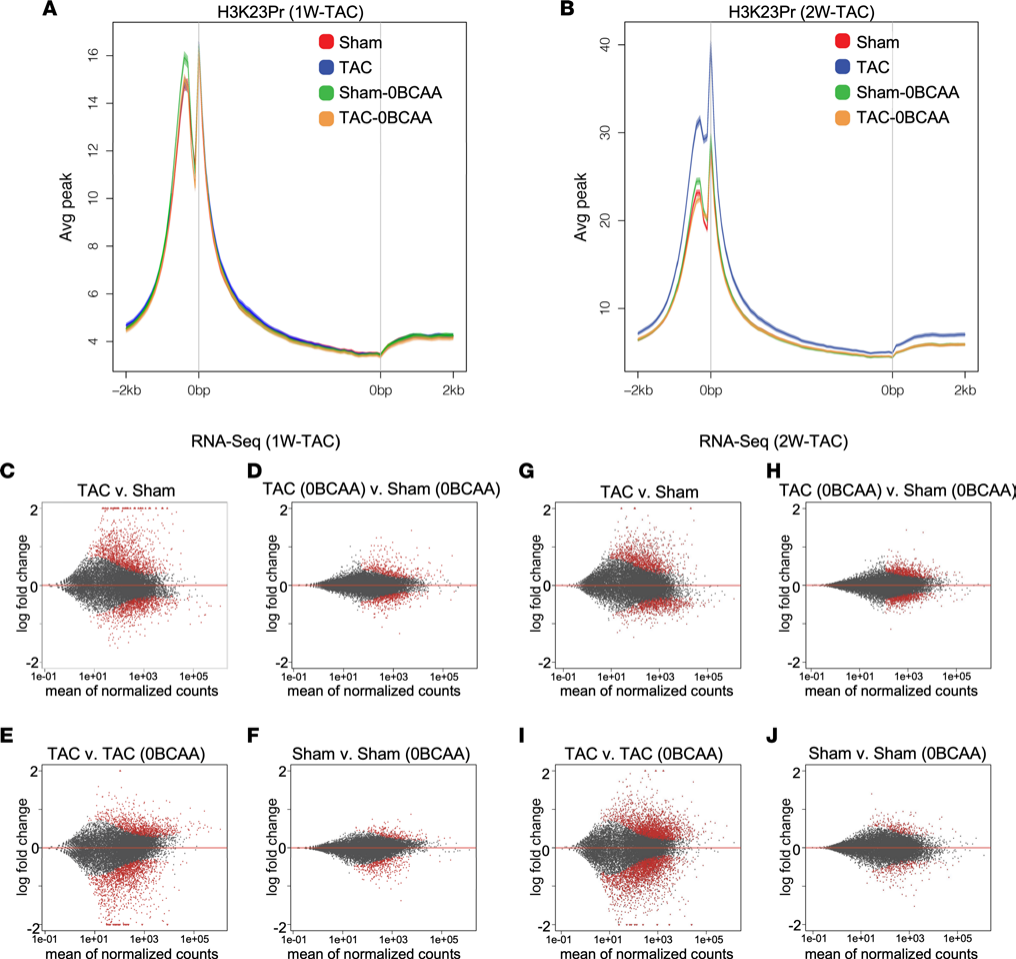
A BCAA-free diet inhibits promoter H3K23Pr content and reduces pressure overload–induced mRNA expression. (**A** and **B**) Chromatin was extracted from the heart and assayed by H3K23Pr ChIP-Seq, both (**A**) 1 week and (**B**) 2 weeks after TAC (date from 3 hearts each were pooled). The average (Avg) sequence tags from the ChIP-Seq results were graphed across gene promoters and gene bodies (–2 kb from TSS to +2 kb from gene end). (**C**–**J**) RNA was extracted from the heart and analyzed by RNA-Seq (*n* = 3 each). The results are displayed in MA plots of LFC (*y* axis) versus the mean of normalized counts (*x* axis) for comparisons between the different samples. (**C** and **G**) TAC versus sham (BCAA control diet for both). (**D** and **H**) TAC versus sham (0BCAA diet for both). (**E** and **I**) TAC (control) versus TAC (0BCAA). (**F** and **J**) Sham (control) versus sham (0BCAA). Each dot represents the values for a single gene; red dots indicate a *P* value of 0.1 or less, and gray dots indicate a *P* value > 0.1.

To determine the effect of dietary BCAA on histone propionylation and gene expression, we treated the mice as described in the previous section (Figure 2). After 1 week or 2 weeks of imposed work overload (1W TAC or 2W TAC) on the heart, chromatin or total RNA was extracted from the left ventricle. Chromatin was immunoprecipitated using anti-H3K23Pr, after confirming the specificity of the antibody (Supplemental Figure 4). The results of ChIP-Seq reveal enrichment of this histone mark at gene promoters (Figure 3, A and B, accessions GSE227227 and GSE227228, superseries GSE229131). One week after TAC, there were no discernible differences in the average of total promoter H3K23Pr peaks across the genome (more than 12,000 expressed genes detected in the mouse heart), in the sham-operated versus TAC hearts, or in the hearts of mice on the BCAA control diet versus those on a BCAA-free diet. The absence of a difference between those conditions may be due the residual (~40%) circulating BCAA observed with the BCAA-free diet (Figure 2A), since BCAAs are also produced by the gut microbiome (Supplemental Figure 3). This also explains the differences between the in vivo data versus the cultured cells that were deprived of BCAA (Figure 1A). Furthermore, changes in the promoter H3K23Pr of genes that were induced within 1 week of TAC (see below) — 8% upregulated and 6% downregulated — were not reflected in the promoter’s average peak for all genes (Figure 3A). In contrast to the 1-week TAC, after 2 weeks of continuous work overload, a distinct increase in average promoter H3K23Pr peak became evident in mice on the control diet but not in those on the BCAA-free diet (Figure 3B; accession GSE227228, superseries GSE229131). The data suggest that dietary BCAA is involved in work overload–induced promoter H3K23 propionylation in the heart.

We performed RNA-Seq analysis of cardiac gene expression in similarly treated mice. The log ratio/mean average (MA) plots (log_2_ fold change [LFC] vs. the mean of normalized counts) revealed that 1 week after pressure overload (TAC), the number of genes and the extent of change of their mRNA levels (log_2_ TAC/sham) were much greater in the hearts of mice on a BCAA control diet (Figure 3C) versus those on a BCAA-free (0BCAA) diet (Figure 3D; accession GSE227110, superseries GSE229131). This was also reflected in the MA plot comparing TAC (control) versus TAC (0BCAA) (Figure 3E). In contrast, dietary BCAA had a minimal impact on gene expression in the unstressed (sham) hearts (Figure 3F). The effect of dietary BCAA on gene expression was reproduced in the 2-week TAC hearts (Figure 3; G–J, accession GSE229128, superseries GSE229131). Thus, dietary BCAAs accounted for the increase in promoter H3K23Pr within 2 weeks of pressure overload–induced cardiac hypertrophy and were associated with robust changes in gene expression compared with a BCAA-free diet.

### Changes in promoter H3K23Pr correlate with changes in gene expression.

**Figure 4:**
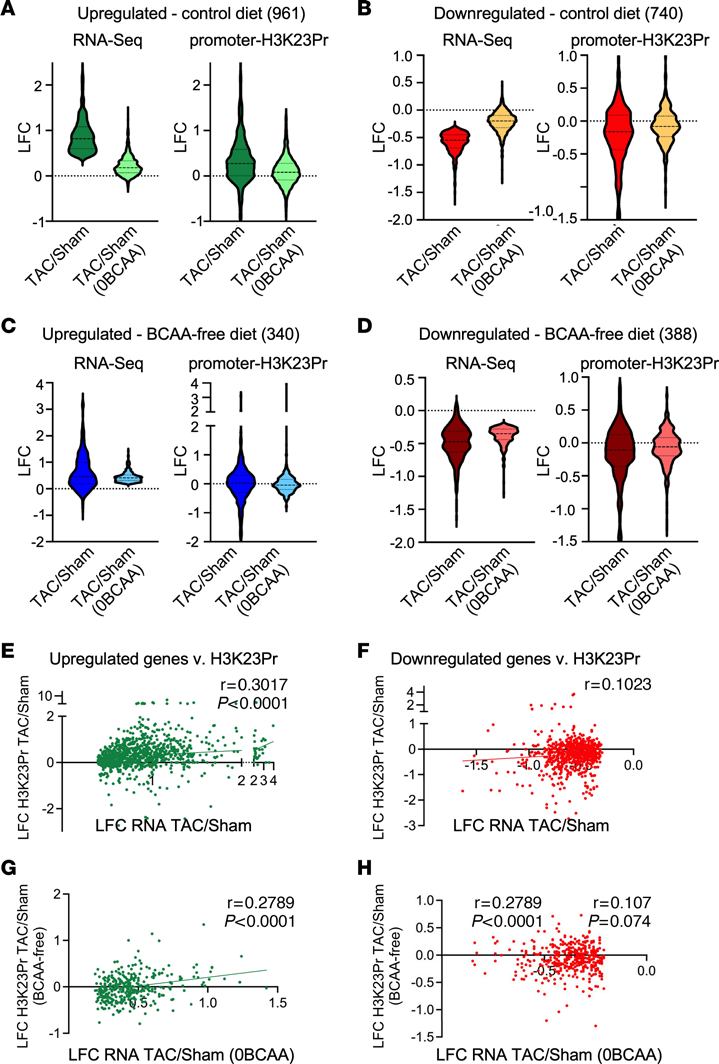
Pressure overload–induced mRNA expression correlates with changes in promoter H3K23Pr and is depressed by a BCAA-free diet. (**A**–**H**) H3K23Pr ChIP-Seq (–1,000 to +1,000) and RNA-Seq data from the 1-week TAC experiment described in Figure 3 were aligned by gene name and then sorted according to the genes’ mRNA LFCs for TAC/sham that were significantly upregulated (padj ≤ 0.05) in hearts of mice on (**A**) a control diet or (**C**) a BCAA-free (0BCAA) diet, or significantly downregulated (padj ≤ 0.05) in the hearts of mice on (**B**) a control diet or (**D**) a BCAA-free (0BCAA) diet. The LFCs of mRNA expression (RNA-Seq, left) and promoter H3K23Pr (right) changes in the TAC or sham-operated hearts from these mice are presented as violin plots. (**E**–**H**) Spearman’s correlation analyses of LFCs of mRNA expression versus LFCs of promoter H3K23Pr in TAC or sham-operated hearts showing (**E** and **G**) those for the significantly upregulated genes and (**F** and **H**) for the significantly downregulated genes.

The RNA-Seq reads were aligned with promoter H3K23Pr ChIP-Seq tags (–1,000 bp to +1,000 bp) for all genes, before sorting them by significant (adjusted *P* [padj] ≤ 0.05) changes in mRNA expression. We identified 961 mRNAs that were significantly (padj ≤ 0.05) upregulated (Figure 4A) and 740 that were significantly (padj ≤ 0.05) downregulated (Figure 4B) in the mouse heart after 1 week of imposed pressure overload. Those results are plotted as LFC of TAC/sham, alongside the LFC for the corresponding TAC/sham of their promoter H3K23Pr (Figure 4, A and B). Of those, 184 mRNAs were significantly (padj ≤ 0.05) upregulated and 231 mRNAs were significantly downregulated in the hearts of mice on a BCAA-free diet (padj ≤ 0.05), albeit to a significantly lesser extent. On the other hand, a total of 340 mRNAs were significantly (padj <0.05) upregulated (Figure 4C) and 388 were significantly (padj ≤ 0.05) downregulated (Figure 4D) in mice maintained on a BCAA-free diet. Of those, 188 mRNAs were significantly (padj ≤ 0.05) upregulated and 226 mRNAs were significantly downregulated in the hearts of mice on a BCAA control diet.

Correlation analysis was performed between the LFC of TAC/sham for mRNA of the upregulated genes with the BCAA control (*x* axis) versus the LFC of TAC/sham for the corresponding promoter H3K23Pr (*y* axis) (Figure 4E), and similarly for the BCAA-free diet (Figure 4G) and downregulated mRNA expression (Figure 4, F and H). The results showed that changes in mRNA expression positively correlated with changes in promoter H3K23Pr in the hearts of mice on a BCAA control diet (Figure 4, E and F). We observed a similar relationship with the upregulated genes in mice on a BCAA-free diet (Figure 4G), but not with the downregulated genes (Figure 4H). Persistent pressure overload (for up to 2 weeks after TAC) in mice on the BCAA control diet was associated with further increases in H3K23Pr across promoters of inducible genes (Supplemental Figure 5, A–H), with a resultant loss of correlation with gene expression with the BCAA control diet (Supplemental Figure 5, E and F), but not with those upregulated with the BCAA-free diet (Supplemental Figure 5G). This may be attributed to the downregulation of PCCA (Supplemental Figure 10A), which converts propionyl-CoA into methylmalonyl-CoA, resulting in accumulation of propionyl-CoA. Thus, dietary BCAA modulated stress-induced promoter H3K23Pr and gene expression.

### Dietary BCAAs mediate stress-induced increases in promoter H3K23 propionylation and can be substituted by propionate.

**Figure 5:**
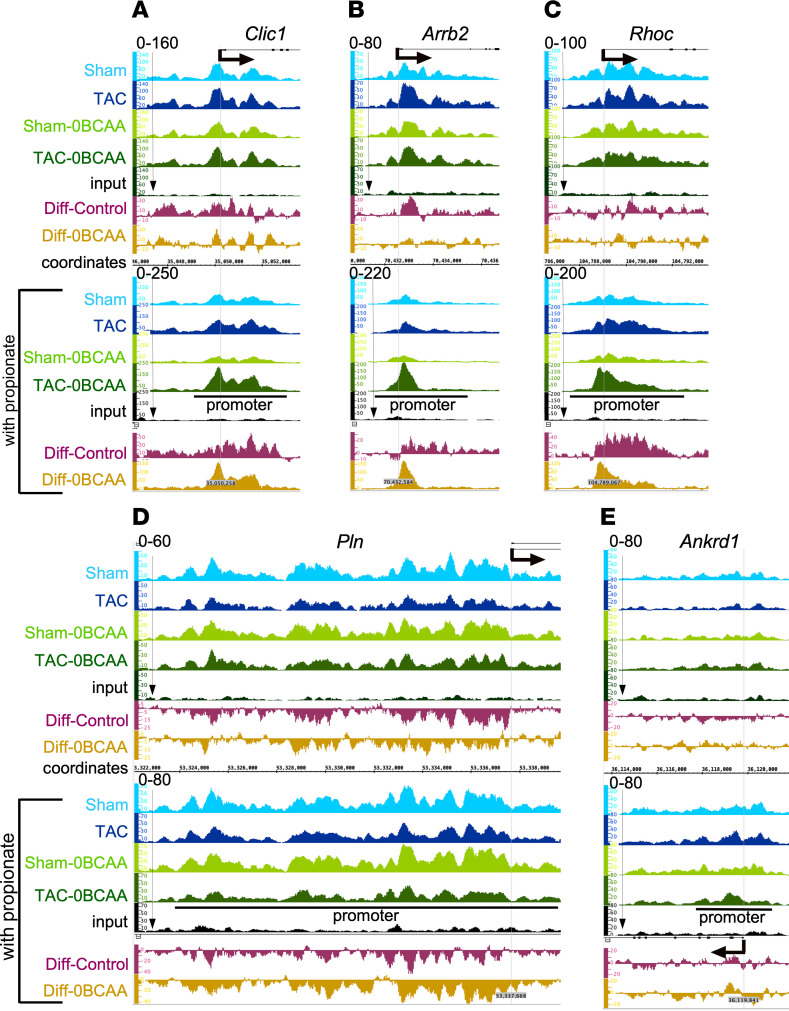
Dietary BCAAs and propionate regulate pressure overload–induced promoter H3K23Pr during cardiac hypertrophy. (**A**–**E**) IGB images showing H3K23Pr ChIP sequence tags from the sham-operated and TAC hearts of mice on the different diets without or with propionate supplementation, aligned across *Clic1*, *Arrb2*, *RhoC*, *Pln*, and *Ankrd1* gene coordinates. The labels on the left of each track indicate the surgical and diet conditions applied in the mice: sham and TAC surgeries with the BCAA control diet (blue tracks), with the BCAA-free diets (green tracks); the differences (Diff) in the H3K23Pr sequence tags of TAC minus sham for the control diet (Diff-Control, brown tracks), and BCAA-free diet (Diff-0BCAA, gold tracks) are shown in separate tracks for the diets with or without propionate, as indicated.

**Figure 6:**
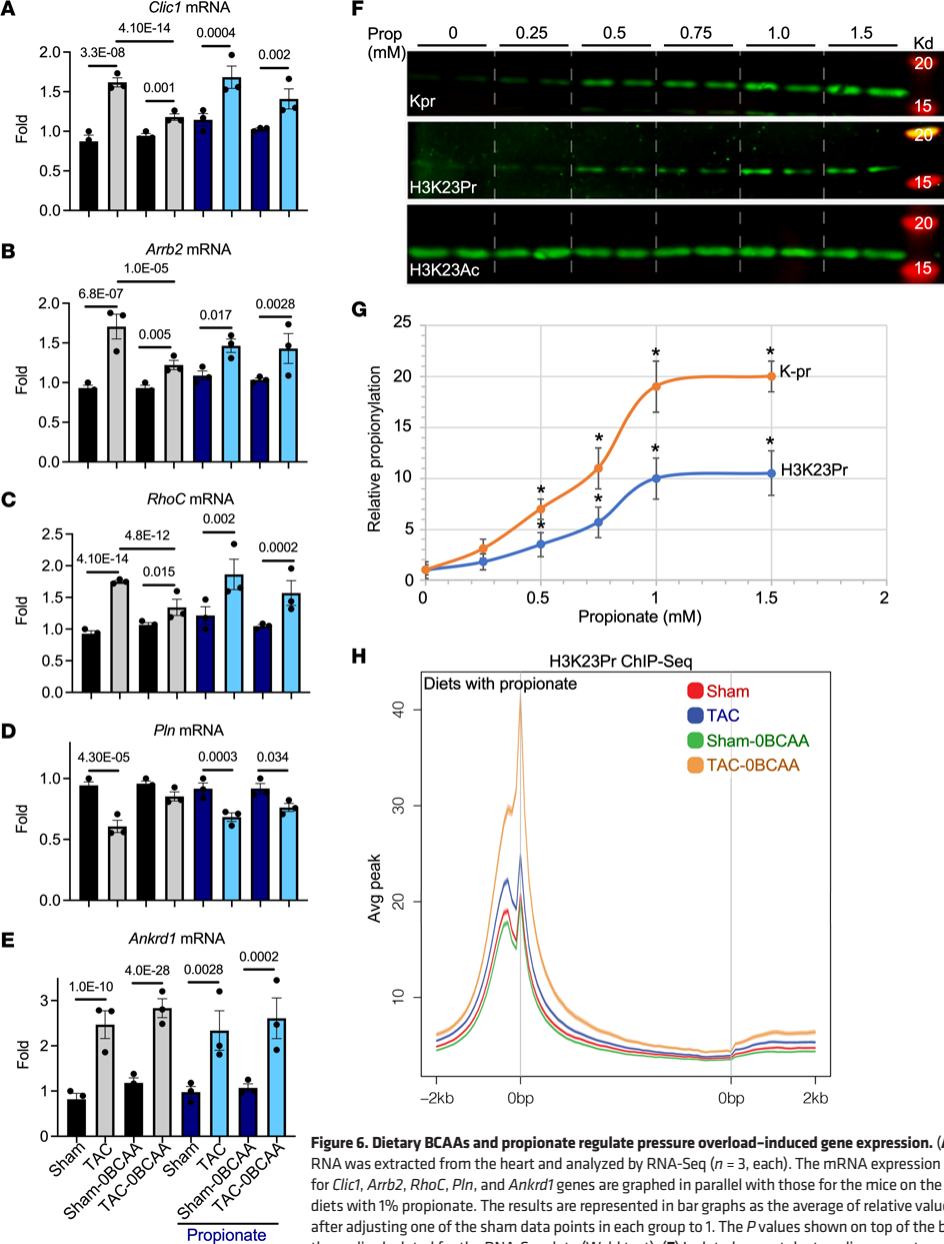
Dietary BCAAs and propionate regulate pressure overload–induced gene expression. (**A**–**E**) RNA was extracted from the heart and analyzed by RNA-Seq (*n* = 3, each). The mRNA expression data for *Clic1*, *Arrb2*, *RhoC*, *Pln*, and *Ankrd1* genes are graphed in parallel with those for the mice on the same diets with 1% propionate. The results are represented in bar graphs as the average of relative values after adjusting one of the sham data points in each group to 1. The *P* values shown on top of the bars are the padj calculated for the RNA-Seq data (Wald test). (**F**) Isolated neonatal rat cardiac myocytes were cultured overnight in reduced BCAA (0.1×) medium (DMEM) before they were treated with increasing doses of propionate. Histones were extracted and analyzed by WB for the protein listed on the left of each panel. (**G**) Lys-propionyl (Kpr) and H3K23Pr signals were quantified and graphed (*n* = 3). *P ≤ 0.05 versus control (no propionate) using 2-tailed *t* test. (**H**) Mice were treated as described in Figure 2, with the exception that both the BCAA control and BCAA-free diets were supplemented with 1% propionate. Chromatin was extracted from the heart and analyzed by H3K23Pr ChIP-Seq 1 week after TAC (date were pooled from 3 hearts each). The average sequence tags from the ChIP-Seq results were graphed across gene bodies (–2 kb from the gene start (distal 0 bp) to +2 kb from the gene end (proximal 0 bp).

The data show an overall positive correlation between changes in mRNA expression and promoter H3K23Pr in the heart within 1 week of pressure overload. This was evident at the promoters of many upregulated genes (e.g., *Clic1*, *Arrb2*, and *RhoC*; Figure 5, A–C or downregulated genes (Figure 5D), but not those lacking any significant promoter H3K23Pr peaks (Figure 5E) and their mRNA expression (Figure 6, A–E), which persisted for at least 2 weeks after TAC, corroborating those results (Supplemental Figure 6, A–D). Chloride intracellular channel 1 (CLIC1) contributes to the pathogenesis of cancer (28) and Alzheimer’s disease (29). Similarly, both arrestin β2 (ARRB2) and Ras Homolog Gene Family, Member C (RHOC) contribute to cardiac dysfunction when activated or overexpressed (30, 31). The BCAA-free diet consistently reduced TAC-induced increases in the genes’ promoter H3K23Pr (Figure 5, A–C) and their mRNA (Figures 6, A–C).

Conversely, pressure overload induced downregulation of some genes, such as the calcium pump regulator phospholamban (PLN) (32). Accordingly, the decrease in promoter H3K23Pr (Figure 5D) of this gene was associated with downregulation of its mRNA (Figure 6D). However, counterintuitively, the BCAA-free diet curbed the downregulation of both, consistent with overall TAC-induced downregulation of mRNA (Figure 3, D and H), suggesting the involvement of other regulatory factors. Notably, not all genes were regulated by the promoter H3K23Pr. For example, the *Ankrd1* gene, a marker of cardiac hypertrophy that is markedly upregulated, had little or no detectable promoter H3K23Pr peak (Figure 5E), while its mRNA exhibited the highest fold increase after pressure overload, which was equivalent in the hearts of mice on BCAA control and BCAA-free diets (Figure 6E). Similar results were observed with other hypertrophy markers, including *Myh7* (see below) and *Acta1* (Supplemental Figure 7, A, F, and I). The equivalent increases in the hypertrophy markers were congruent with the equal increases in HW/BW at this juncture (1 week after TAC, Figure 2B). Thus, a BCCA-free diet selectively moderated changes in promoter H3K23Pr and gene expression without affecting the increase in cardiac mass, particularly during this early phase of pressure overload.

**Figure 7:**
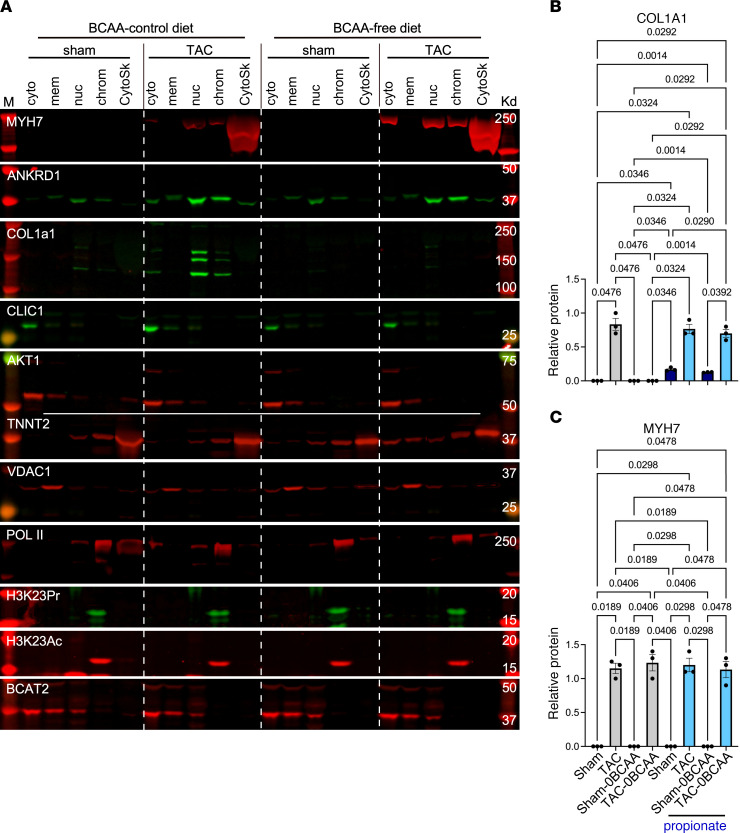
BCAAs differentially regulate protein expression in the hypertrophied heart. Mice were treated as described in Figure 2. (**A**) Protein from the hearts was extracted and fractionated into cytoplasm (cyto), membrane (mem) (which contains mitochondria), nucleoplasm (nuc), chromatin (chrom), and cytoskeletal (CytoSk) fractions. M, standard markers. The fractions were analyzed by WB with the antibodies against the proteins listed on the left of each panel (*n* = 3, each). Note, COL1A1 is assembled in the endoplasmic/sarcoplasmic reticulum, which is in continuum with the nuclear membrane, which is why it is detected in the nuclear fraction. (**B** and **C**) Western blot signals for COL1A1 and MYH7 were quantitated, and the results were graphed as the average relative values, after adjusting one of the sham data points to 1. The results were analyzed by 1-way ANOVA, and the *P* values of those that were 0.05 or less are shown above the brackets encompassing the bars.

**Figure 8:**
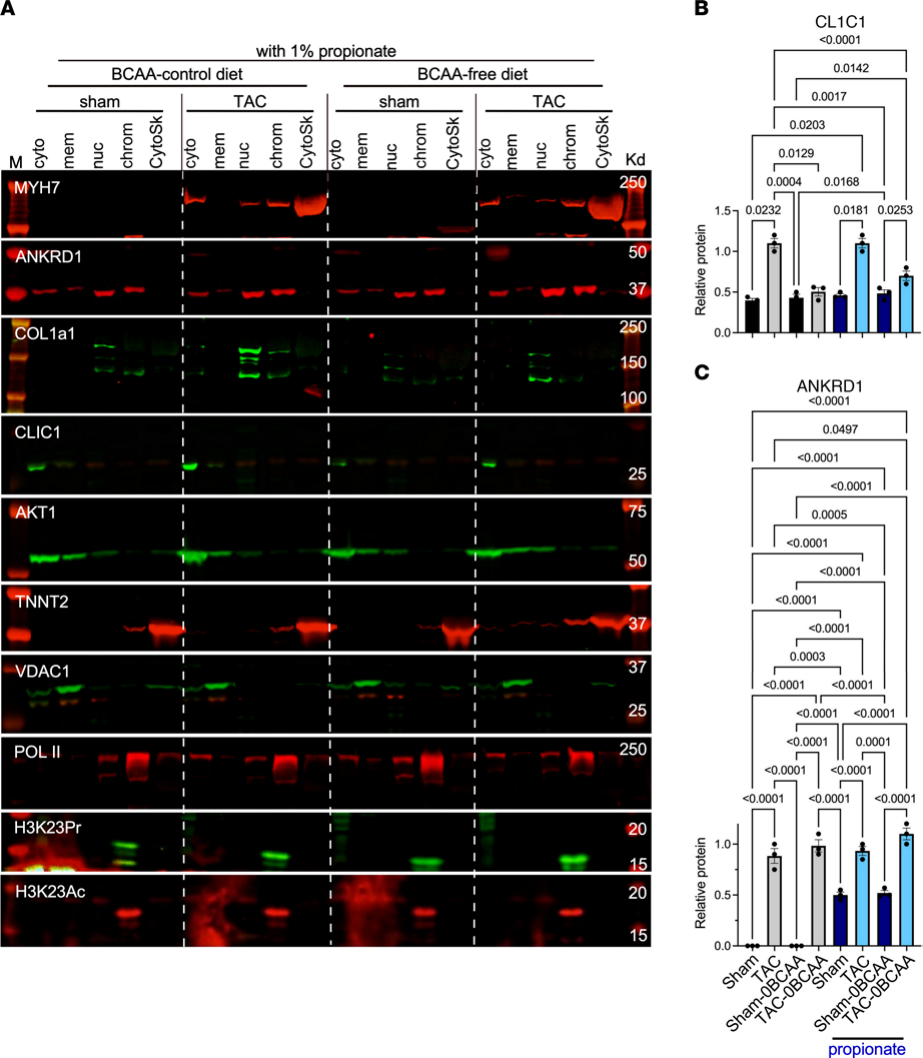
Propionate compensates for the lack of dietary BCAAs’ effect on protein expression. Mice were treated as described in Figure 2, with 1% propionate added to the control or BCAA diets. (**A**) Protein was extracted and analyzed as described in Figure 7A. (**B** and **C**) Western blot signals for CLIC1 and ANKRD1 were quantitated, graphed, and analyzed as described in Figure 7, B and C. The results were analyzed by 1-way ANOVA, and the *P* values of those that were ≤ 0.05 are shown above the brackets encompassing the bars.

In addition to being an intermediate of isoleucine and valine oxidation, propionyl-CoA is also generated from propionate, produced by the gut microbiota (33–35). We confirmed that supplementing a BCAA-free cell culture medium with propionate could dose-dependently increase total lysine-propionyl (K-pr) in histone extracts from cardiac myocytes, as well as specifically increase H3K23Pr content (Figure 6, F and G). Next, to determine whether the effect of BCAA on gene expression in the heart is regulated by histone propionylation-dependent versus -independent mechanisms, we supplemented the mouse diets with 1% propionate, which increased circulating propionate levels approximately 6- to 10-fold (Supplemental Figure 6, E and F). One week after TAC, hearts were analyzed by H3K23Pr ChIP-Seq and RNA-Seq, which revealed that propionate compensated for the lack of BCAAs in the BCAA-free diet by augmenting pressure overload–induced increases in promoter H3K23Pr of upregulated genes (Figure 5, A–C, lower tracks, labeled “with propionate,” and Figure 6H; accession GSE227226, superseries GSE229131), paralleled by an increase in mRNA (Figure 6, A–C; accession GSE227225, superseries GSE229131) and protein expression that was comparable to that seen with the BCAA control diet (without or with propionate [Figure 7, A–C and Figure 8, A–C]). Counterintuitively, however, dietary propionate augmented the reduction of promoter H3K23Pr and RNA expression of downregulated genes during pressure overload (e.g., *Pln*, Figure 5D and Figure 6D), suggesting the involvement of other regulatory factors. Meanwhile, consistent with the above data, propionate had no effect on promoter H3K23Pr abundance (Figure 5E) or on the expression of the mRNA or protein of the hypertrophy marker genes *Myh7* and *Ankrd1* (Figure 6E, Figure 7, A and C, and Figure 8, A and C). Thus, propionate can compensate for the BCAAs needed for TAC-induced upregulation of promoter H3K23 propionylation and the increase in its expression. A complete list of those genes and some of the associated functional pathways are presented in Supplemental Figure 6, K–O.

### A BCAA-free diet reduces pressure overload–induced extracellular matrix proteins in the heart via a propionylation-dependent mechanism.

**Figure 9:**
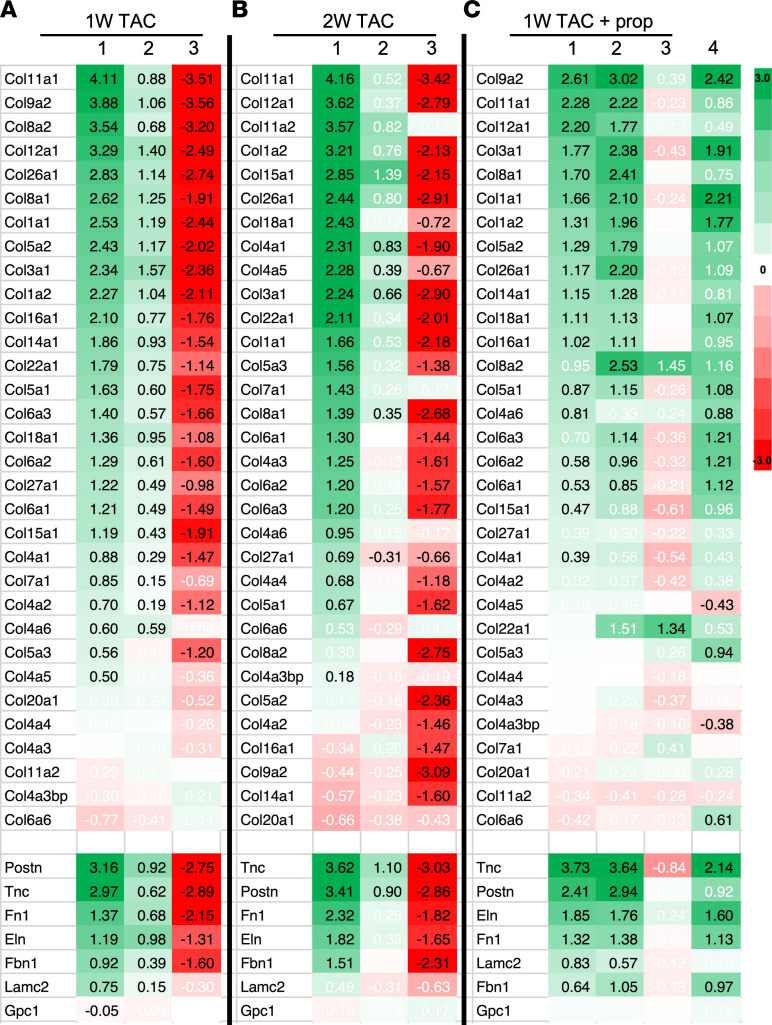
A BCAA-free diet reduces pressure overload–induced ECM genes. Mice were treated, as described in Figure 2, (A and B) without or (**C**) with 1% dietary propionate supplementation. (**A** and **C**) 1 week after TAC or (**B**) 2 weeks after TAC, the hearts were isolated, and RNA was extracted and sequenced (*n* = 3 each). Heatmaps show the LFCs of TAC/sham (column 1, control); TAC/sham (column 2, BCAA-free); TAC (BCAA-free)/TAC (column 3, control); and TAC (column 4, 0BCAA with 1% propionate)/TAC (0BCAA) for all collagen isoform genes expressed in the heart and other ECM genes including *Postn*, *Tnc*, *Fn1*, *Eln*, *Fbn1*, *Lamc2*, and *Gpc1* (negative control; did not increase with pressure overload). The color code bar is shown on the right. Values displayed in black have a padj of 0.05 or less.

**Figure 10:**
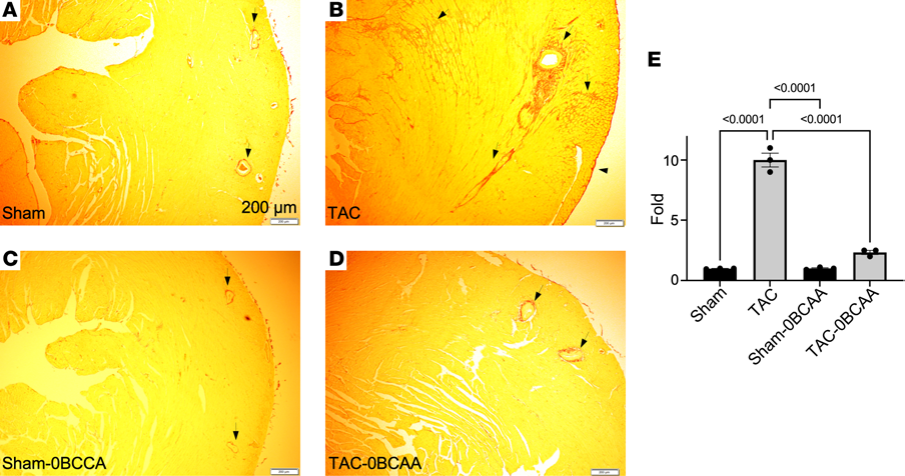
A BCAA-free diet reduces collagen deposition during cardiac hypertrophy. (**A**–**E**) Heart tissue from (**A** and **C**) sham-operated and (**B** and **D**) 2-week TAC hearts from mice on the (**A** and **B**) BCAA control diet or the (**C** and **D**) BCAA-free diet were fixed, sectioned, and stained for collagen using Picrosirius red (*n* = 3 each). Scale bars: 200 μm. (**E**) Collagen (red in the images) was quantitated and graphed as the average relative values, after adjusting one of the sham data points to 1 (*n* = 3). The results were analyzed by 1-way ANOVA, and the *P* values of those that were 0.05 or less are shown on the graph.

Gene set expression analysis revealed that the changes in mRNA expression during TAC with the BCAA control diet were predominantly enriched in gene ontology (GO) terms related to extracellular matrix (ECM) and mitochondrial genes (Supplemental Figure 5, G–J). Western blot analysis confirmed the increase in collagen type I α 1 chain (COL1A1) protein expression in the heart within 1 week of applying pressure overload, which, in contrast, was hardly detectable in the hearts of mice on a BCAA-free diet (Figure 7, A and B). There are several collagen isoforms expressed in the heart, as well as other ECM proteins, including fibronectin, fibrillin, and laminin, that increase during hypertrophy and failure. To determine the extent of ECM regulation, we measured the LFC of the mRNA expression of these collagen isoforms in the heart after 1 week and 2 weeks of imposed pressure overload via TAC versus sham operation in mice maintained on either a BCAA control diet or a BCAA-free diet, as described in Figure 2. The results are presented in a heatmap, where a significant (padj ≤ 0.05) LFC values of TAC/sham for each of the expressed genes is indicated in black font (Figure 9, A and B). After 1 week of pressure overload, 26 of 32 collagen isoforms expressed in the heart were significantly upregulated (Figure 9A, first lane), 24 of which remained persistently high for at least up to 2 weeks after TAC (Figure 9B, first lane) in mice on the BCAA control diet. Also, tenascin C (*Tnc*), periostin (*Postn*), elastin (*Eln*), fibronectin (*Fn1*), laminin C (*Lmnc2*), and fibrillin (*Fbn1*) mRNA were upregulated in those hearts (Figure 9, A and B, first lanes). In contrast, mice maintained on a BCAA-free diet exhibited, overall, a significantly lower LFC for all ECM mRNA after pressure overload that further declined within 2 weeks of TAC (Figure 9, A and B, LFC TAC/sham [BCAA-free], lanes 2). This was also reflected in the lower LFC of TAC (BCAA-free)/TAC (control) (Figure 9, A and B, lanes 3). Staining of heart sections with Picrosirius red confirmed reduced cardiac collagen deposition with the BCAA-free versus BCAA control diet (Figure 10, A–E). Consistent with the results in Figure 8, addition of propionate to the diet compensated for the lack of BCAA in the BCAA-free diet, increasing the LFC of TAC/sham of ECM genes to levels comparable to those observed with the BCAA control diet (Figure 9C, lane 1 vs. lane 2). This was also demonstrated in lane 4 of Figure 9C, which shows the increase in LFC of TAC BCAA-free with propionate and BCAA-free diet. Propionate increased TAC-induced promoter H3K23Pr more robustly with the BCAA-free diet (see Figure 5, A–C), but the levels of mRNA and protein expression did not exceed those observed with the control diets, suggesting that other factors were limiting. Congruently, the increase in HW/BW was comparable with all diets (Supplemental Figure 6G). However, the addition of propionate to either the control or BCAA-free diet adversely affected cardiac function, as reflected by the reduced ejection fraction, which usually does not occur until 4–5 weeks after TAC (Supplemental Figure 6H). On the other hand, propionate did not reverse the effect of the BCAA-free diet on food intake or BW (Supplemental Figure 6, I and J). 

Staining of heart sections with Picrosirius red confirmed reduced cardiac collagen deposition with the BCAA-free versus BCAA control diet (Figure 10, A–E). Moreover, select ECM-regulating factors and receptors, including TGF-β isoforms and its receptors and integrins, conformed with the expression pattern of ECM mRNA and promoter H3K23Pr enrichment (Supplemental Figure 8, A–F), corroborating the effect of diet on ECM. Thus, the data support the fact that promoter H3K23Pr promotes stress-induced ECM gene transcription.

### Lowering BCAA concentrations reduces cardiac fibroblast proliferation and collagen 1a1 expression.

**Figure 11:**
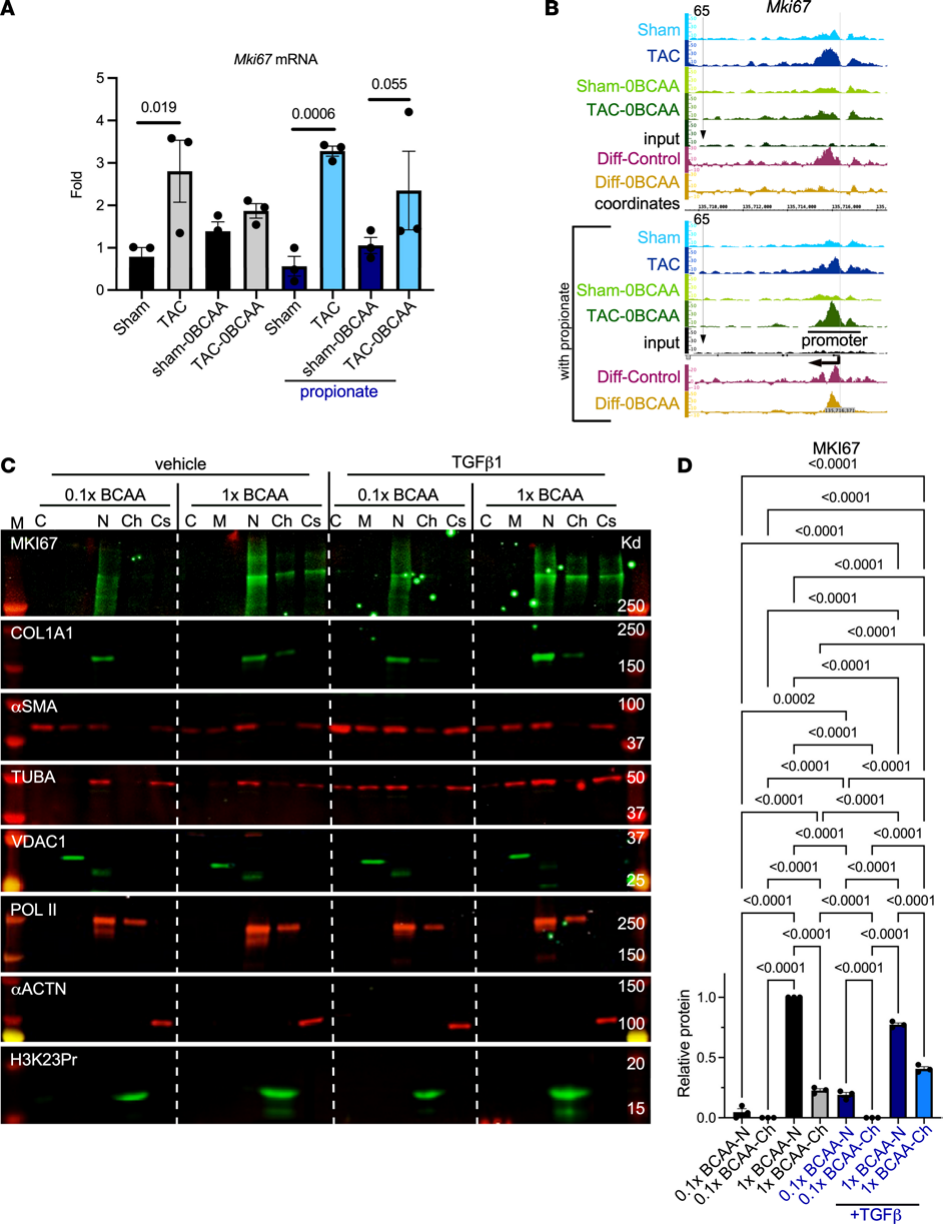
Lowering BCAAs decreases KI67 expression in cardiac fibroblasts. (**A**) Relative fold change of *MKi67* mRNA expression in the heart under the conditions described in the legend to Figure 2 (*n* = 3). Error bars represent the SEM, and the padj (Wald test) of 0.05 or less is given above the lines encompassing the bars. (**B**) IGB images showing H3K23Pr ChIP sequence tags from similarly treated mice, aligned across *Mki67* gene coordinates (data were pooled from of 3 hearts each). See the legend to Figure 4 for more details on the IGB tracks. (**C**) Neonatal rat heart fibroblasts were isolated and cultured. After 20 hours, the medium was replaced with DMEM with standard levels of BCAA (1× BCAA) or low BCAA (0.1× BCAA), without FBS, with 1 ng/mL TGF-β or vehicle, and incubated for another 24 hours (*n* = 3 each). Protein was then extracted and fractionated into cytosol (C), membrane/mitochondria (M), nucleoplasm (N), chromatin-bound (Ch), and cytoskeletal (Cs) fractions and analyzed by WB, with antibodies for the proteins listed on the left in each panel. (**D**) The WB signals were quantified and graphed for MKI67, normalized to POL II. The results were analyzed by 1-way ANOVA after adjusting the signal of the 1× BCAA, vehicle-treated cells, to 1. Error bars represent the SEM and the results were analyzed by 1-way ANOVA. *P* values of 0.05 or less are shown above the brackets encompassing the bars.

**Figure 12:**
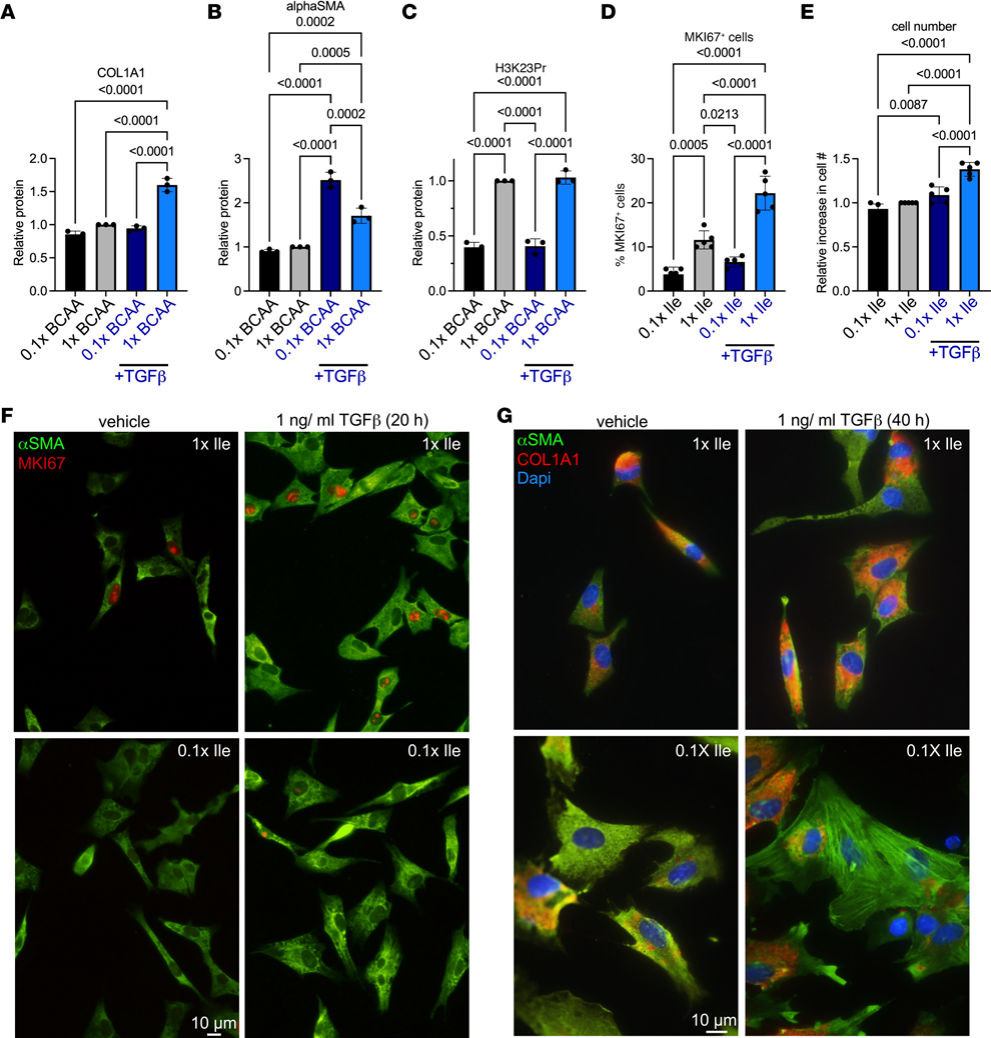
Lowering isoleucine decreases cardiac fibroblast MKI67 and COL1A1 expression. (**A**) Western blot signals in Figure 11C were quantified and graphed for COL1A1 normalized to POL II, (**B**) αSMA normalized to αACTN, and (**C**) H3K23Pr normalized to POL II. The results were as described in Figure 11D. (**D**–**G**) Cardiac fibroblasts were cultured as in described in legend to Figure 11C. After 20 hours, the medium was replaced with DMEM with either standard levels of Ile (1× Ile) or low Ile (0.1× Ile), without FBS, with 1 ng/mL TGF-β or vehicle, and incubated for 20 hours (*n* = 5 each). (**D**) The percentage of MKI67^+^ cells was counted, and (**E**) cell numbers were calculated in 4 fields for each of the 5 repeats, graphed after adjusting the 1× BCAA- or vehicle-treated cells to 1, and analyzed by 1-way ANOVA. (**F**) Cells were then immunostained with anti-MKI67 (red) and anti-αSMA (green), and imaged. Scale bar: 10 μm. Original magnification, ×40. (**G**) Similarly, fibroblasts were treated with 1 ng/mL TGF-β or vehicle for 40 hours (*n* = 3 each). The fibroblasts were then immunostained for anti-COL1A1 (red) and anti-αSMA (green) antibodies and imaged. Scale bar: 10 μm. Original magnification, ×60.

MKI67 is one of the most established proliferation markers (36) and is invariably upregulated in proliferating fibroblast (37). Our RNA-Seq data show that *Mki67* mRNA was significantly upregulated in the hypertrophied heart, coinciding with an increase in its promoter H3K23Pr, and that both of these were suppressed by a BCAA-free diet and reversed by supplementation of the diet with propionate (Figure 11, A and B). Western blot analysis of protein extracts from cardiac fibroblasts shows that MKI67 was more robustly expressed in cells cultured with standard levels of BCAA (1× BCAA) versus reduced BCAA (0.1× BCAA) and increased in the chromatin fraction of the former upon stimulation with TGF-β for 24 hours (Figure 11, C and D). This was corroborated by immunostaining of fibroblasts maintained in the presence of low (0.1×) versus standard levels (1×) of Ile (Figure 12, D and F), consistent with the fact that Ile is responsible for histone propionylation (23). This was associated with an increase in cell numbers (Figure 12E). The finding was also reproduced in cells stimulated with endothelin-1 (Supplemental Figure 9A). These results suggest that reduced ECM gene expression by a BCAA-free diet was at least partly due to reduced fibroblast proliferation after pressure overload.

COL1A1 increased after TGF-β stimulation in the presence of the standard 1× BCAA medium, but not the 0.1× BCAA medium (Figures 11C and Figure 12A). On the other hand, α smooth muscle actin (αSMA) was induced by TGF-β in the presence of both 1× and 0.1× BCAA medium, with significantly higher expression levels in the latter (Figure 11C and Figure 12B). Immunostaining confirmed that TGF-β in the presence of 0.1× Ile versus 1× Ile medium promoted the assembly of αSMA fibers (Figure 12G, left panels), which is characteristic of fully differentiated myofibroblasts (38). These cells expressed little or no COL1A1 (Figure 12G, lower left panel), consistent with a report by Tsukui et al., which established this inverse relation by single-cell RNA-Seq analysis (39). Thus, lower supplies of Ile (0.1×), associated with reduced levels of H3K23Pr (Figure 12C), reduced fibroblast proliferation and collagen expression, while increasing αSMA expression and fiber assembly.

### A BCAA-free diet enhances basal and spare mitochondrial respiration.

**Figure 13:**
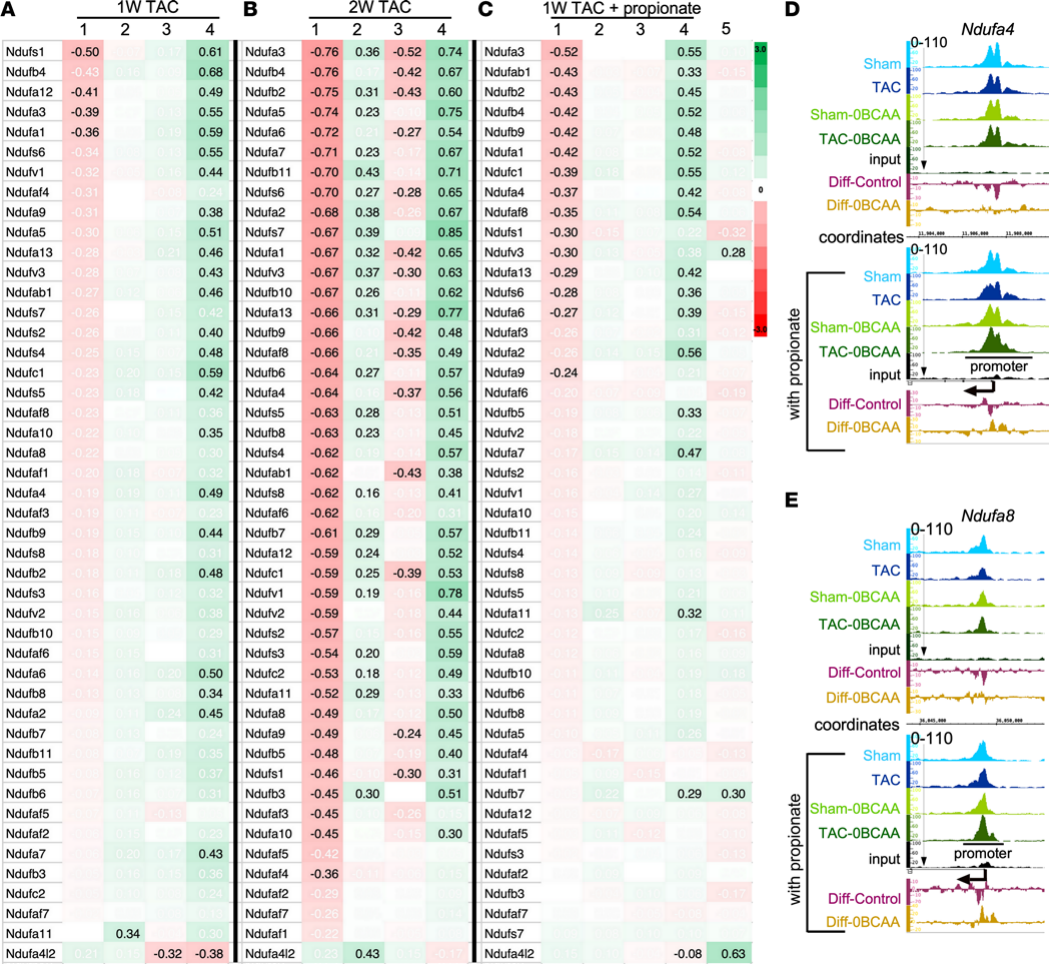
A BCAA-free diet prevents pressure overload–induced downregulation of ETC subunits. As described in Figure 2, mice were treated (**A** and **B**) without or (**C**) with 1% dietary propionate supplementation. (**A**) One week or (**B**) 2 weeks after TAC, the hearts were isolated, and RNA was extracted and sequenced (*n* = 3 each). Heatmaps show the LFC of TAC/sham (column 1, control); TAC/sham (column 2, BCAA-free); sham (BCAA-free)/sham (column 3, control); TAC (BCAA-free)/TAC (column 4, control); TAC (0BCAA with 1% propionate)/TAC (column 5, 0BCAA). (**D** and **E**) IGB images showing H3K23Pr ChIP sequence tags from the sham-operated or TAC hearts of mice on the different diets with or without propionate supplementation, aligned across the *Nduaf4* and *Ndufa8* gene coordinates. Values displayed in black have a padj of 0.05 or less. See the legend to Figure 5 for more details on the IGB tracks.

Pressure overload on the heart induced the downregulation of metabolic (Supplemental Figure 10) and ETC I–V subunits (Figure 13 and Supplemental Figure 11). With a downward trend within 1 week, the decline in gene expression was significant 2 weeks after TAC (Figure 13, A and B, and Supplemental Figure 11, A and B, first lanes). This was not a consequence of reduced mitochondrial volume, since mRNA expression of the translocase membrane proteins (*Timm* and *Tomm*) and expression of total mitochondrial protein/mg tissue remained unchanged (Supplemental Figure 12). The reduction in mRNA expression was accompanied by a decrease in promoter H3K23Pr levels (Figure 13, D and E). Intriguingly, the BCAA-free diet prevented the decline in ETC gene expression and promoter H3K23Pr levels within 1 week of pressure overload (Figure 13, D and E) and induced a significant increase in the expression of most of the subunits after 2 weeks of TAC (Figure 13B, middle lane, and Supplemental Figure 9B, middle lane). Supplementing the diet with propionate did not reverse or lessen downregulation of the ETC genes in mice on the BCAA control diet, except for complex II (succinate dehydrogenase, also a TCA cycle enzyme), which, on the contrary, augmented the decrease in the expression of these genes (Figure 13C). On the other hand, the addition of propionate to the BCAA-free diet had no effect on the expression of ETC genes (Figure 13C, lanes 2 and 5, and Supplemental Figure 11C), while it slightly increased promoter H3K23Pr expression (Figure 13, D and E).

**Figure 14:**
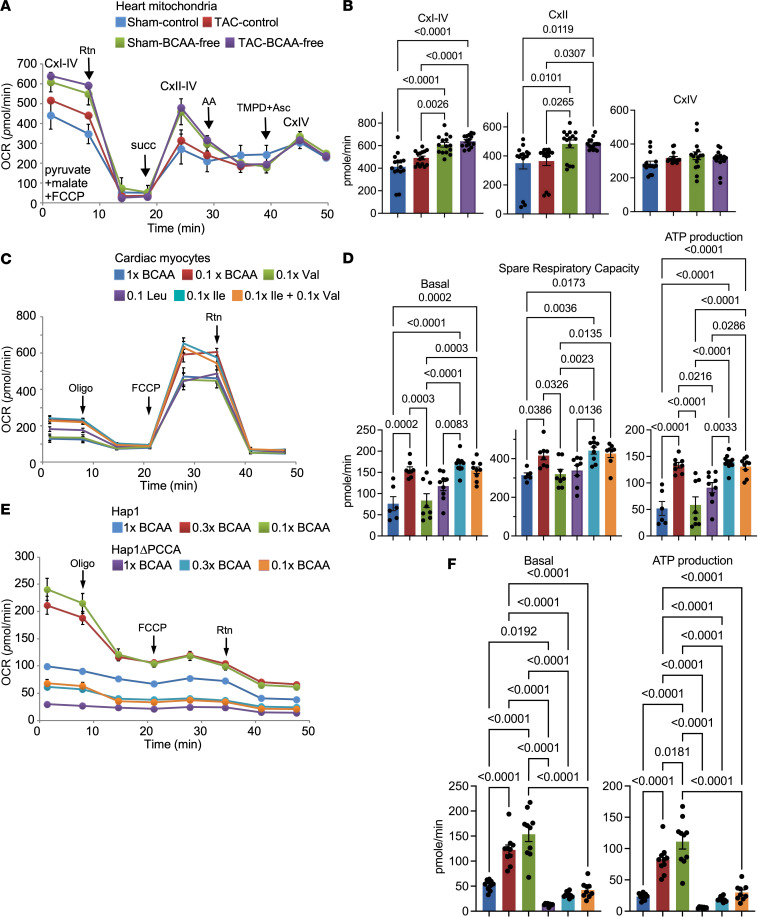
Lowering BCAAs or isoleucine enhances mitochondrial respiration. (**A**) Mitochondria were freshly isolated from the heart, and the OCR (pmol/h, *y* axis) over time (*x* axis) was measure using the Seahorse analyzer, before and after the addition of rotenone (Rtn), succinate (succ), antimycin A (AA), and TMPD plus ascorbic acid (TMPD+Asc), at the time points indicated by the arrows on the curve. (**B**) Basal (CI–IV), CxII, and CxIV OCRs (pmol/h) were graphed (*n* = 3 independent hearts; *n* = 15 replicas each) after normalization to mitochondrial protein. Error bars represent the SEM. *P ≤ 0.05, by 1-way ANOVA. (**C**) Neonatal rat cardiac myocytes were cultured in DMEM (with glucose, without FBS) with either 1× or 0.1× BCAAs, or with 0.1× of the individual aa Leu, Ile, or Val, as indicated by the color key, without FBS. After 16 hours, the OCR (pmol/h, *y* axis) versus time (h, *x* axis) was measured using the Seahorse analyzer, before and after the addition of oligomycin (Oligo), FCCP, and rotenone, at the time points indicated by arrows. (**D**) The mitochondrial spare respiratory capacity, proton leak, and ATP-linked OCRs were calculated and graphed (*n* = 3 independent cultures; *n* = 6–10 replicas each). Error bars represent the SEM. *P ≤ 0.05, by 1-way ANOVA. (**E**) Hap1 and Hap1ΔPCCA were cultured in DMEM with increasing doses of BCAAs as indicated by the color key, without FBS. After 16 hours, the OCR was measured (*n* = 3 independent cultures; *n* = 10 replicas each), as described in **H**. (**F**) The data were graphed and analyzed by 1-way ANOVA, as described in **D**.

To investigate whether the changes in ETC gene expression translate into an effect on mitochondrial respiration, we freshly isolated mitochondria from hearts of the mice on the different diets and stress conditions described above and measured their oxygen consumption rates (OCR) in the presence or absence of rotenone (inhibitor of CxI), succinate (activator of CxII), antimycin (inhibitor of CxIII), or TMPD plus ascorbic acid (activator of CxIV). The results show that basal levels (CxI-IV) and spare respiratory capacity (CxII-IV), which we have previously shown to be a function of CxII (40), were significantly higher in mitochondria isolated from hearts of mice on a BCAA-free versus those on a BCAA control diet, in both sham-operated and TAC hearts (Figure 14, A and B).

Next, to determine whether BCAAs directly regulate ETC expression and function, we measured the OCR in live cultures of neonatal myocytes. The most notable differences between BCAA doses were observed with spare respiratory capacity and ATP production, in which 0.1× BCAA versus 1× BCAA induced significantly higher levels of OCR (Figure 14, C and D). Although the latter measurements were in the presence of glucose, we obtained similar results with cells maintained on palmitate-BSA, glucose-free medium (Supplemental Figure 13, C and D). To determine which BCAA was responsible for the observed effects on the OCR, we maintained cardiac myocytes in media with 0.1× of each one’s standard DMEM concentration, separately. The results show that only the medium with 0.1× Ile conferred the increases in basal, reserve, and ATP-linked OCR (Figure 14, C and D).

To confirm these findings and determine their relevance in human cells, we examined the effects of BCAAs on mitochondrial respiration in human Hap1 cells and in Hap1 cells with a deletion of PCCA (Hap1ΔPCCA), rendering it defective in the carboxylation of propionyl-CoA to methylmalonyl-CoA and inducing an increase in histone propionylation (Figure 1, C and D). The results revealed that when Hap1 cells were cultured in low BCAAs (0.1× and 0.3×), they exhibited approximately 2-fold higher basal OCR levels and approximately 4-fold higher ATP production levels versus culturing them in medium with standard BCAAs (1× BCAA, Figure 14, E and F). The knockout of PCCA lowered these parameters to levels below those observed with 1× BCAA in the parent cells, as expected from its effect on histone propionylation (Figure 1, C and D). These results confirm that lowering BCAAs in the medium enhanced mitochondria respiration in cultured human cells.

## Discussion

Our results show that a diet with standard concentrations of BCAAs (BCAA control) was associated with selective increases in H3K23Pr at the promoters of genes that are upregulated by pressure overload on the heart. A notable category of genes regulated by BCAAs are those of the ECM, including collagen isoform, fibronectin, laminin, and fibrillin genes (Figure 9, A–C and Supplemental Figure 8), which play a critical role in the pathogenesis of heart failure. Another major category of genes with a similar pattern of expression includes the cluster-of-differentiation (CD) surface markers expressed in infiltrating immune cells, e.g., CD72, expressed on all B cells (41), and Cd84, expressed on lymphocytes and monocytes (41) (Supplemental Figure 14, A–C). Since ECM and immune cell CD genes are mainly expressed in myofibroblasts and infiltrating immune cells, respectively, it is plausible that an increase in cell number contributes to the increase in gene expression and promoter H3K23Pr. Indeed, we show that higher BCAA concentrations enhanced cardiac fibroblast proliferation, expression of the proliferation marker KI67 and of COL1A1, which, conversely, were inhibited by lowering BCAA levels (Figures 11 and 12). To our knowledge, this is the first study to demonstrate that lowering dietary BCAAs can substantially reduce fibroblast proliferation and ECM expression associated with pressure overload via modulation of the cells’ epigenetics, which might have significant clinical implications.

Another striking difference between the effect of the BCAA-free diet versus the BCCA control diet in the hypertrophied heart was the ability of the BCAA-free diet to prevent the downregulation of ETC I–V gene expression during cardiac hypertrophy, i.e., it preserved their normal/basal levels (Figure 13A and Supplemental Figure 11). Unlike the other early (within 1 week) changes in gene expression, ETC downregulation by pressure overload had a more gradual onset that only reached significance after 2 weeks of pressure overload. Moreover, supplementing the diet with propionate did not counteract the effect of the BCAA-free diet. Conversely, this supplementation accelerated the effect of the BCAA control diet, as it exhibited a significant decline in ETC gene expression within 1 week of pressure overload. This suggests that the effects of BCAAs on the expression of ETC genes may be independent of promoter propionylation. One possibility is that these genes may be regulated by RNA-binding proteins (YBX1-3, Supplemental Figure 13E), or, alternatively, the time frame of our experiment may not have been long enough. Contradictorily, propionate inhibited the OCR in cultured cardiac myocytes, suggesting that an effective concentration may be different in monolayer cultures versus in vivo.

We confirmed that serum BCAA levels were reduced (~60%) in mice consuming a BCAA-free diet (Figure 2A). However, we did not measure the levels of propionyl-CoA in the heart under these conditions. Thus, we can conclude that dietary BCAA concentrations influence promoter H3K23Pr levels of inducible genes, but we cannot conclude that this was a direct effect of the production of the BCAA intermediate propionyl-CoA in the nucleus. We can infer it, however, from the study by Trefely et al., which shows that propionyl-CoA is substantially enriched in the nucleus versus the cytosol, is derived from isoleucine oxidation, and contributes to H3K23 propionylation (23). It has also been reported that the propionyl-CoA carboxylase is “slightly reversible” with a low propionyl-CoA flux (42), plausibly a negative feedback loop counterbalancing reduced BCAA levels and meriting further investigation.

Intriguingly, the BCAA-free diet did not significantly change basal levels of promoter H3K23Pr or gene expression in the normal (i.e., unstressed heart), at least within the time frame of our experiments (1 week–2.5 weeks). This could be explained by the fact that the gut microbiome produces sufficient propionate and BCAAs to maintain basal levels of histone propionylation. In support of this, we show that the levels of circulating BCAAs were only reduced by 60% with the BCAA-free diet (Figure 2A) and that the mouse gut harbored *Firmicutes* and *Bacteroidetes,* which increased in abundance with a BCAA-free diet (Supplemental Figure 3). Alternatively, propionyl-CoA can be derived from odd-chain fatty acid, which was lacking in the mouse diets, but can be synthesized by the gut microbiome (7). We did predict, however, that by eliminating dietary BCAAs, the overall reduction in the availability of BCAAs would diminish the pressure overload–induced increase in cardiac mass (HW/BW). This was not the case after 1 week of pressure overload (Figure 2C), however, after 2 weeks of TAC, we observed a plateauing of the HW/BW, but a continued rise in this ratio with the BCAA control diet (Figure 2C). This finding could be explained by insufficient BCAA levels, which are necessary for myocyte growth, reduced collagen expression and deposition (Figures 9 and 10 and Supplemental Figure 9), and/or reduced expression of factors required for cardiac growth. For example, while endothelin-3 (Edn3), which is involved in the development of cardiac hypertrophy (43, 44), was similarly upregulated in the hearts of mice on either diet, 1 week after TAC, Edn3 expression levels began to decline in the absence of dietary BCAAs (Supplemental Figure 15). Meanwhile, the BCAA control diet was associated with additional increases in Edn1 and endothelin receptor 1b (Ednrb). Alternatively, lower dietary leucine, which has been shown to enhance protein translation initiation (45) and protein synthesis in the heart (46), may play a role in dampening cardiac hypertrophy. Further studies with individual BCAA-deficient diets are underway to better characterize the roles of BCAAs in gene expression and cardiac hypertrophy.

Little is known about H3K23Pr or its role in gene transcription. It correlates with active transcription and is regulated by the acetyl-CoA/CoA ratio in C2C12 myoblasts (47). H3K23Pr is also downregulated in testes of mice on a high-fat diet, associating with low sperm motility (48). We show here that H3K23Pr was enriched at the promoters of all constitutively expressed genes, as well as at inducible promoters, albeit selectively (Figure 5). During pressure overload on the heart, the changes in promoter H3K23Pr positively correlated with a gene’s change in mRNA levels (Figure 4). Moreover, these changes were modulated by dietary BCAAs (Figures 2–5). Yan et al. reported the association of H3K23 with the KAT6A-KT6B-BRPF1 complex, which catalyzes its propionylation (49). Our RNA-Seq data show that both *Kat6a* and *Kat6b* mRNA were significantly downregulated (25%–27%) within 1 week of pressure overload in the hearts of mice on a BCAA-free diet (Supplemental Figure 16A). This suggests a positive feedback loop associated with a reduced requirement of KAT6A/B as BCAAs levels were decreased. We also confirmed that knockout of Kat6a was associated with reduced levels of H3K23Pr (Supplemental Figure 16, C and D).

H3K23Pr is one of many histone modifications that differentially affects the recruitment of transcription factors and regulators to a gene’s promoter. Indeed, other histone modifications have been shown to regulate gene expression during cardiac hypertrophy (50–52). Therefore, the observed effect of BCAAs on gene expression may not always directly correlate with the level of dietary BCAAs or the increase in histone propionylation. Notable examples of this include the cardiac-specific sarcomeric genes, in which 1 week after TAC, we observed a reduction in promoter H3K23Pr of the sarcomeric cardiac actin gene, among others, which was not associated with a change in the gene’s mRNA levels with the BCAA control diet and which consistently increased with the BCAA-free diet (Supplemental Figure 7, A and D). After 2 weeks of TAC, we found that both the BCAA control and BCAA-free diets were associated with an increase in promoter H3K23Pr levels, whereas the mRNA levels remained equivalent to those seen 1 week after TAC (Supplemental Figure 7, B–G). The data would suggest that the source of propionyl CoA — dietary versus gut microbiome — selectively regulates H3K23Pr abundance. This could be attributed to the selective binding of the BCAA oxidation enzymes versus the acyl-CoA synthetase short-chain family member to chromatin. Other explanations include an increase in propionyl transferase activity or a decrease in depropionylase activity at these promoters, triggered by low BCAA levels. Note that the established hypertrophy marker genes *Ankrd1* and sarcomeric α actin (*Acta1*) had little or no promoter H3K23Pr and were, accordingly, equally upregulated in the hypertrophied heart with both diets, thus serving as a positive internal control (Figure 5E, Figure 6E, and Supplemental Figure 7, A and F).

In summary, this study shows that a diet deficient in BCAAs was beneficial during cardiac stress by depressing stress-induced changes in promoter H3K23Pr levels and the correlating changes in gene expression. Specifically, low BCAA levels reduced KI67, myofibroblast proliferation and collagen expression and deposition during cardiac hypertrophy. Conversely, BCAAs completely blocked the downregulation of ETC gene expression during cardiac hypertrophy, thereby boosting respiration.

## Methods

### Animals, diets, and animal care.

Ten- to 12-week-old male C57BL/6J mice were purchased from The Jackson Laboratory, as needed. Mouse diets were purchased from Research Diets, including a custom-made BCAA control diet (catalog A12450K), a BCAA-free diet (0BCAA, catalog A19121601), a BCAA control diet with 1% propionate (catalog A22041404), and a BCAA-free diet with 1% propionate (catalog A22041405, see Supplemental Table 1 for the formulas). Sprague-Dawley dams with 1- to 2-day-old pups were purchased from Charles Rivers Laboratories.

### Human haploid and Hap1-knockout cell lines.

Human haploid (Hap1), Hap1Δ BCAA transferase 2 *(*Δ*BCAT2*), and Hap1Δ propionyl-CoA carboxylase A (Δ*PCCA*) cell lines were purchased from Horizon Discovery. Cells were cultured in DMEM with 10% FBS. The medium was changed to DMEM with or with BCAAs, without FBS, for the experiments in Figures 1 and 8. These were fibroblast-like cells derived from the human male chronic myelogenous leukemia (CML) cell line KBM-7. The knockouts were generated by CRISPR/Cas9 editing of the genes. Hap1Δ*BCAT2* was generated by a 13 bp deletion in exon 5, with the target transcript BCAT2 (NM_001190) and the following RNA guide sequence: GACTGGGTCCCCGATGCCGC. Hap1Δ*PCCA* was generated by a 92 bp deletion in exon 5, with the target transcript PCCA (NM_000282), and the following guide RNA sequence: TTGAGGTAGCTTTTACTGGT.

### Culturing of neonatal rat cardiac myocytes.

Cardiac myocytes were cultured as described in our previous reports (53). Briefly, hearts were isolated from 1-day-old Sprague-Dawley rats. After dissociation with collagenase, cells were subjected to Percoll gradient centrifugation followed by differential preplating for 30 hours to enrich for cardiac myocytes and deplete nonmyocytes. Myocytes were cultured in DMEM/F12 plus 10% FBS.

### Culturing of neonatal rat cardiac fibroblasts.

Cardiac fibroblasts were isolated from the same hearts used for isolation of cardiac myocytes, applying the same extraction method explained above. Cardiac fibroblasts were separated from the cardiac myocytes by Percoll gradient centrifugation. The cells were then counted and cultured in either 10 cm plates or gelatin-coated glass slides in DMEM with 10% FBS. The next day, the medium was replaced with customized media (deficient in BCAAs or individual aa), without FBS, with or without 1 ng/mL TGF-β (Abcam, catalog ab50036) for 24 or 48 hours, as indicated. The BCAA-free DMEM is a custom-made medium from Thermo Fisher Scientific (SKU: ME20061L1), which is a modification of SKU: 11966 DMEM (see Supplemental Table 2 for the formula).

### Recombinant modified nucleosomes, subcellular fractionation, histone extraction, and Western blotting.

Recombinant nucleosomes with the modified histones H3K23Ac (catalog 16-0364)and H3K23Pr (custom-made) were purchased from EpiCypher and analyzed by Western blotting (WB) with the corresponding antibodies. Information regarding the antibodies used in this manuscript is provided in Supplemental Table 3. See complete unedited blots in the supplemental material.

Cellular protein (25–50 μg) was fractionated using the subcellular protein fractionation kit (Thermo Fisher Scientific, catalog 78840) according to the manufacturer’s protocols. Histones were extracted using EpiGentek’s EpiQuik Total Histone Extraction Kit (catalog OP-0006-100).

The cellular fractions were separated on a 4%–12% gradient SDS-PAGE (Criterion gels, Bio-Rad) and transferred onto nitrocellulose membranes. Western blot signals were detected by the Odyssey imaging system (LI-COR) and quantitated using ImageJ (NIH).

### Measurement of plasma BCAA levels.

Blood was drawn from the left ventricle of mice using a heparinized needle. Plasma BCAAs were measured using Abcam’s BCAA assay kit (catalog 83374) according to the manufacturer’s protocol.

### Measurement of plasma propionate concentrations.

Blood was drawn from the left ventricle of mice using a heparinized needle. Measurement of SCFAs was performed by Creative Proteomics. The protocol and results are shown in Supplemental Figure 6, E and F.

### Immunocytochemistry.

Cells were cultured in gelatin-coated 4-well glass chambers. Cells were fixed in 4% paraformaldehyde with 0.3% Triton X-100. They were then incubated with the designated antibody in Tris-buffered saline with 1% BSA and then washed and mounted using Prolong Gold antifade with DAPI (Thermo Fisher Scientific, catalog P36931). 

### Mitochondria isolation, electron flow assay, and stress test.

For the mitochondrial electron flow assay, mitochondria were isolated from heart tissue using differential centrifugation in a buffer composed of 70 mM sucrose, 210 mM mannitol, 5.0 mM HEPES, 1.0 mM EGTA, and 0.5 % (w/v) fatty acid–free BSA, pH 7. Using 40 μg mitochondria, the assay was conducted in a buffer consisting of 70 mM sucrose, 220 mM mannitol, 10 mM KH2PO4, 5 mM MgCl_2_, 2 mM HEPES, 1.0 mM EGTA, 0.2 % (w/v) fatty acid–free BSA, pH 7.2, with 10 mM pyruvate, 2 mM malate, and 4 μM FCCP. Using the Seahorse XF^e^96 Analyzer, basal OCRs were measured before and after sequential injections of 2 μM rotenone, 10 mM succinate, 4 μM antimycin A, and 10 mM ascorbate plus 100 μM tetramethyl-*p*-phenylene diamine (TMPD), final concentrations.

The mitochondrial stress assay was performed as we previously described (54). Cardiac myocytes, Hap1 cells, or Hap1ΔPCCA cells were seeded in 96-well Seahorse analyzer plates (50,000 cells/well) in full medium consisting of DMEM plus 10% FBS overnight (~20 hours). The medium was then changed to DMEM with a varying concentration of BCAAs (from 0- to 2-fold the levels present in DMEM) along with 17.5 mM glucose or 100 μM palmitate-BSA, as indicated in the figure legends. Using the Seahorse XF^e^96 Analyzer, basal OCRs were measured in live cells, followed by measurements after the sequential addition of 1 μM oligomycin 8 hours after the start of measurements, 1 μM FCCP at 22 hours, and 10 μM antimycin A plus rotenone at 35 hours. Two readings were taken after each compound was injected, and the results were plotted in real time in pmol/hours versus time (hours) (40). The raw data were then exported to an Agilent Seahorse XF Mito Stress Test Report Generator file for calculation and graphing of mitochondrial basal levels, spare respiratory capacity, proton leak, and ATP production.

### TAC in mice.

TAC was performed as described in our previous reports (55, 56). Briefly, a 7-0 braided polyester suture was tied around the transverse thoracic aorta, against a 27 gauge needle, between the innominate artery and the left common carotid artery. Control mice were subjected to a sham operation involving the same procedure, minus the aortic constriction.

### Echocardiography and Doppler measurements.

Echocardiographic and Doppler measurements were performed as described in our previous reports (55, 56). Briefly, transthoracic echocardiography was performed using the Vevo 3100 imaging system (Visual Sonics) with a MX400 30 MHz (mouse, cardiology) scan head encapsulated transducer. Electrocardiographic electrodes were taped to the 4 paws, and then 1D M-mode and 2D B-mode tracings were recorded from the parasternal short-axis view at the mid-papillary muscle level. In addition, pulse-wave Doppler was used to measure blood flow velocity and peak gradient pressure in the aorta. Vevo 3100 Software (Vevo Lab, version 3.2.6) was used, which includes an analytic software package for B-Mode (2D) image capture and analysis; cine loop image capture, display, and review; software analytics for advanced measurements and annotations; and physiological data onscreen tracing.

### ChIP-Seq analysis.

Mouse heart tissue was analyzed by ChIP (performed by Active Motif) using anti-H3K23Pr (Abcam, catalog ab2414466). Briefly, ChIP libraries (~200 bp average fragment length) were sequenced using Illumina’s NextSeq 500, generating 75 nt single-end sequence reads that were mapped to the genome using BWA algorithms. The reads/tags were extended in silico by 200 bp at their 3′ end (fragments), the density of which was determined along the genome and divided in 32 nt bins, and the results were saved in bigWig and BAM (Binary Alignment/Map) files, which were used for the plots, heatmaps, and images of the fragments aligned to chromosome coordinates. Fragment peaks were identified using MACS (57), which identifies local areas of enrichment of Tags when compared with input, defined as “intervals,” while overlapping intervals were grouped into “merged regions.” The locations and proximities to gene annotations of intervals and active regions were defined and compiled in Excel spreadsheets, which include average and peak fragment densities.

Additionally, we analyzed the fragment densities by gene region, where the average value (Avg Val) of fragment densities at the transcription start site (TSS) (–1,000 to +1,000) and gene bodies (+1,000 to 3′ end) regions for all genes was calculated separately. Furthermore, these results were integrated with those of the RNA-Seq data, which were used to sort the genes into those that were upregulated, downregulated, or unchanged after TAC.

### RNA-Seq analysis.

Using the Illumina NextSeq 500, 42 nt sequence reads were generated, which were aligned with the genome using the STAR algorithm (RNA-Seq was performed by Azenta Life Sciences). Read pairs that had both ends aligned were counted, and those with at least 25 bp overlapping bases in a fragment were assigned. Gene annotations were obtained from the Subread package (58). Differential genes were detected by DESeq2 at a padj 10% FDR (59).

### ChIP-Seq and RNA-Seq analysis software.

Integrated Genome Browser (IGB) (60) can be downloaded for free at http://bioviz.org/igb/

### Statistics.

One-way ANOVA with post hoc Tukey’s or multiple-comparison analysis or 1-way ANOVA with Šídák’s multiple-comparison test was used to calculate significance between more than 2 groups. A Student’s *t* test (equal variance, 2-tailed) was used to calculate significance between 2 groups, and a *P* value of 0.05 or less was considered significant. For RNA-Seq data, differential gene expression (LFC) was detected by DESeq2 at a padj 10% FDR (59). The Wald test was used to calculate *P* values and LFCs. LFCs with a padj of 0.05 or less were considered specific, as represented in Figures 4, 6, 7, 9, and 13. RNA-Seq analysis was performed by Azenta Life Sciences.

### Study approval.

All animal procedures in this study were performed in accordance with the NIH *Guidelines for the Care and Use of Laboratory Animals* (National Academies Press, No. 85-23, 2011). All protocols were approved by the IACUC of the Rutgers-New Jersey Medical School.

### Data availability.

All raw and processed RNA-Seq and ChIP-Seq data are deposited in the NCBI’s Gene Expression Omnibus (GEO) database in a superseries (accession GSE229131). The superseries includes 6 data sets (3 for the RNA-Seq data and 3 for the ChIP-Seq data) under GEO accessions GSE227110, GSE227226, GSE227227, GSE227228, GSE227225, and GSE229128. Other data values are provided in the Supplemental Supporting Data Values file. All data are also available from the corresponding author upon request.

## Author contributions

ZY performed all surgical procedures and echocardiography on the mice and extracted RNA from heart tissue. MH cultured cardiac myocytes and fibroblasts and performed mitochondrial stress tests on cardiac myocytes (Seahorse analysis). JA cultured Hap1 cells, fractionated cellular protein extracts from heart tissue, performed WB and immunocytochemistry, and measured OCRs on isolated mitochondria and Hap1 cells (Seahorse analysis). DS performed ChIP-Seq analysis. MA designed and supervised all experiments, analyzed data, generated figures, and wrote the manuscript.

## Supplementary Material

Supplemental data

Supporting data values

## Version 1 09/05/2023

In-Press Preview

## Version 2 11/15/2023

Electronic publication
