# Supplementary material for: Reducing branched-chain amino acids improves cardiac stress response in mice by decreasing histone H3K23 propionylation
Source: J Clin Invest. 2023 Nov 15;133(22):e169399. doi: 10.1172/JCI169399 (PMC10645387; doi:10.1172/JCI169399)

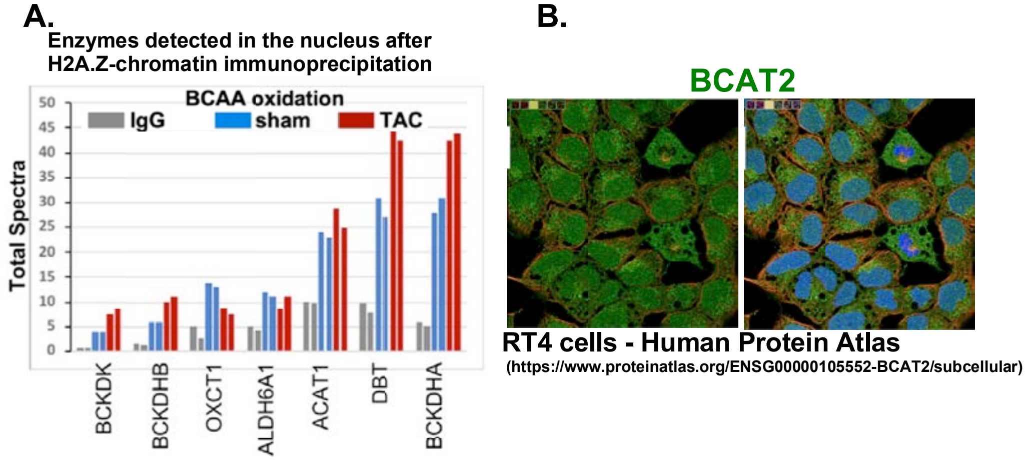

Figure 1S. BCAA oxidation enzymes are detected in the nucleus. A. Mice were subjected to either a sham operation or transverse aortic constriction (TAC) for 1 week. Chromatin was extracted and analyzed by anti-H2A.Z immunoprecipitation followed by mass spectrometry. This identified BCAA oxidation enzymes in the nucleus. The graph shows the total spectra identified for each of the indicted enzymes in the control IgG (grey bars), the sham heart (blue bars), and the hearts subjected to TAC (red bars). Note, not all the BCAA oxidation enzymes were identified by this approach, as their abundance and strength of association with chromatin influences their detection. **B.** Immunofluorescence staining of BCAT2 (green), nuclei (blue), microtubules (red), in human RT4 cells, showing its mitochondrial as well as nuclear localization (Human Protein Atlas, see link to webpage below the image).

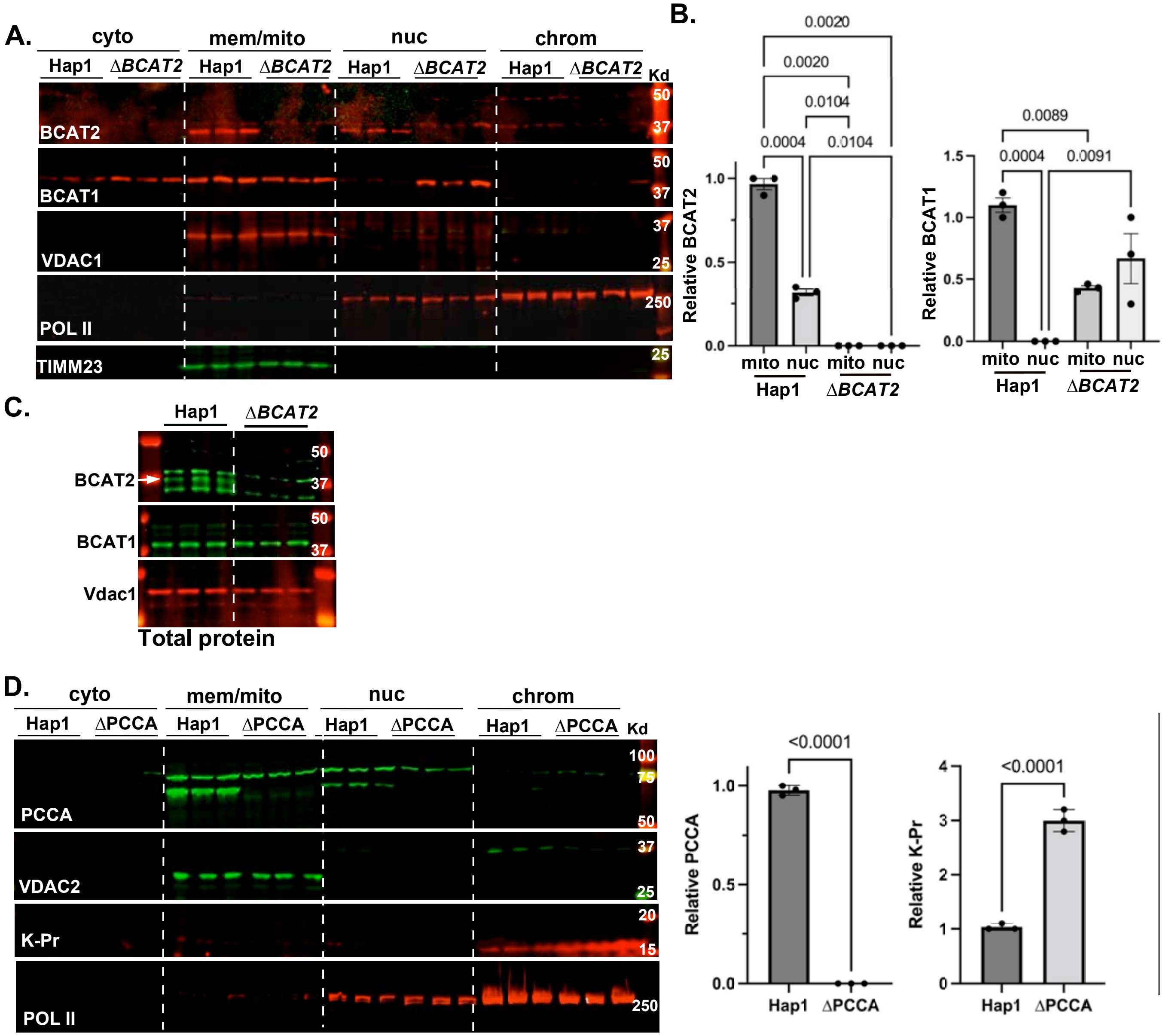

Figure 2S. Confirming BCAT2 and PCCA gene knockouts in Hap1 cells. A, C. Cultures of Hap1 and Hap1ΔBCAT2 cells, or **D.** Hap1 and Hap1ΔPCCA cells, were **A., D.** harvested, and fractionated into cytoplasm (cyto), membrane, which contains mitochondria (mem/mito), nucleoplasm (nuc), and chromatin (chrom) fractions, or **C.** collected and total protein extracted. The protein was analyzed by WB with the antibodies listed on the left in each panel. **B, E.** BCAT2, BCAT1**,** and PCCA in the mito and nuc fractions on the WB were quantitated, normalized to Vdac1 or Vdac2 and H3 (or pol II), respectively, and plotted as relative values after adjusting the signal of 'mito' samples to 1 (n=3). The data were analyzed by one-way Anova and the p values of those that are ≤ 0.05 are indicated on the graph (n=3, each)

The results confirm the absence of BCAT2 in the membrane/mitochondria and nucleoplasm fractions of the BCAT2 knockout cells, whereas BCAT1, which is not detected in the nucleoplasm in the parent Hap1 cells, translocates to the nucleus in Hap1ΔBCAT2, plausibly compensating for BCAT2's function, at least partially. This might explain the residual H3K23Pr signal observed in the presence of BCAA in these cells (main Fig. 1A).

Figure 2S

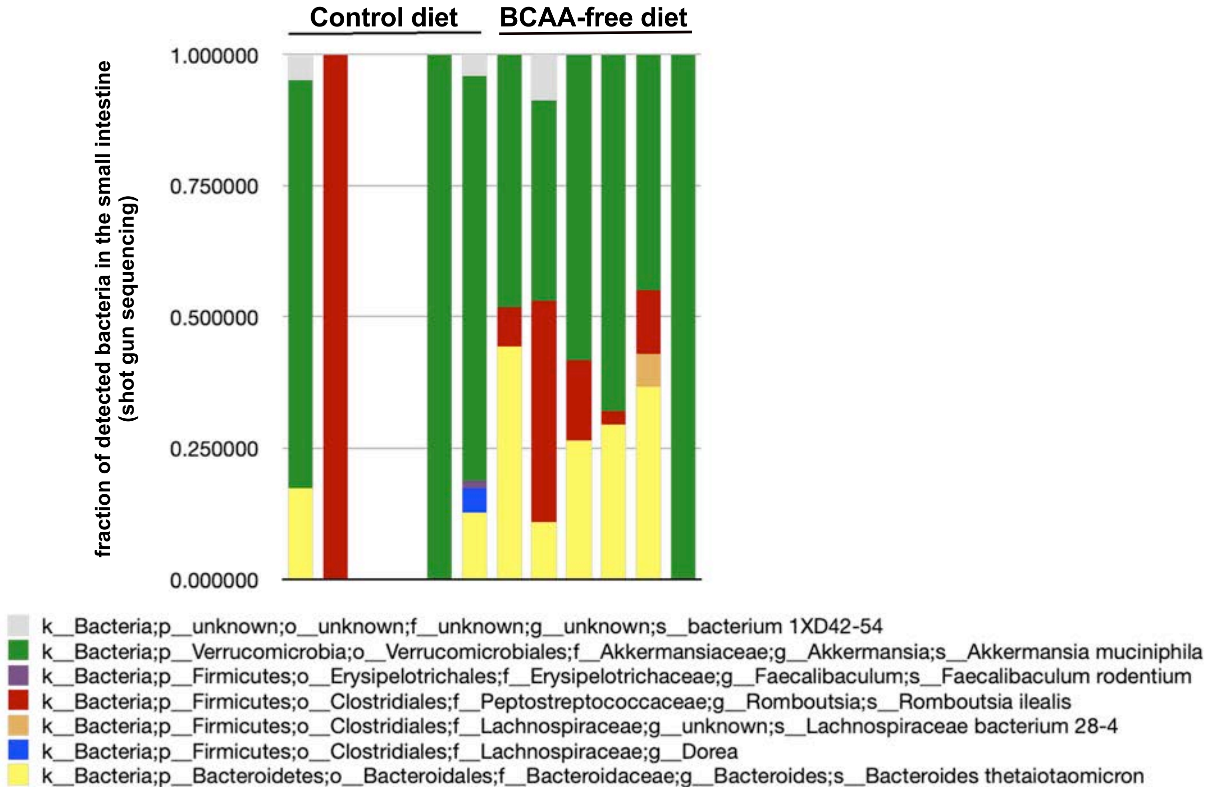

Figure 3S. Firmicutes and Bacteriodetes are more prevalent in mice guts on a BCAA-free diet. Mice were fed a BCAA-control or BCAA-free diet for 10 days. The small intestines were isolated and subjected to shotgun metagenomics. The results are presented in the stacked bar graph, showing the fractions of the bacterial families detected in each mouse. The color scheme for the family classification is listed below the graph.

Figure 3S

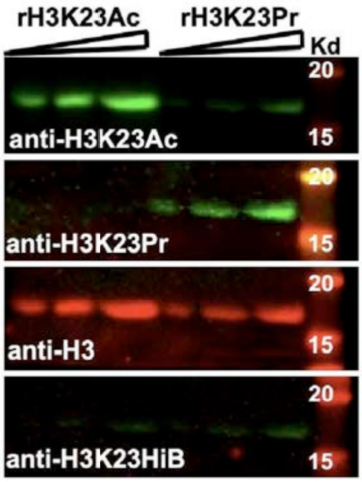

Figure 4S. Specificity of the H3K23-propionyl (H3K23Pr) antibody. Fifty, 100, and 200 nM of recombinant (r) nucleosomes harboring either rH3K23Ac or rH3K23Pr, as indicated on the top, were analyzed by Western blots for the antibodies listed on each panel.

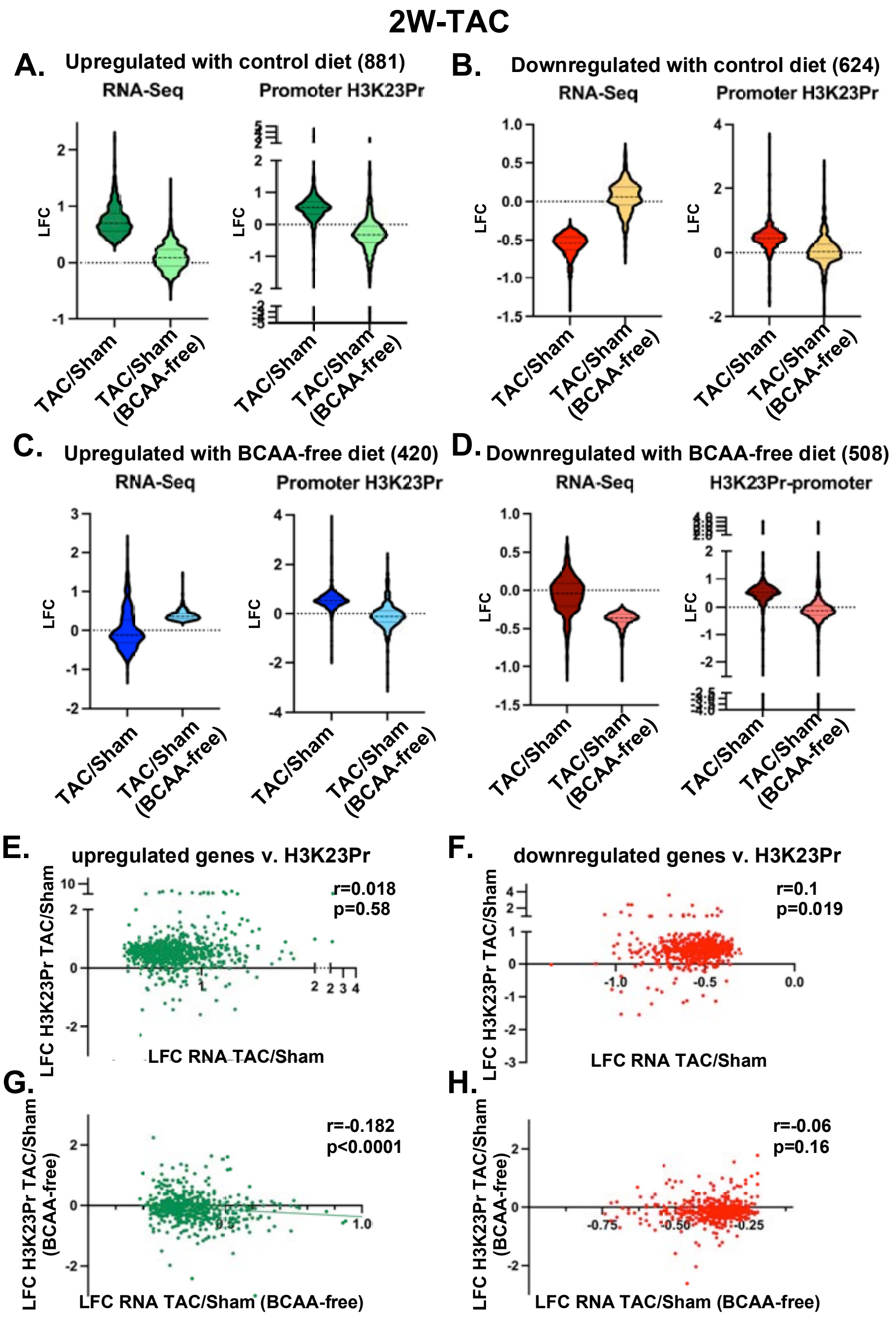

Figure 5S. The increase in promoter-H3K23Pr persists after 2W of pressure overload. H3K23Pr ChIP-Seq (-1000 to +1000) and RNA-Seq data from the 2W TAC experiment described in Fig. 3 (main text) were aligned by gene name and then sorted according to the genes' mRNA LFC TAC/sham that were significantly **A.** upregulated (padj ≤ 0.05) in the hearts of mice on a control diet, or **C.** on a BCAA-free diet, or **B.** downregulated (padj ≤ 0.05) in the hearts of mice on a control diet or **D.** on a BCAA-free diet. The log2 fold change (LFC) of RNA expression (RNA-Seq, left) and promoter-H3K23Pr (right) changes in the heart of TAC/Sham from these mice are presented as violin plots. **E-H.** Spearman's correlation analyses of LFC RNA expression v. LFC of promoter-H3K23Pr of TAC/Sham of significantly **E., G.** upregulated genes and **F., H.** downregulated genes. **G-J**. The RNA-Seq data from the 1W TAC on the BCAA-control and BCAA-free diets were analyzed by Gene Set Expression Analysis (GSEA). **G. I.** A complete list of the GO term enrichment. H., J. Examples of enrichment plots for some of the GO terms that are upregulated or downregulated during TAC.

Figure 5S

G.

GO gene set enrichment analysis (GSEA) - TCA v Sham, BCAA-control diet (alphabetical order)

enplot_GO_2_OXOGLUTARATE_METABOLIC_PROCESS_57.png
enplot_GO_AEROBIC_RESPIRATION_42.png
enplot_GO_APOPTOTIC_CELL_CLEARANCE_87.png
enplot_GO_BONE_REMODELING_84.png
enplot_GO_BONE_RESORPTION_78.png
enplot_GO_COLLAGEN_CONTAINING_EXTRACELLULAR_MATRIX_72.png
enplot_GO_COLLAGEN_FIBRIL_ORGANIZATION_66.png
enplot_GO_COLLAGEN_METABOLIC_PROCESS_120.png
enplot_GO_COLLAGEN_TRIMER_63.png
enplot_GO_COMPLEX_OF_COLLAGEN_TRIMERS_114.png
enplot_GO_CONDENSED_CHROMOSOME_CENTROMERIC_REGION_111.png
enplot_GO_ENDOPLASMIC_RETICULUM_LUMEN_93.png
enplot_GO_EXTRACELLULAR_MATRIX_90.png
enplot_GO_EXTRACELLULAR_MATRIX_STRUCTURAL_CONSTITUENT_69.png
enplot_GO_EXTRACELLULAR_MATRIX_STRUCTURAL_CONSTITUENT_CONFERRING_TENSILE
enplot_GO_EXTRACELLULAR_STRUCTURE_ORGANIZATION_75.png
enplot_GO_INNER_MITOCHONDRIAL_MEMBRANE_PROTEIN_COMPLEX_18.png
enplot_GO_MACROPHAGE_MIGRATION_96.png
enplot_GO_MITOCHONDRIAL_ELECTRON_TRANSPORT_NADH_TO_UBIQUINONE_54.png
enplot_GO_MITOCHONDRIAL_GENE_EXPRESSION_27.png
enplot_GO_MITOCHONDRIAL_MATRIX_51.png
enplot_GO_MITOCHONDRIAL_PROTEIN_COMPLEX_3.png
enplot_GO_MITOCHONDRIAL_RESPIRATORY_CHAIN_COMPLEX_ASSEMBLY_12.png
enplot_GO_MITOCHONDRIAL_TRANSLATION_9.png
enplot_GO_MITOCHONDRIAL_TRANSLATIONAL_TERMINATION_45.png
enplot_GO_MYELOID_LEUKOCYTE_MIGRATION_99.png
enplot_GO_NADH_DEHYDROGENASE_ACTIVITY_30.png
enplot_GO_NADH_DEHYDROGENASE_COMPLEX_24.png
enplot_GO_NADH_DEHYDROGENASE_COMPLEX_ASSEMBLY_15.png
enplot_GO_ORGANELLAR_RIBOSOME_60.png
enplot_GO_OXIDOREDUCTASE_ACTIVITY_ACTING_ON_NAD_P_H_QUINONE_OR_SIMILAR_C
enplot_GO_PHAGOCYTOSIS_105.png
enplot_GO_REGULATION_OF_BONE_REMODELING_102.png
enplot_GO_REGULATION_OF_LEUKOCYTE_MIGRATION_108.png
enplot_GO_RESPIRASOME_6.png
enplot_GO_RESPIRATORY_CHAIN_COMPLEX_21.png
enplot_GO_RESPIRATORY_ELECTRON_TRANSPORT_CHAIN_48.png
enplot_GO_TRANSLATIONAL_TERMINATION_33.png
enplot_GO_TRICARBOXYLIC_ACID_CYCLE_36.png
enplot_GO_VACUOLAR_LUMEN_117.png

Figure 5S-cont’

H, **Enrichment plots - TCA v Sham, BCAA-control diet**

Upregulated during TAC | **Downregulated during TAC**

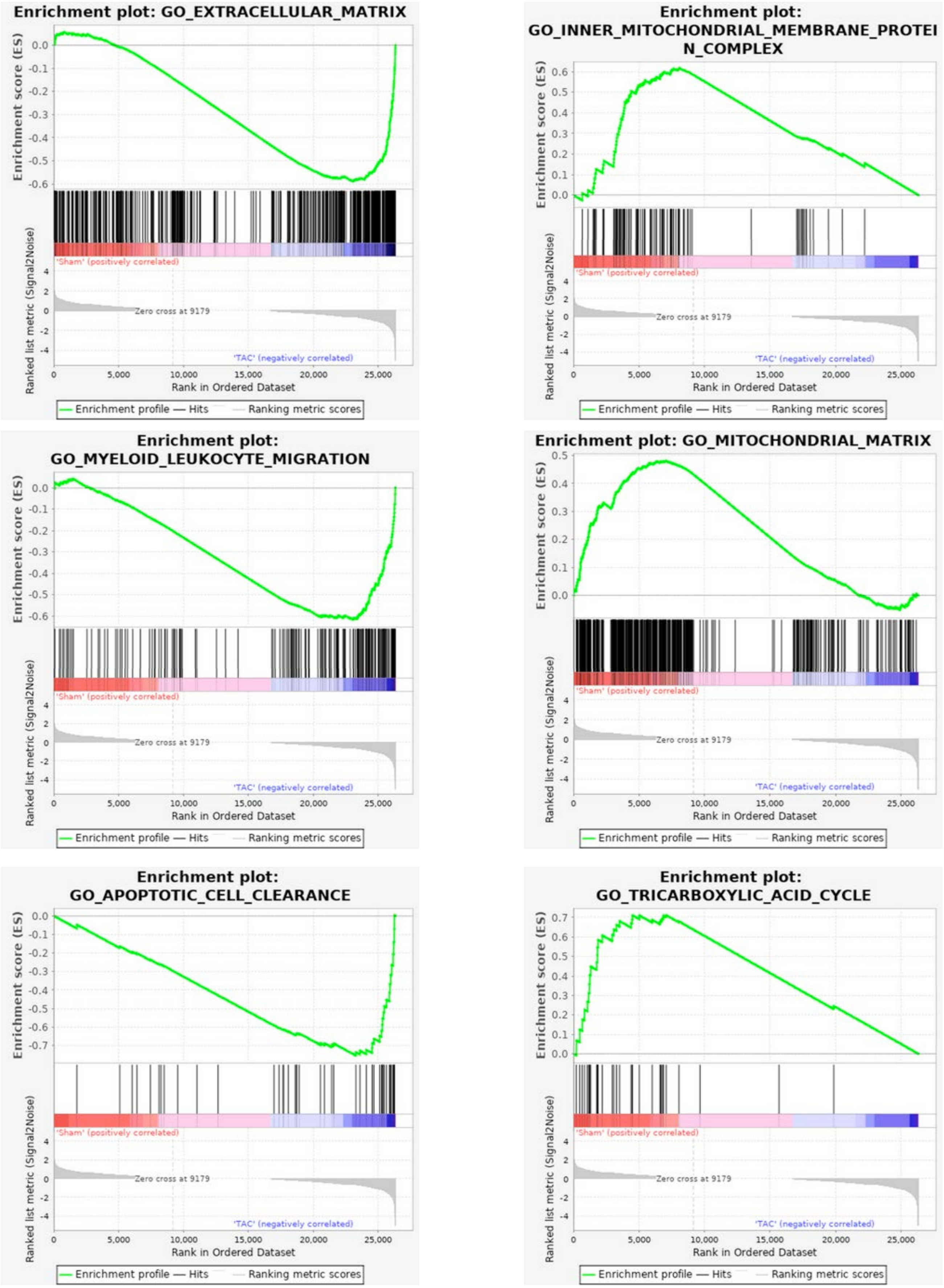

Figure 5S-cont'

I.

GO gene set enrichment analysis (GSEA) - TCA v Sham, BCAA-free diet (alphabetical order)

enplot_GO_ACTIN_FILAMENT_117.png
enplot_GO_ADRENERGIC_RECEPTOR_SIGNALING_PATHWAY_51.png
enplot_GO_APPENDAGE_MORPHOGENESIS_57.png
enplot_GO_CELL_PROLIFERATION_IN_FOREBRAIN_3.png
enplot_GO_COLLAGEN_BINDING_78.png
enplot_GO_COLLAGEN_CONTAINING_EXTRACELLULAR_MATRIX_72.png
enplot_GO_COLLAGEN_FIBRIL_ORGANIZATION_84.png
enplot_GO_COLLAGEN_TRIMER_69.png
enplot_GO_COLUMNAR_CUBOIDAL_EPITHELIAL_CELL_DEVELOPMENT_60.png
enplot_GO_COMPLEMENT_ACTIVATION_81.png
enplot_GO_DORSAL_SPINAL_CORD_DEVELOPMENT_6.png
enplot_GO_EMBRYONIC_CAMERA_TYPE_EYE_MORPHOGENESIS_18.png
enplot_GO_ENTEROENDOCRINE_CELL_DIFFERENTIATION_48.png
enplot_GO_EXTRACELLULAR_MATRIX_96.png
enplot_GO_EXTRACELLULAR_MATRIX_STRUCTURAL_CONSTITUENT_63.png
enplot_GO_EXTRACELLULAR_MATRIX_STRUCTURAL_CONSTITUENT_CONFERRING_TENSILE
enplot_GO_HISTONE_METHYLTRANSFERASE_COMPLEX_27.png
enplot_GO_INNER_MITOCHONDRIAL_MEMBRANE_PROTEIN_COMPLEX_108.png
enplot_GO_INTRINSIC_COMPONENT_OF_PRESYNAPTIC_MEMBRANE_42.png
enplot_GO_KILLING_OF_CELLS_OF_OTHER_ORGANISM_105.png
enplot_GO_MANNOSYLATION_30.png
enplot_GO_MITOCHONDRIAL_TRANSLATIONAL_TERMINATION_93.png
enplot_GO_MUSCLE_FILAMENT_SLIDING_114.png
enplot_GO_NEGATIVE_REGULATION_OF_CHOLESTEROL_EFFLUX_36.png
enplot_GO_NEURONAL_STEM_CELL_POPULATION_MAINTENANCE_12.png
enplot_GO_ORGANELLAR_RIBOSOME_90.png
enplot_GO_PLATELET_AGGREGATION_111.png
enplot_GO_POSITIVE_REGULATION_OF_NEUROTRANSMITTER_SECRETION_54.png
enplot_GO_POSITIVE_REGULATION_OF_VASCULAR_ENDOTHELIAL_CELL_PROLIFERATION_4
enplot_GO_REGULATION_OF_FATTY_ACID_BETA_OXIDATION_24.png
enplot_GO_REGULATION_OF_FEEDING_BEHAVIOR_15.png
enplot_GO_REGULATION_OF_INFLAMMATORY_RESPONSE_TO_ANTIGENIC_STIMULUS_21.
enplot_GO_REGULATION_OF_PEPTIDYL_SERINE_PHOSPHORYLATION_OF_STAT_PROTEIN_[illegible]
enplot_GO_REGULATION_OF_SYNAPTIC_TRANSMISSION_GABAERGIC_33.png
enplot_GO_RESPIRATORY_CHAIN_COMPLEX_120.png
enplot_GO_RIBOSOMAL_SUBUNIT_75.png
enplot_GO_SMALL_RIBOSOMAL_SUBUNIT_87.png
enplot_GO_STARTLE_RESPONSE_39.png
enplot_GO_STRUCTURAL_CONSTITUENT_OF_RIBOSOME_66.png
enplot_GO_VASCULAR_ENDOTHELIAL_CELL_PROLIFERATION_9.png

Figure 5S-cont’

J.

Enrichment plots - TCA v Sham, BCAA-free diet

Upregulated during TAC | **Downregulated during TAC**

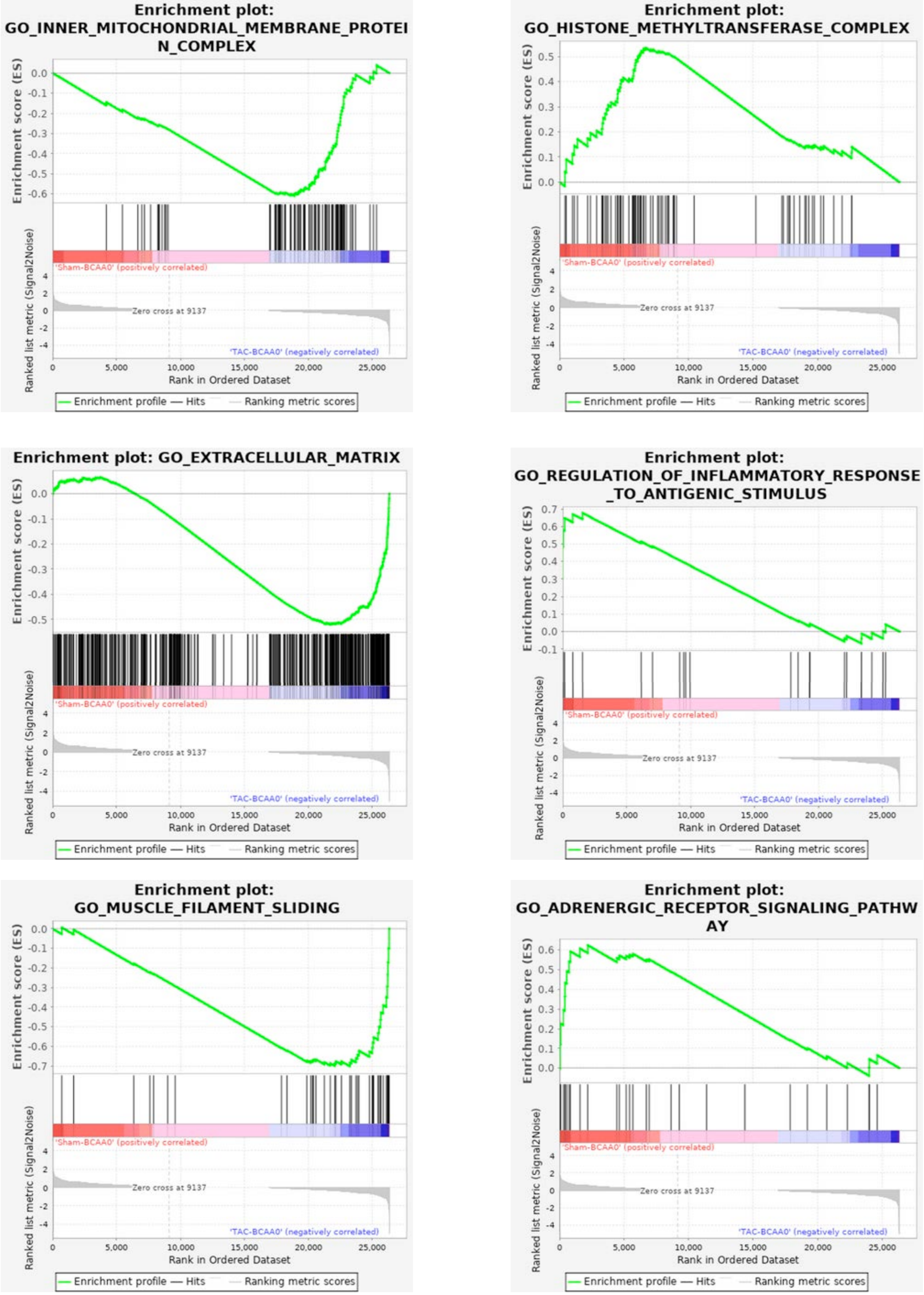

Figure 5S-cont'

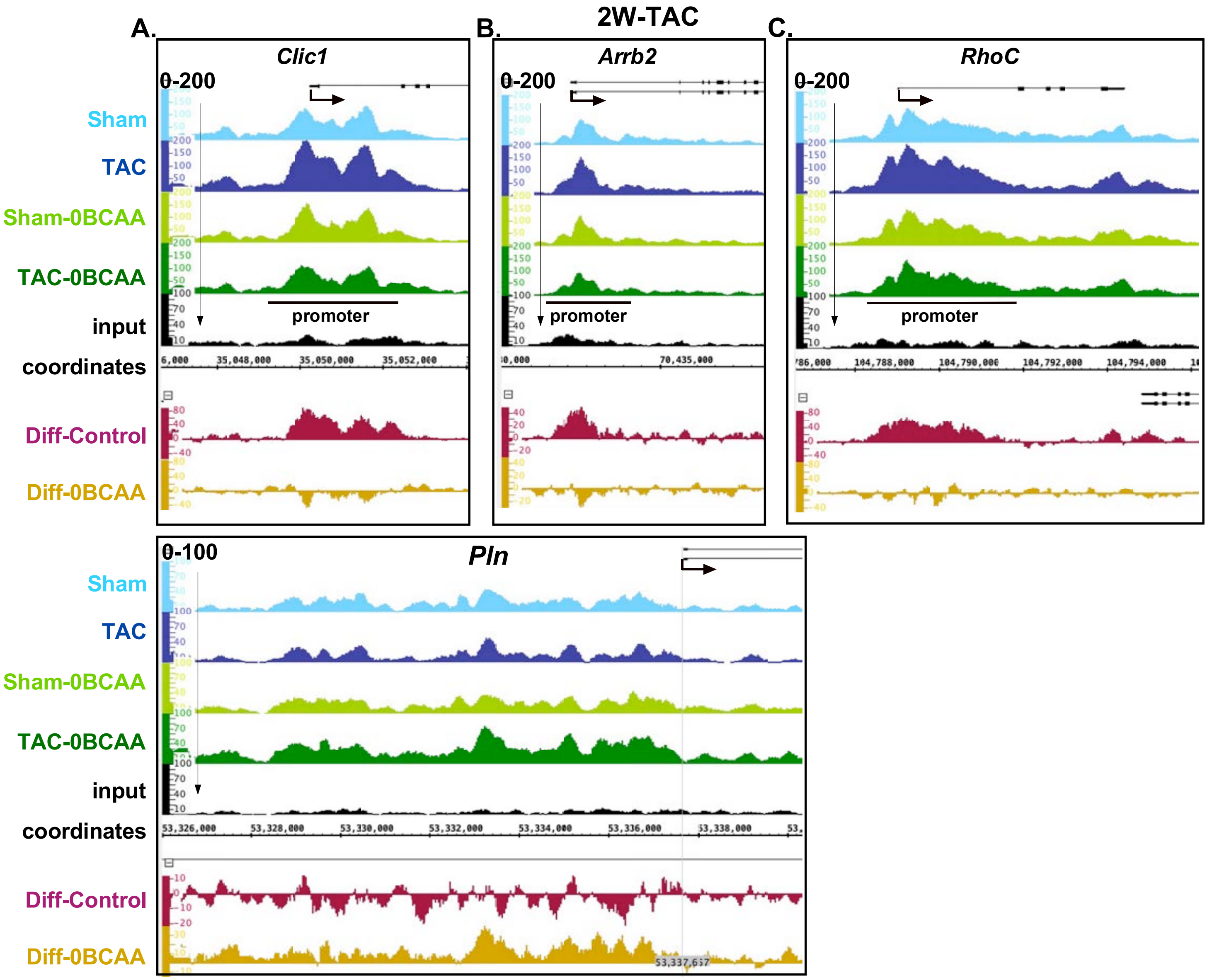

Figure 6S. TAC-induced upregulation of promoter-H3K23Pr is completely abrogated by a BCAA-free diet after 2W of pressure overload. Integrated genome browser images showing H3K23Pr ChIP-sequence tags from the 2W sham and TAC hearts of mice on the different diets, aligned across **A.** *Clic1*, **B.** *Arrb2*, **C.** *RhoC,* and **D.** *Pln*, gene coordinates. The labels on the left of each track indicate the surgical and diet conditions applied in mice; Sham and TAC surgeries with the BCAA-control diet (blue tracks), with the BCAA-free diets (green tracks); the differences (Diff) in the H3K23Pr sequence tags of TAC minus sham are shown in separate tracks for the control diet (Diff-Control, brown track) and BCAA-free diet (Diff-0BCAA, gold track).

Figure 6S

E.

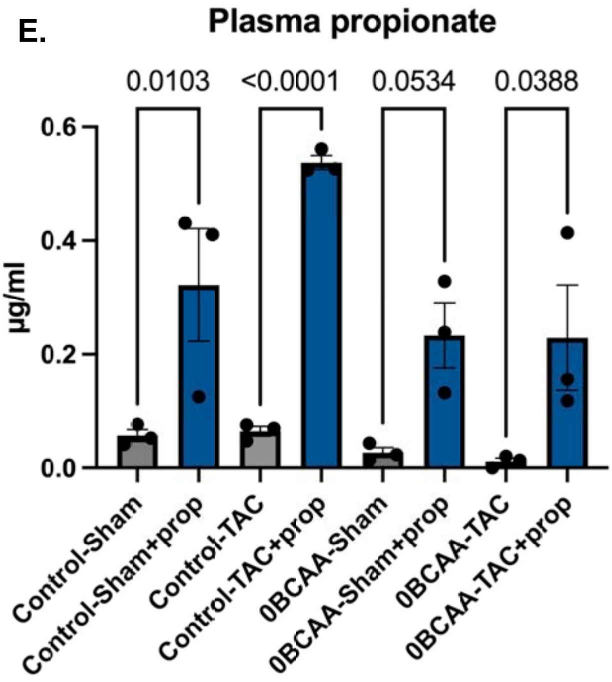

F.

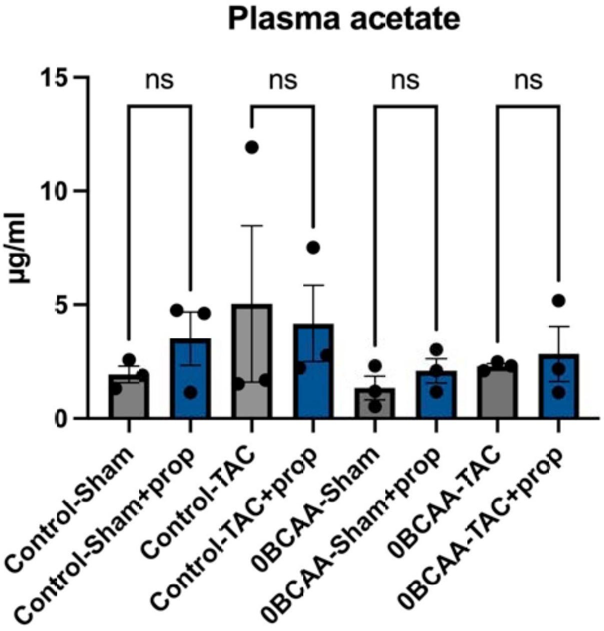

Figure 6S-E-F. Dietary propionate increases circulating propionate levels. Mice were fed a control or BCAA-free (0BCAA) diet without (grey bars) or with 1% dietary propionate (prop, blue bars) supplement for 4 days, before subjecting them to a sham or TAC operation. After 1 W the mice were analyzed with echocardiography, sacrificed, the plasma collected from the left ventricle, and the levels of short chain fatty acids measured (Creative Proteomics). Briefly, "samples were diluted in water containing labelled internal standards for each chain length (C2-C6). The free short chain fatty acids were derivatized using methyl chloroformate in 1-propanol yielding propyl esters before subsequent liquid-liquid extraction into hexane and analysis on a SLB-5ms (30 × 0.25 mm × 1.0 μm) column and detection using GC-EI-MS in SIM-mode. The analytes were quantified using 8-point calibration curves." Creative Proteomics, n=3, each. The results for plasma propionate (left) and acetate (right) concentrations (μg/ml) were plotted as bar graphs. Error bars represent S.E.M. The data were analyzed by one-way Anova using multiple pairwise comparisons of samples of those with vs. without propionate, for each of the conditions.

Figure 6S-cont'

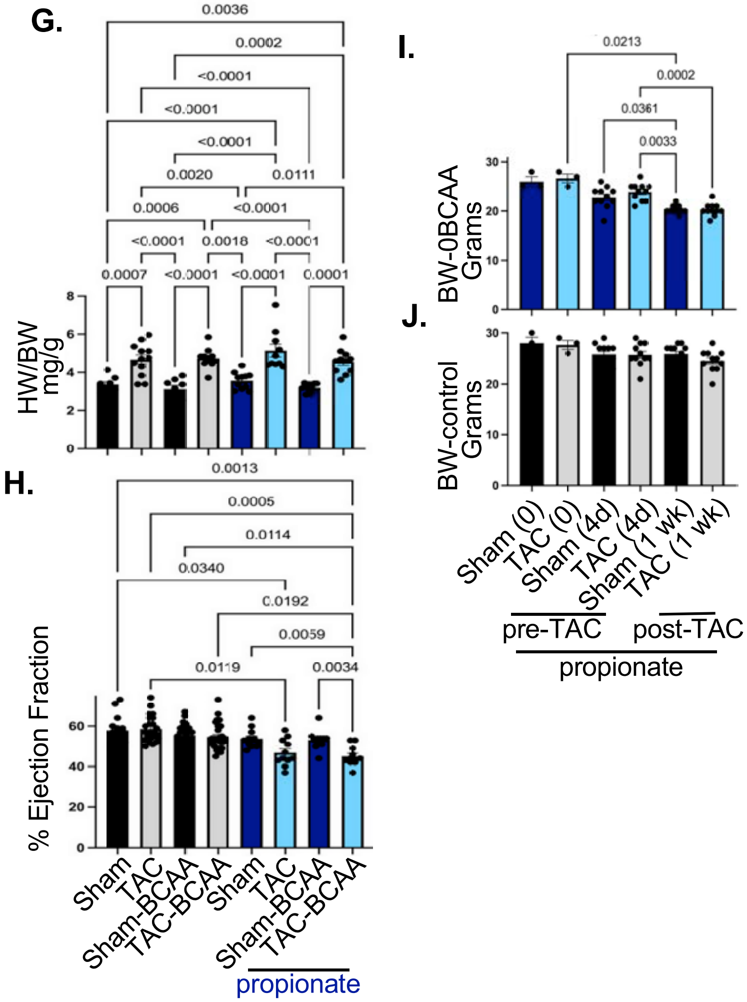

Figure 6S-G-J. Mice were fed a BCAA-control of BCAA-free diet without or with 1% propionate. After 4d they were subjected to a sham or TAC surgery. After 1W, mice were examined by echocardiography and the **G.** heart weight/body weight (HW/BW, mg/g) was calculated and graphed. **H.** Ejection fraction (EF) was graphed as %EF. **I-J**. BW (g) was measured and graphed. All results were analyzed by one-way Anova, and the p values of those that are ≤0.05 are listed above the brackets encompassing the bars.

Figure 6S (cont')

K.

TAC-induced upregulation of gene expression after addition of propionate to the BCAA-free diet

LFC	Gene	LFC	Gene	LFC	Gene	LFC	Gene
0.59	45174.00	0.40	Arhgef25	1.27	Ccr5	0.31	Cox6a2
0.57	1600002K03Rik	1.07	Arl5c	0.57	Cd14	0.93	Cpxm1
0.73	1700017B05Rik	0.50	Armcx2	1.15	Cd248	0.69	Creb3l1
0.81	1700020L24Rik	0.85	Arntl	0.93	Cd300c2	0.41	Crip1
0.72	1700120C14Rik	0.45	Arpc1b	1.30	Cd300lb	0.35	Crip2
1.08	2200002D01Rik	1.09	Arpp21	0.58	Cd33	1.81	Crmp1
1.14	2210407C18Rik	0.34	Arrb1	0.90	Cd52	0.47	Crtap
0.31	2310022B05Rik	0.68	Arrb2	0.71	Cd68	0.32	Csf1
0.42	3632451O06Rik	0.46	Arsa	1.61	Cd72	0.72	Csf1r
1.06	4632427E13Rik	0.37	Atg2a	1.03	Cd84	0.90	Csf2rb
1.76	5330406M23Rik	0.65	Atp1b2	0.39	Cd93	1.46	Csf2rb2
1.41	5430431A17Rik	0.36	Atp2a3	1.03	Cdc20	0.54	Csk
0.94	Abcc3	0.39	Atp9a	0.58	Cdc25b	0.66	Cspg4
0.41	Abcd1	0.38	Atxn7l3b	0.32	Cdc42ep1	0.46	Csrp1
0.52	Abhd14b	0.48	AU020206	0.58	Cdc42ep2	0.57	Csrp2
2.00	Acan	0.35	Aup1	0.45	Cdc42se1	0.40	Ctsa
0.31	Acot7	0.36	Auts2	0.36	Cdk2ap1	0.40	Ctsb
1.20	Acta2	0.35	Axl	0.93	Cdo1	0.54	Ctsk
1.05	Actb	0.74	B3galnt1	0.83	Cdr2l	0.85	Ctss
0.56	Actg1	0.92	B4galnt1	0.78	Cebpa	0.53	Ctsz
1.45	Actg2	0.34	B4galt3	0.30	Celf2	0.40	Cx3cl1
0.36	Actn1	0.44	B4galt5	0.34	Cep170b	1.30	Cx3cr1
0.44	Acvrl1	0.56	Bbc3	1.14	Cercam	0.35	Cxcl12
1.34	Adam12	0.53	BC017158	1.63	Cfd	0.70	Cxcl16
0.35	Adam15	0.47	Bcar3	0.44	Cfl1	1.49	Cxcr4
0.54	Adam19	0.52	Bcl2l11	0.48	Cfp	0.46	Cyb5r3
0.65	Adam33	0.46	Bcl2l12	1.03	Chad	0.40	Cyba
1.21	Adam8	0.98	Bcl3	0.31	Chd3	0.71	Cybb
0.62	Adamts15	0.43	Bcl6	0.37	Chpf	0.31	Cyp27a1
1.93	Adamts4	0.43	Bcr	0.33	Chpf2	0.93	Cyp2e1
0.55	Adamts5	0.89	Bgn	0.60	Chst1	0.39	Cyp2u1
0.67	Adamtsl1	0.53	Bin1	0.82	Chst12	1.16	Cyp4f18
0.53	Adcy4	1.09	Blnk	0.41	Cisd3	0.98	Cyth4
0.59	Adcyap1r1	0.42	Blvrb	0.55	Cited4	0.32	D17H6S53E
0.87	Adgrd1	0.37	Bmp1	0.49	Ckap4	0.44	Dab2
0.74	Adgre1	0.49	Brf2	0.47	Ckb	1.26	Dact2
0.64	Adgrg3	0.54	Bscl2	0.77	Clec10a	0.44	Dap
0.34	Adgrl1	1.04	Btg2	0.85	Clec12a	0.80	Dbh
1.46	Adipoq	0.37	Bud13	0.38	Clec3b	0.50	Dcakd
0.50	Adora2a	1.43	C130023A14Rik	1.18	Clec4a1	0.37	Deaf1
0.79	Aebp1	0.87	C1qa	1.22	Clec4d	0.31	Dennd1a
0.45	Agpat1	0.80	C1qb	0.98	Clec4n	0.46	Des
0.70	Agpat4	0.60	C1qc	0.37	Clic1	0.31	Dgcr2
0.68	Agt	0.59	C1qtnf2	0.46	Clip2	0.80	Dhcr24
0.37	Ahdc1	1.66	C1qtnf3	0.68	Clip3	0.45	Dhrs3
0.85	Ahnak2	1.07	C1qtnf6	0.70	Clmp	0.33	Dhx8
0.42	AI480526	0.40	C1ra	0.49	Cln6	0.58	Dlg4
0.40	AI506816	0.34	C1s1	1.41	Clu	0.51	Dll4
1.23	Aif1	0.77	C3	1.62	Cmtm5	0.49	Dnajb1
0.47	Ajuba	1.38	C3ar1	0.31	Cmtm6	0.39	Dnm1
1.45	Aldh1a2	0.93	C4b	1.21	Cnn1	0.81	Dock2
0.46	Alg2	0.92	C5ar1	0.37	Cnn2	0.52	Dpagt1
0.42	Alox5ap	1.02	Cacnb3	0.35	Cnot9	1.30	Dpep2
1.05	Alox8	0.72	Cadm3	0.31	Cnppd1	0.50	Dph2
0.38	Alpk2	0.30	Cald1	0.44	Coa4	0.42	Dpt
0.46	Alpl	0.32	Calr	1.07	Col18a1	0.77	Dpysl2
0.46	Anpep	0.30	Cap1	2.21	Col1a1	0.51	Dtwd1
0.40	Anxa2	0.44	Capg	1.77	Col1a2	0.90	Dtx4
0.60	Aoc3	0.34	Capn15	1.91	Col3a1	0.50	Dusp10
1.04	Aph1c	0.70	Car11	0.88	Col4a6	0.41	Dusp23
0.49	Apod	1.67	Car3	1.08	Col5a1	0.69	Dusp5
0.76	Apoe	0.49	Cavin1	0.94	Col5a3	0.45	Dusp6
0.69	Aqp7	0.41	Cavin3	1.12	Col6a1	0.36	Dxo
0.31	Arfgap1	0.54	Cbr2	1.21	Col6a2	0.48	Dynll1
0.62	Arhgap19	0.59	Cbx4	1.21	Col6a3	0.46	Ebf1
0.58	Arhgap30	0.52	Cbx6	0.61	Col6a6	0.81	Ecm1
0.72	Arhgap45	0.52	Ccdc134	2.42	Col9a2	0.32	Eef2kmt
0.42	Arhgdia	0.49	Ccdc191	0.71	Comtd1	0.52	Efemp1
0.41	Arhgdib	0.68	Ccdc3	0.52	Coro1a	0.81	Efemp2
0.41	Arhgef15	1.88	Ccl8	0.47	Coro1c	0.98	Efhd1
0.32	Arhgef2	1.10	Ccr2	0.65	Cotl1	0.86	Efna5

LFC	Gene	LFC	Gene	LFC	Gene	LFC	Gene
1.21	Efs	0.38	Gal3st3	0.60	Htra3	0.54	Litaf
0.93	Egfl8	0.62	Galns	0.33	Ier2	0.57	Lmnb1
0.55	Egfr	0.58	Galnt16	0.31	Ier3	0.61	Lmod1
0.85	Egr1	0.47	Galnt17	0.33	Ier5	0.57	Loxl1
1.05	Egr2	1.30	Galnt6	0.87	Ier5l	0.58	Lpar1
0.56	Ehd2	0.94	Gas7	0.51	Iffo2	0.31	Lrg1
0.33	Elk3	0.41	Gatsl2	0.40	Ifi27	0.48	Lrp1
1.60	Eln	0.83	Gdf11	0.39	Ifitm2	0.37	Lrp10
0.32	Elof1	1.41	Gfra3	0.60	Ifitm3	0.34	Lrrc14
0.52	Elovl1	0.32	Gga3	0.30	Ifnar2	0.72	Lrrc17
0.47	Emp3	0.67	Ggcx	0.46	Ift46	1.22	Lrrc25
0.39	Eng	0.49	Ggt5	0.95	Igf1	0.66	Lrrc32
0.56	Engase	0.49	Gimap1	0.67	Igf2	0.74	Lrrc75a
0.48	Entpd2	0.46	Gja1	0.69	Igf2bp2	0.59	Lrrn2
1.03	Ephb6	0.91	Gja5	0.74	Igfbp4	0.60	Lsp1
0.75	Eps8l2	0.34	Glb1	0.58	Igfbp5	0.52	Ltbp3
0.32	Ergic1	0.70	Gli1	0.64	Igsf3	0.66	Ltc4s
0.32	Esyt1	0.80	Glipr2	0.44	Igsf8	0.43	Ly6a
0.42	Ets1	0.62	Glis2	1.10	Igsf9	0.56	Ly6e
0.48	Eva1b	0.40	Glmp	0.71	Ikbke	0.70	Lyl1
0.34	Evc	0.38	Gltp	0.90	Il10ra	0.52	Lyz2
1.14	Evi2a	0.59	Gm47283	0.69	Il16	0.62	Maf
0.34	Ext1	0.61	Gmfg	0.58	Il17ra	0.33	Maged2
0.63	Faap20	0.55	Gmip	0.52	Il3ra	0.81	Map1a
0.30	Fads1	0.52	Gngt2	0.32	Il4ra	0.36	Map1s
0.78	Fads2	0.79	Gpc3	0.63	Il6ra	0.53	Map3k14
0.53	Fam102a	2.03	Gpr132	0.50	Inpp5d	1.23	Map3k7cl
1.33	Fam105a	0.31	Gpr137	0.37	Inpp5j	0.43	Map6
0.37	Fam129b	0.47	Gpr153	0.43	Ints5	1.08	Marcks
0.88	Fam181b	1.66	Gpr183	0.38	Irf3	0.31	Marveld1
0.35	Fam193a	0.78	Gpr19	0.82	Irf8	1.60	Matn4
2.68	Fam196a	0.58	Gpr4	0.42	Irgm2	0.38	Mcam
0.72	Fam206a	1.20	Gpr65	0.62	Itga8	0.50	Mcm3
0.46	Fam210b	0.61	Gprc5c	0.84	Itgam	0.34	Mcrs1
0.48	Fam222b	0.72	Gpsm3	0.50	Itgb2	0.89	Meg3
0.45	Fam43a	0.50	Gpx1	0.31	Itgb5	0.37	Metrnl
1.19	Fam46c	0.57	Gpx7	1.87	Itih4	0.33	Mettl1
0.36	Fam53b	0.31	Grhpr	0.44	Itpripl1	0.43	Mettl26
1.52	Fasn	0.52	Grik5	0.43	Jag1	1.22	Mfap2
0.70	Fastkd5	0.33	Grina	0.39	Jmjd8	0.45	Mfge8
0.51	Fbln2	0.59	Grn	0.51	Jun	0.57	Mfsd10
0.44	Fbln5	0.63	Grrp1	0.48	Junb	0.47	Mfsd12
1.37	Fbln7	1.39	Gspt2	0.31	Jund	0.51	Mhrt
1.14	Fbn2	0.50	Gstm1	0.34	Kat2a	0.37	Micall1
0.44	Fbxl22	0.42	Gstm2	0.64	Kcna2	0.41	Miip
0.47	Fbxo10	0.43	Gtf3c5	0.87	Kcnmb1	0.73	Mir133a-2
0.76	Fbxo46	0.31	Gucy1a1	0.72	Kcnq1ot1	0.52	Mir208a
1.16	Fcer1g	0.49	Gusb	1.05	Kcp	0.67	Mir208b
0.56	Fcgr2b	0.32	H13	1.00	Kctd11	0.36	Mir99ahg
0.82	Fcgr3	0.93	H19	0.42	Kctd17	0.56	Mki67
1.50	Fcgr4	0.56	H2-Aa	0.73	Kctd21	0.49	Mkl1
0.34	Fcgrt	0.44	H2afx	0.76	Kctd7	0.43	Mmd
0.87	Fcna	0.35	H6pd	0.57	Kdelr2	0.41	Mmp11
0.36	Fdps	0.47	Hdac10	0.98	Kdelr3	3.10	Mmp12
0.74	Fermt3	0.44	Hes1	1.42	Kif23	1.03	Mmp14
0.67	Fgd2	0.81	Hey2	1.18	Klf2	0.56	Mmp23
1.18	Fgd3	0.79	Heyl	0.59	Klhl25	0.46	Mmrn1
0.32	Fli1	0.33	Hhatl	0.62	Kmt5c	0.50	Morn2
1.13	Fn1	0.62	Hid1	0.68	L1cam	0.36	Mospd3
0.69	Fndc10	0.77	Hivep3	1.04	Laptm5	0.37	Mpdu1
1.57	Fos	0.31	Hjurp	0.61	Lasp1	0.54	Mpeg1
0.90	Foxc2	0.92	Hlx	0.52	Lbp	0.56	Mrc2
0.47	Foxo1	0.40	Hmox1	1.36	Lcat	1.38	Mrgprf
0.69	Foxo6	1.07	Hoxb3os	0.82	Lcn2	1.61	Ms4a7
1.16	Foxs1	1.34	Hp	0.61	Lcp1	1.02	Msc
0.36	Fpgs	1.21	Hpgds	0.80	Lgals3	0.63	mt-Nd3
0.66	Ftl1	0.49	Hr	0.69	Lgals3bp	1.11	mt-Tg
0.58	Fxyd5	0.43	Hs1bp3	0.65	Lgi4	0.57	mt-Th
0.65	Fzd2	0.70	Hs3st1	0.43	Lgmn	0.60	mt-Tl2
0.52	G0s2	1.25	Hsp25-ps1	0.37	Lhpp	0.38	mt-Ts2
0.53	G6pdx	0.74	Hspa2	0.50	Limd2	0.52	mt-Tt

LFC	Gene	LFC	Gene	LFC	Gene	LFC	Gene
0.41	Mt1	0.52	mt-Tt	0.79	Pcdhb22	2.19	Ptprv
0.58	Mt2	0.41	Mt1	0.94	Pced1b	1.30	Pycr1
0.33	Mthfr	0.58	Mt2	0.42	Pcgf1	0.60	Pygo2
0.61	Lmod1	0.33	Mthfr	0.84	Pcolce	0.50	Rab13
0.97	Loxl1	0.72	Muc6	0.81	Pcyox1l	0.40	Rab23
0.58	Lpar1	0.70	Mustn1	0.32	Pde4d	0.40	Rab34
0.31	Lrg1	0.34	Myadm	0.67	Pdgfa	0.40	Rab3il1
0.48	Lrp1	0.83	Mybpc2	0.56	Pdgfb	1.20	Rac2
0.37	Lrp10	0.44	Myct1	0.46	Pdgfrb	0.60	Rap1gap
0.34	Lrrc14	0.49	Myd88	0.59	Pdia5	0.80	Rap2b
0.72	Lrrc17	1.14	Myh11	0.41	Pdlim3	0.40	Rapsn
1.22	Lrrc25	0.43	Myh9	0.63	Pdlim7	0.60	Rara
0.66	Lrrc32	0.41	Myl12b	0.69	Pdpn	0.30	Rarg
0.74	Lrrc75a	0.36	Myl6	0.53	Pf4	0.60	Rasl12
0.59	Lrrn2	0.87	Myl9	0.44	Pgghg	0.60	Rbm12
0.60	Lsp1	0.36	Mylk	0.31	Pgls	0.50	Rbms3
0.52	Ltbp3	0.44	Myo1d	0.32	Phc2	0.60	Rcc2
0.66	Ltc4s	0.61	Myo1e	0.68	Phkg1	0.80	Rcn3
0.43	Ly6a	0.81	Myo1f	1.19	Pianp	0.40	Recql5
0.56	Ly6e	1.38	Mypopos	0.31	Pias3	0.50	Rela
0.70	Lyl1	0.42	NA	0.85	Pik3cd	0.60	Rem1
0.52	Lyz2	0.40	NA	0.93	Pirb	0.40	Rest
0.62	Maf	0.32	Naga	0.59	Pirt	2.40	Retn
0.33	Maged2	0.62	Nat14	0.30	Pitpnm1	0.40	Retreg2
0.81	Map1a	0.62	Nbl1	0.52	Pknox2	0.30	Retreg3
0.36	Map1s	0.92	Ncam1	0.75	Pla2g15	0.50	Rflnb
0.53	Map3k14	0.44	Ncdn	0.43	Pla2g6	0.40	Rfx1
1.23	Map3k7cl	0.59	Ncf1	0.65	Pla2g7	0.30	Rfxank
0.43	Map6	0.56	Ncf2	1.53	Platr32	0.30	Rgl1
1.08	Marcks	1.21	Ncf4	0.43	Plcl2	0.40	Rgma
0.31	Marveld1	0.62	Nckap1l	0.48	Pld3	0.90	Rgs10
1.60	Matn4	0.49	Nckap5l	0.85	Pld4	1.50	Rgs11
0.38	Mcam	0.48	Nckipsd	0.48	Plekhg3	0.50	Rgs4
0.50	Mcm3	0.60	Ndn	0.49	Plekhh3	0.30	Rhoc
0.34	Mcrs1	0.63	Ndufa4l2	0.79	Plekho2	0.70	Rhog
0.89	Meg3	0.30	Ndufb7	0.46	Plod3	0.40	Rhoj
0.37	Metrnl	0.45	Neat1	0.42	Plscr3	0.70	Rhou
0.33	Mettl1	0.72	Nes	0.71	Pltp	0.50	Rian
0.43	Mettl26	0.35	Neurl2	0.59	Pml	0.30	Rin2
1.22	Mfap2	0.80	Neurl3	1.63	Pnpla3	0.90	Ripor3
0.45	Mfge8	0.83	Nfatc4	0.33	Pnpla7	0.40	Rnaseh2c
0.57	Mfsd10	0.40	Nfib	0.32	Podxl	0.50	Rnd2
0.47	Mfsd12	0.47	Nfil3	0.34	Poldip3	0.40	Rnf157
0.51	Mhrt	0.46	Nos2	0.47	Polr2a	0.90	Rnf26
0.37	Micall1	0.35	Nos3	0.38	Pomt1	0.30	Rnpep
0.41	Miip	0.45	Notch3	2.03	Pou2f2	0.40	Rpl24
0.73	Mir133a-2	0.32	Npc2	0.53	Ppan	0.30	Rpl28
0.52	Mir208a	0.44	Npdc1	0.38	Ppic	0.30	Rpl41
0.67	Mir208b	0.34	Npr1	0.47	Ppm1m	0.30	Rps28
0.36	Mir99ahg	0.88	Nptxr	0.64	Ppp1r10	0.30	Rps29
0.56	Mki67	1.69	Nr5a2	0.47	Ppp1r14a	0.80	Rps6ka1
0.49	Mkl1	0.52	Nrep	0.36	Pprc1	0.30	Rps6kb2
0.43	Mmd	0.49	Nrros	0.76	Praf2	0.60	Rrad
0.41	Mmp11	0.37	Nt5dc2	0.47	Prag1	0.40	Rras
3.10	Mmp12	1.73	Ntrk2	1.53	Prc1	0.40	Rrnad1
1.03	Mmp14	0.44	Numbl	0.35	Prelid1	1.20	Rsph9
0.56	Mmp23	0.41	Nup214	0.56	Prelp	0.40	Rtl8b
0.46	Mmrn1	0.32	Nup62	0.47	Prex1	0.40	Rtn4rl1
0.50	Morn2	0.60	Oaf	0.35	Prickle1	0.90	Rtp3
0.36	Mospd3	0.89	Olfm1	1.08	Prkcb	0.40	Rusc1
0.37	Mpdu1	0.44	Olfml3	0.51	Prkcq	0.80	S100a11
0.94	Mpeg1	0.86	Opn4	0.58	Prr13	0.50	S100a13
0.96	Mrc2	0.45	Orai1	0.42	Prr33	0.60	S100a4
1.38	Mrgprf	0.46	Osbpl7	0.71	Prrt1	0.50	S100a6
1.61	Ms4a7	0.73	Osr1	1.30	Psd4	1.10	S1pr2
1.02	Msc	0.72	P2ry6	0.71	Pstpip1	0.70	S1pr3
0.63	mt-Nd3	1.58	P4ha3	1.33	Ptafr	0.40	Samd14
1.11	mt-Tg	0.35	Pabpc1	1.41	Ptch2	1.32	Pycr1
0.57	mt-Th	1.22	Pacsin1	0.52	Ptgis	0.64	Pygo2
0.60	mt-Tl2	0.32	Paqr4	0.47	Pth1r	0.50	Rab13
0.38	mt-Ts2	0.34	Pcdh1	0.41	Ptms	0.43	Rab23

LFC	Gene	LFC	Gene	LFC	Gene	LFC	Gene
0.39	Rab34	0.70	Sdc1	1.57	Srarp	0.55	Tppp3
0.40	Rab3il1	0.57	Selenon	0.35	Srebf2	1.14	Tpsb2
1.19	Rac2	0.41	Selenow	0.81	Srpx	0.65	Traf4
0.58	Rap1gap	1.13	Selplg	0.64	Srpx2	1.24	Trem2
0.82	Rap2b	0.37	Sema3g	0.51	Srrm4	0.72	Trf
0.37	Rapsn	0.71	Sema6c	0.37	Srxn1	0.57	Tril
0.58	Rara	1.46	Serinc2	1.01	Ssc5d	0.39	Trim26
0.32	Rarg	1.91	Serpina3i	0.66	Ssh1	0.42	Trim47
0.60	Rasl12	1.45	Serpina3n	0.52	St3gal1	0.50	Trp53
0.64	Rbm12	2.39	Serpinb1a	0.48	St5	0.54	Tshz2
0.49	Rbms3	0.42	Serpine2	0.49	Stab1	1.31	Tsku
0.60	Rcc2	0.56	Serping1	0.80	Star	0.39	Tspyl4
0.79	Rcn3	0.55	Sesn2	0.62	Stmn2	0.41	Tssc4
0.41	Recql5	0.36	Setd1b	0.55	Stxbp2	1.60	Ttc25
0.48	Rela	0.42	Sf3a2	0.39	Supt7l	0.41	Ttyh3
0.58	Rem1	1.28	Sfn	1.20	Susd2	0.86	Tubb2b
0.39	Rest	0.53	Sfxn3	0.70	Susd5	1.36	Tubb3
2.37	Retn	0.60	Sgsh	0.40	Swap70	1.18	Tubb4a
0.37	Retreg2	1.03	Sh2b2	0.41	Syde1	0.67	Tusc5
0.30	Retreg3	0.98	Sh3bgrl3	0.35	Sys1	0.37	Tut1
0.50	Rflnb	0.58	Sh3bp1	0.44	Syt11	0.36	Txndc12
0.42	Rfx1	0.90	Sh3pxd2b	0.37	Taf3	0.78	Tyms
0.34	Rfxank	0.30	Sharpin	0.71	Tagln	0.54	Tyrobp
0.31	Rgl1	0.79	Shisa4	0.47	Tagln2	0.43	Ube2l6
0.44	Rgma	0.73	Shisa5	0.31	Taldo1	0.95	Ubxn10
0.90	Rgs10	0.36	Sipa1	0.74	Tapbp	0.50	Unc119b
1.52	Rgs11	0.47	Sirpa	0.42	Tbc1d10b	0.56	Unc45a
0.51	Rgs4	0.70	Six1	0.42	Tbc1d5	0.73	Unc93b1
0.29	Rhoc	0.41	Six5	0.35	Tbl2	0.80	Usp43
0.74	Rhog	0.63	Slc16a3	0.31	Tbx5	0.64	Vasn
0.36	Rhoj	0.78	Slc16a9	0.34	Tcf3	0.33	Vasp
0.69	Rhou	1.06	Slc1a3	0.50	Tcirg1	0.58	Vat1
0.52	Rian	0.91	Slc25a1	0.33	Tcn2	0.33	Vegfb
0.30	Rin2	0.42	Slc25a23	0.52	Tfe3	0.69	Vim
0.94	Ripor3	0.65	Slc25a45	0.44	Tgfb1	0.49	Vkorc1
0.36	Rnaseh2c	0.30	Slc27a4	0.55	Tgfb1i1	0.45	Vmac
0.54	Rnd2	1.19	Slc2a10	0.35	Tgfbr2	0.48	Vps18
0.43	Rnf157	0.47	Slc52a2	0.79	Tgif1	0.55	Vtn
0.88	Rnf26	0.49	Slc8b1	0.49	Thbs3	0.82	Was
0.35	Rnpep	0.59	Slc9a2	0.82	Thy1	0.74	Wfdc17
0.36	Rpl24	0.77	Slc9a9	1.49	Tifab	0.58	Wif1
0.30	Rpl28	0.36	Slco2b1	1.81	Timp1	0.38	Wipf2
0.34	Rpl41	0.77	Slfn2	0.35	Timp2	0.38	Wiz
0.33	Rps28	0.47	Slit3	0.66	Tkt	0.47	Wnt9a
0.30	Rps29	0.42	Smarcb1	0.33	Tle3	0.43	Wtip
0.79	Rps6ka1	0.37	Smarcd2	0.34	Tmed1	0.51	Xirp1
0.31	Rps6kb2	0.54	Smim3	0.34	Tmed3	0.42	Xylt2
0.56	Rrad	1.32	Snai1	0.75	Tmem119	0.36	Ybx2
0.38	Rras	0.53	Sncg	0.67	Tmem138	0.32	Yipf3
0.35	Rrnad1	0.36	Snhg20	0.72	Tmem173	0.54	Zbtb45
1.15	Rsph9	1.01	Snx20	0.62	Tmem176a	0.59	Zbtb8a
0.40	Rtl8b	0.52	Snx33	0.66	Tmem176b	0.48	Zc3h3
0.44	Rtn4rl1	0.59	Socs3	0.41	Tmem184b	0.32	Zdhhc1
0.94	Rtp3	0.79	Sod3	0.35	Tmem246	0.38	Zdhhc18
0.40	Rusc1	0.32	Sorbs3	0.45	Tmem41a	0.52	Zfhx3
0.81	S100a11	1.21	Sox10	0.73	Tmem51	0.41	Zfp142
0.49	S100a13	0.43	Sox13	0.33	Tmem63a	0.33	Zfp219
0.62	S100a4	0.30	Sox18	1.28	Tmie	0.45	Zfp36
0.50	S100a6	0.41	Sox4	2.14	Tnc	0.37	Zfp36l1
1.12	S1pr2	1.91	Sox9	1.40	Tnfaip8l2	0.60	Zfp385a
0.72	S1pr3	0.49	Sp110	0.68	Tnfrsf19	0.64	Zfp385b
0.43	Samd14	1.08	Sparc	0.40	Tnfrsf1a	0.47	Zfp395
0.33	Samd4b	0.53	Sphk2	0.87	Tnfrsf1b	0.41	Zfp414
1.51	Sap25	0.57	Spi1	0.44	Tnks1bp1	1.24	Zfp618
0.34	Sars2	0.34	Spns2	2.53	Tnnt3	0.45	Zfp703
0.52	Sbk2	1.82	Spon2	0.40	Tnxb	0.70	Zfp764
0.38	Sbno2	0.69	Spp1	0.40	Tor2a	1.17	Zmynd15
0.63	Scara3	0.44	Sppl2b	0.63	Tor4a	0.49	Znrd1
0.51	Scara5	3.05	Sprr1a	0.38	Tpgs2	0.95	Zswim3
0.73	Scd2	0.53	Spry4	0.50	Tpm2	0.44	Zswim4
0.44	Scn1b	0.65	Spsb2	0.35	Tpm3	0.55	Zyx

0 — 3.0

Figure 6S-K-O. Supplementing the BCAA-free diet with propionate substitutes for BCAA, enhancing gene expression in the heart during pressure overload. K. RNA was extracted and sequenced (RNA-Seq) from the hearts of mice on a BCAA-free diet or a BCAA-free diet supplemented with 1% propionate, subjected to sham or TAC surgeries. The LFC of the RNA reads from the TAC, BCAA-free plus 1% propionate / TAC, BCAA-free were calculated and those with significant increases in LFC of > 0.3 are presented in the heatmap, showing the LFC values and the gene names for each. The color scale bar for LFC is shown below the heatmap. **L-O.** The genes identified (listed in the heatmap) were analyzed by DAVID Bioinformatics Resources for functional pathways. Some of these pathway networks (KEGG_Pathway) include **L.** ECM-receptor interaction, **M** Signaling in cancer, **N.** Viral protein interaction with cytokine and cytokine receptor, and **O.** Apoptosis. Genes labeled with a red star are from the analysis list**.**

Figure 6S (cont')

L.

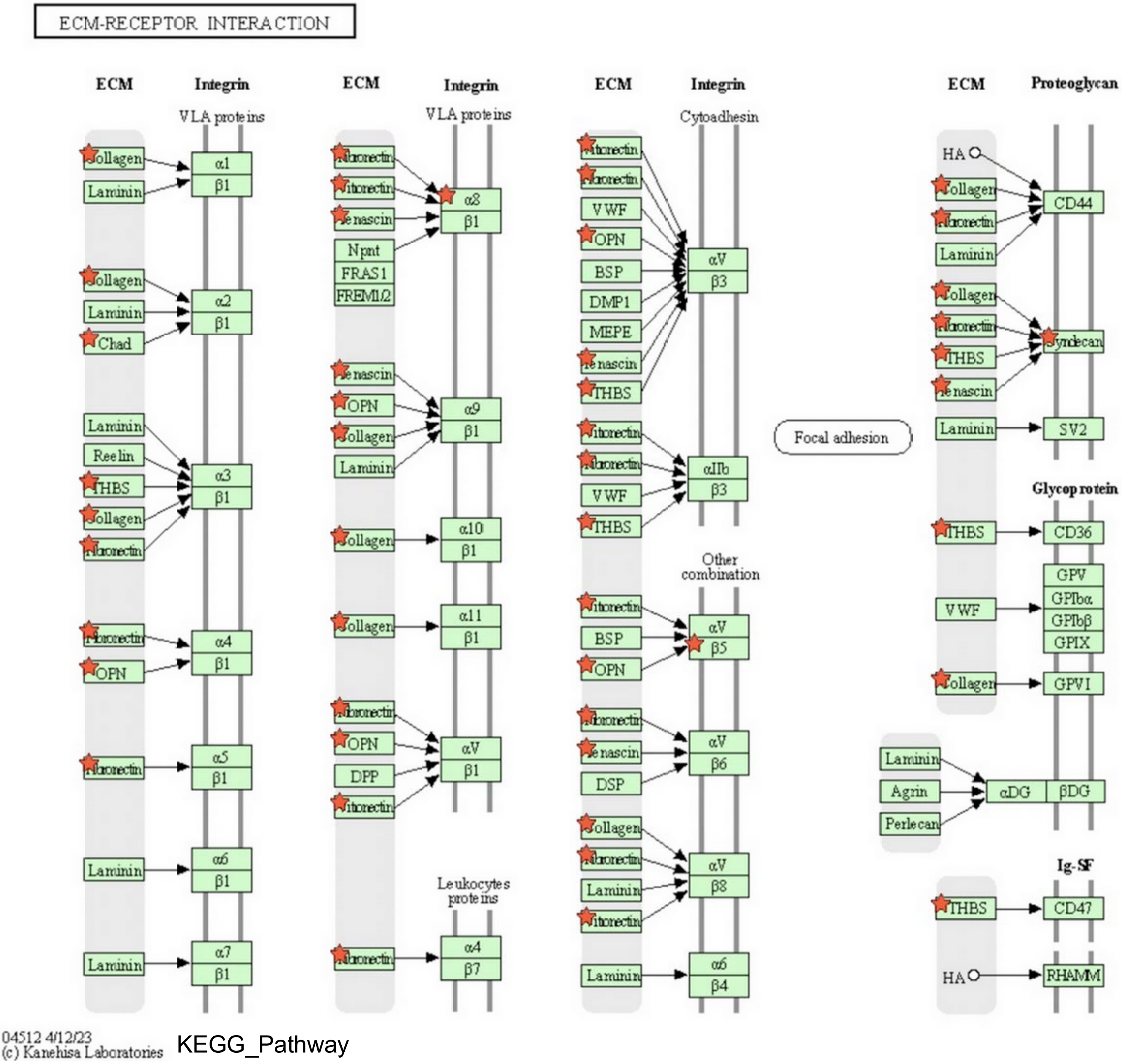

KEGG_Pathway

upregulated by TAC in the heart of mice on a BCAA-free diet plus 1% propionate v. BCAA-free without propionate

Figure 6S (cont')

M.

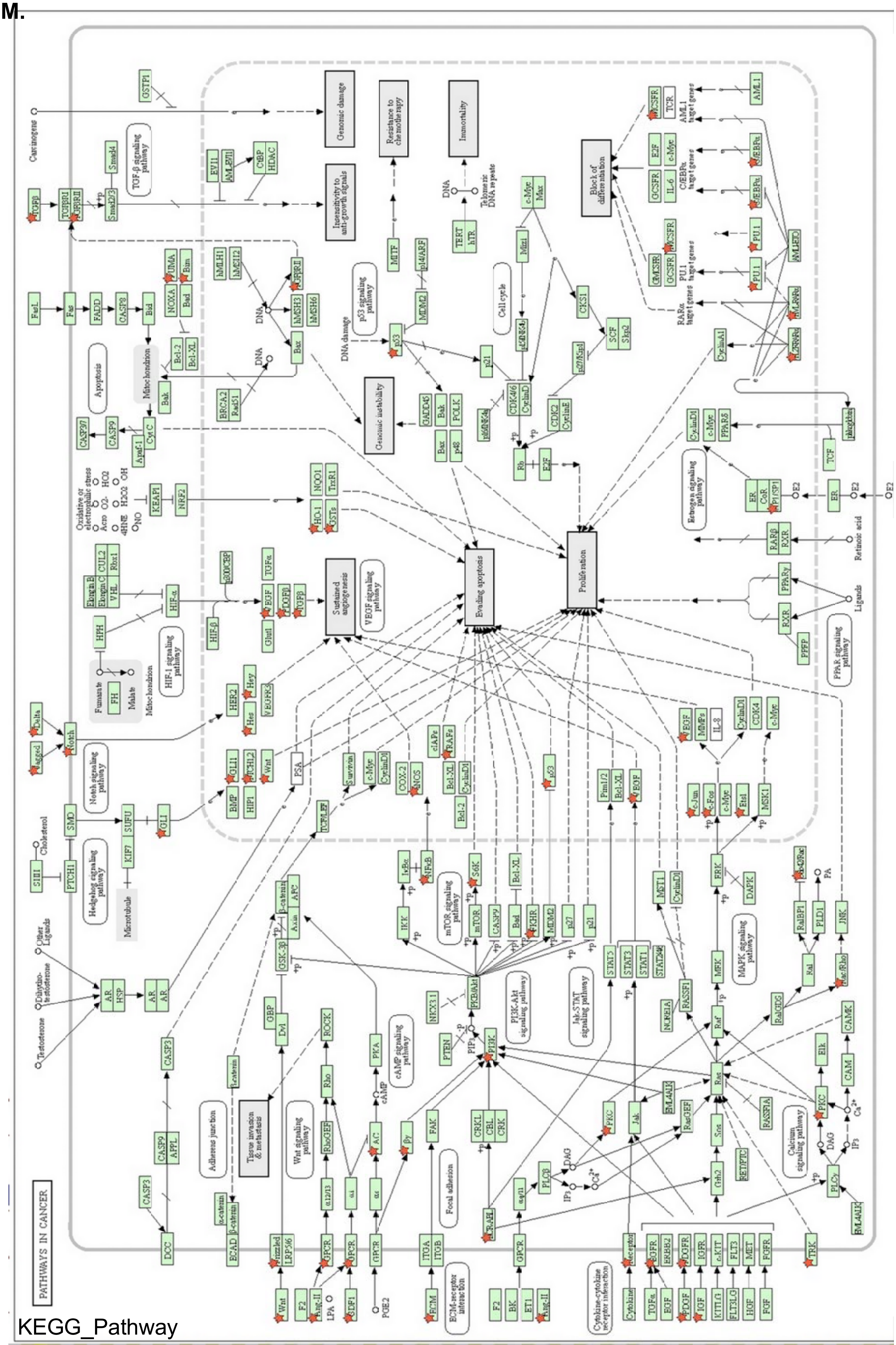

KEGG_Pathway

upregulated by TAC in the heart of mice on a BCAA-free diet plus 1% propionate v. BCAA-free without propionate

Figure 6S (cont')

N.

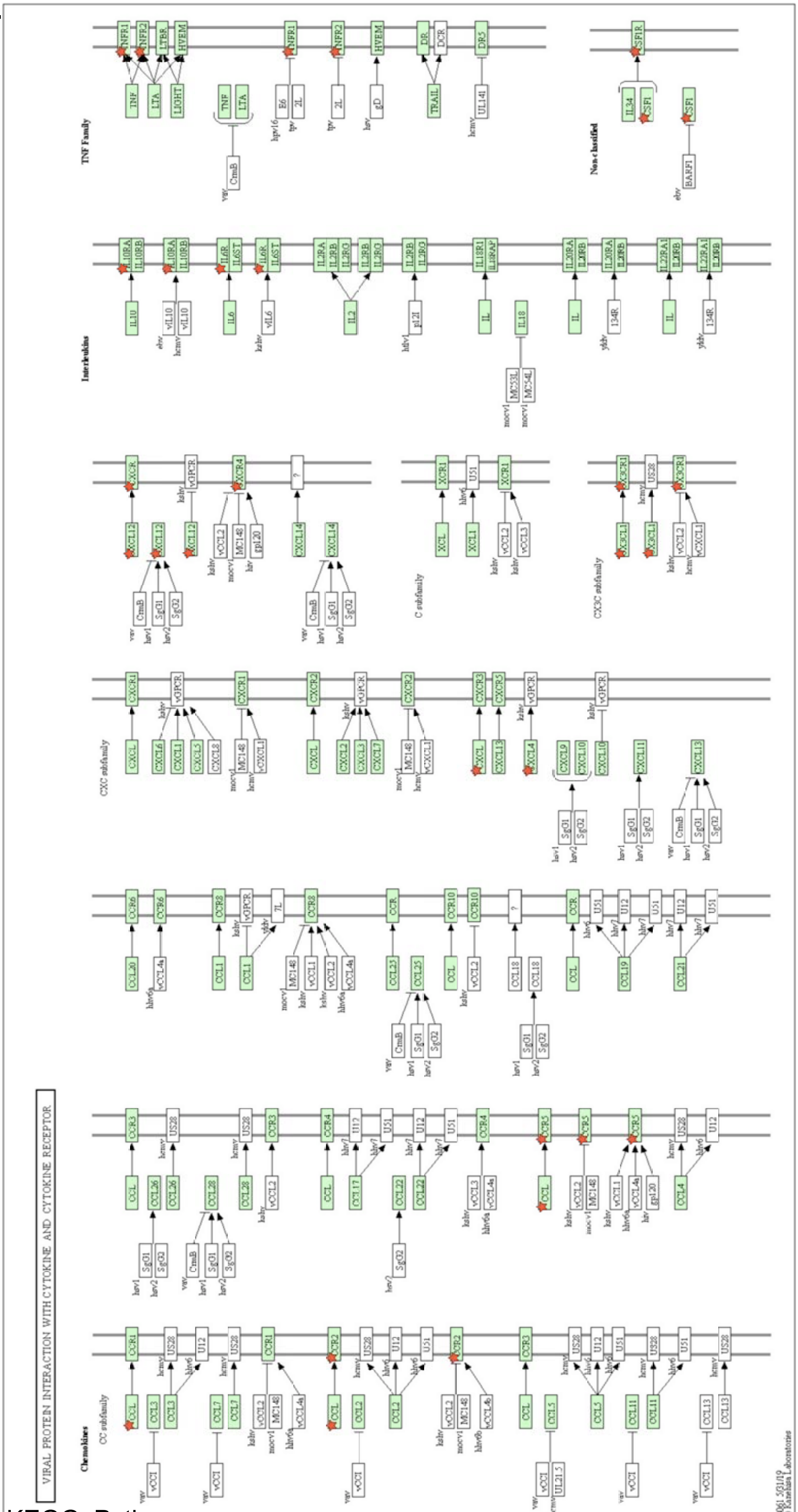

KEGG_Pathway

⭐upregulated by TAC in the heart of mice on a BCAA-free diet plus 1% propionate v. BCAA-free without propionate

Figure 6S (cont')

O.

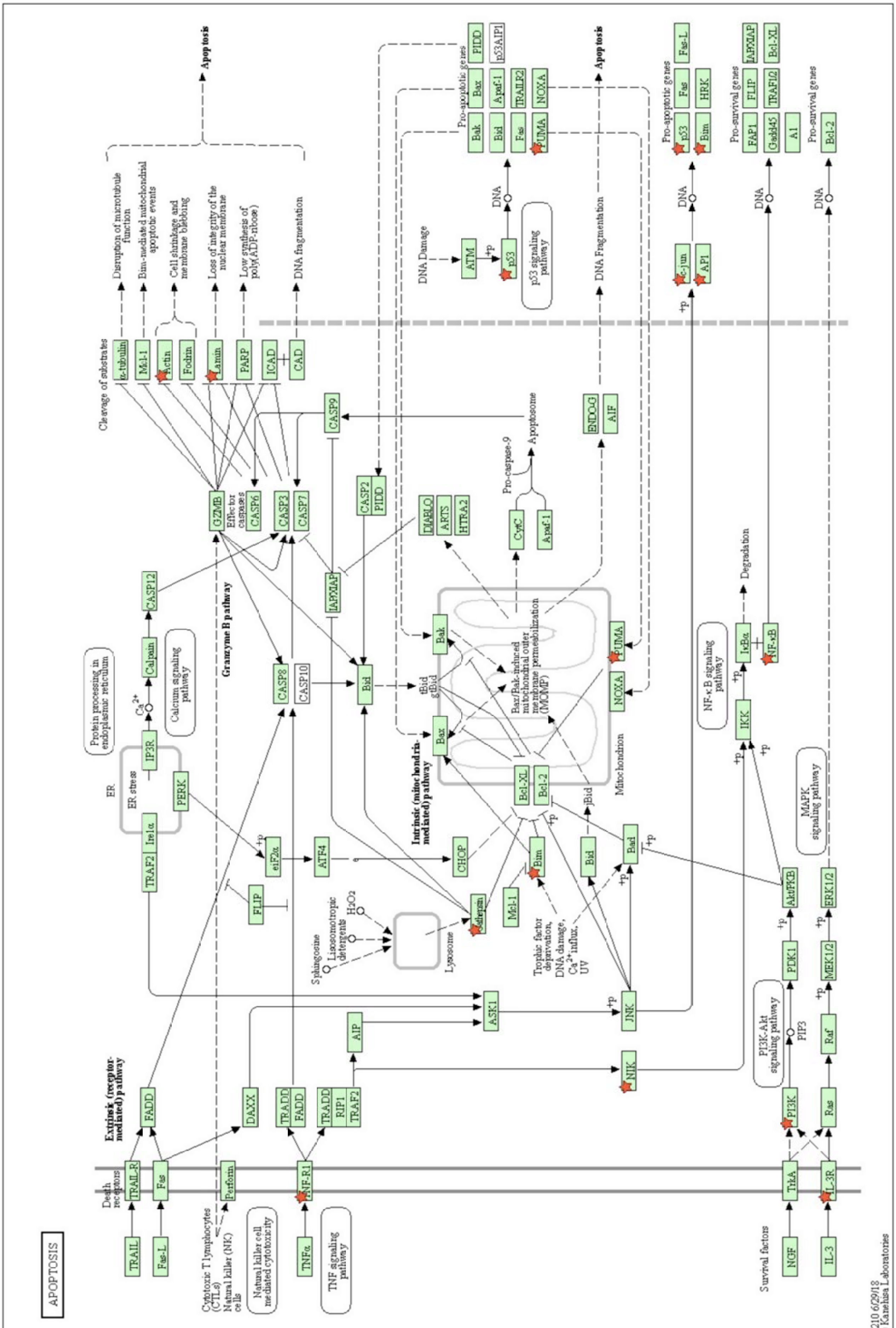

KEGG_Pathway

⭐upregulated by TAC in the heart of mice on a BCAA-free diet plus 1% propionate v. BCAA-free without propionate

Figure 6S (cont')

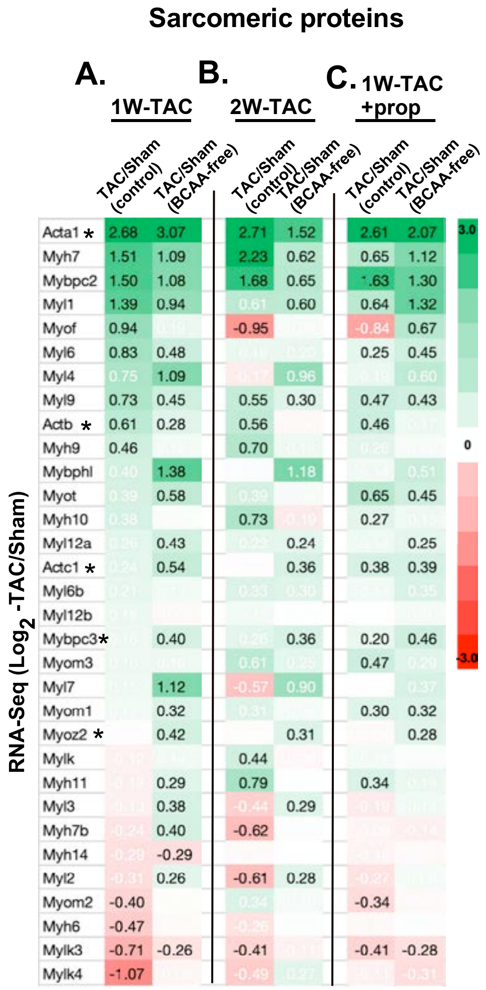

Figure 7S. Pressure overload uniquely increases cardiac actin, myosin binding protein c3, and myozenin2 in the heart, with a BCAA-free vs. BCAA-control diet. Mice were treated as described in figure 2 (in main text) **A., B.** without or **C.** with 1% dietary propionate supplement. **A., C.** 1W or **B.** 2W post-TAC, the hearts were isolated, RNA extracted and sequenced (n=3, each). The heatmaps show the LFC of TAC/Sham (control) and TAC/Sham (BCAA-free), for cardiac sarcomeric proteins. The values displayed in black are those with padj of ≤0.05. The color code bar is shown on the right. **D-I.** Integrated genome browser images showing H3K23Pr ChIP sequence Tags from the **D-F.** 1W, or **G-I.** 2W, sham and TAC hearts of mice on the different diets, aligned across *Actc1, Actb,* and *Acta1* gene coordinates, and sham and TAC hearts aligned across *Actc1*, *Actb*, and *Acta1*. The labels on the left of each track indicate the surgical and diet conditions applied in mice; Sham and TAC surgeries with the BCAA-control diet (blue tracks), with the BCAA-free diets (green tracks); the differences (Diff) in the H3K23Pr sequence tags of TAC minus sham are shown in separate tracks for the control diet (Diff-Control, brown track) and BCAA-free diet (Diff-0BCAA, gold track).

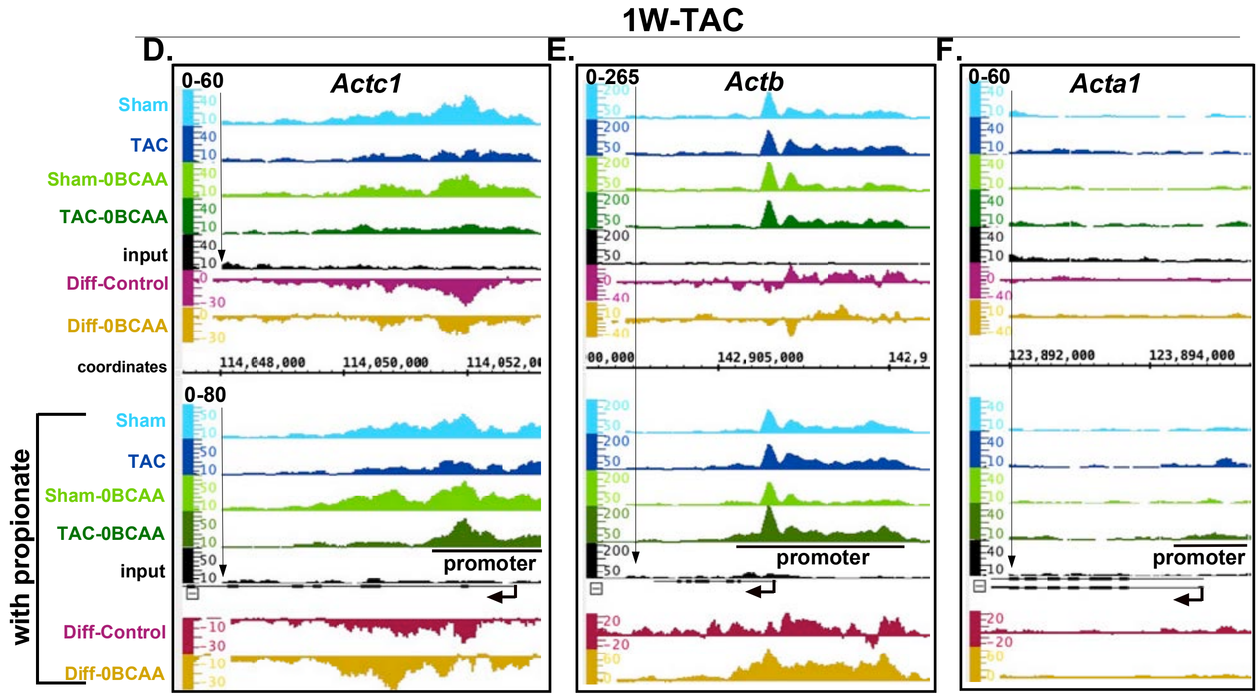

Figure 7S

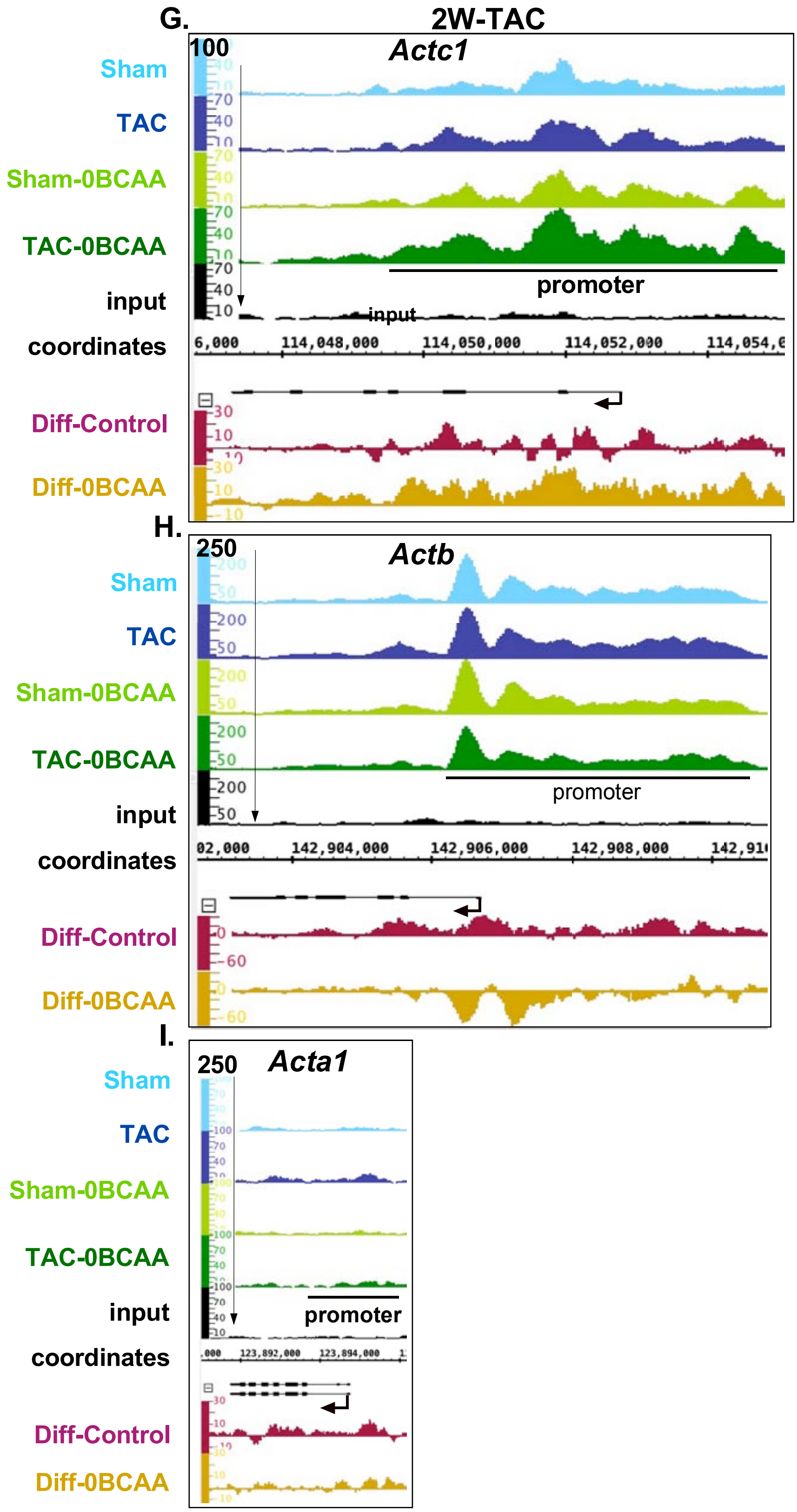

Figure 7S (continued)

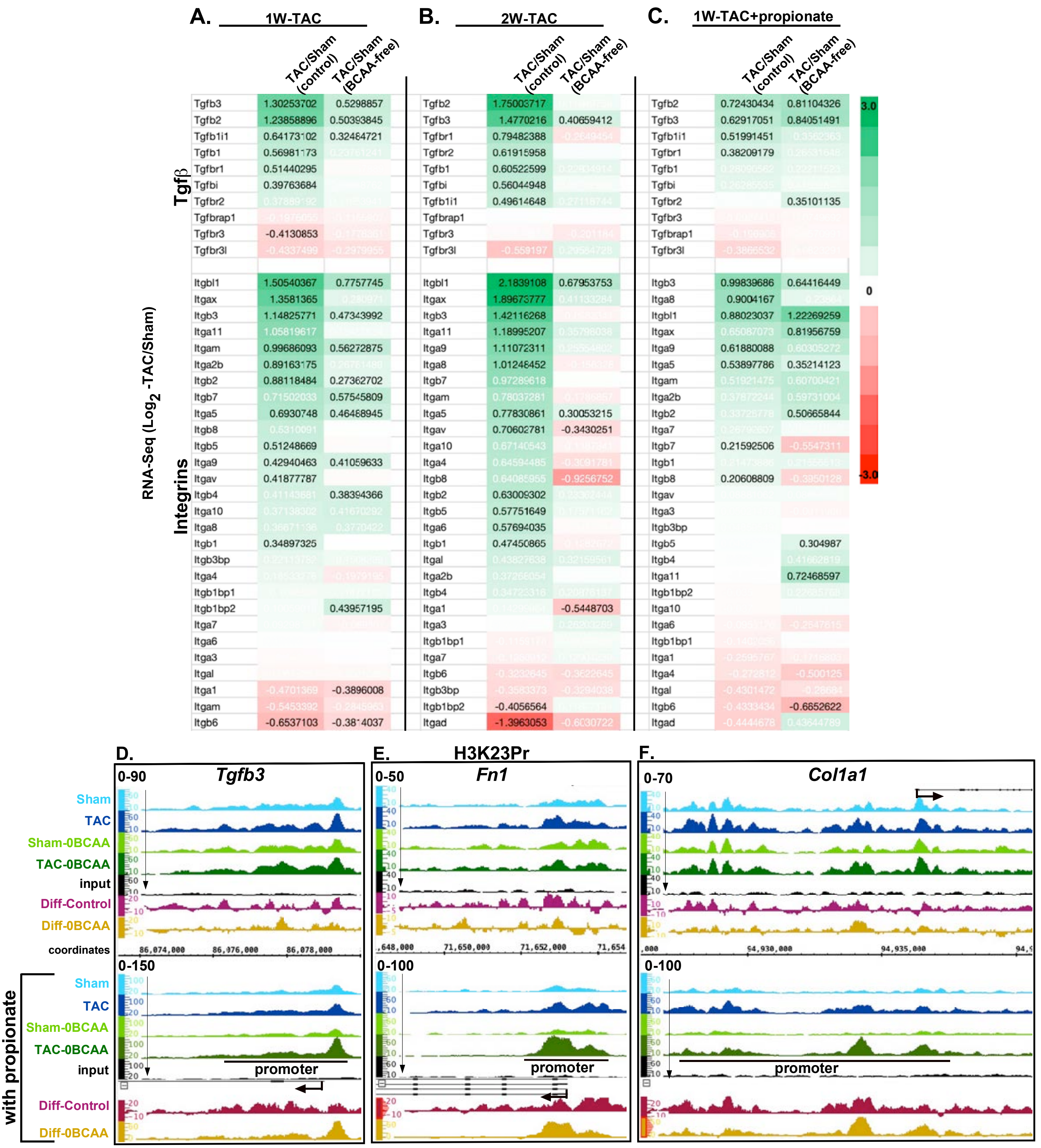

Figure 8S. TGFβ ligands, their receptors, and integrins, are upregulated by pressure overload but significantly dampened by a BCAA-free diet. Mice were treated as described in figure 2 (in main text) **A., B.** without or **C.** with 1% dietary propionate supplement. **A., C.** 1W or **B.** 2W post-TAC, the hearts were isolated, RNA extracted and sequenced (n=3, each). The heatmaps show the LFC of TAC/Sham (control) and TAC/Sham (BCAA-free), of the mRNA for TGFβ receptor ligand and integrin isoforms. The values displayed in black are those with padj of ≤ 0.05. The color code bar is shown on the right. **D-F.** Integrated genome browser images showing H3K23Pr ChIP sequence Tags from the 1W sham and TAC hearts of mice on the different diets, aligned across *Tgfb3*, *Fn1*, and *Col1a1* gene coordinates. See legend of Fig. 7S for more track description.

Figure 8S

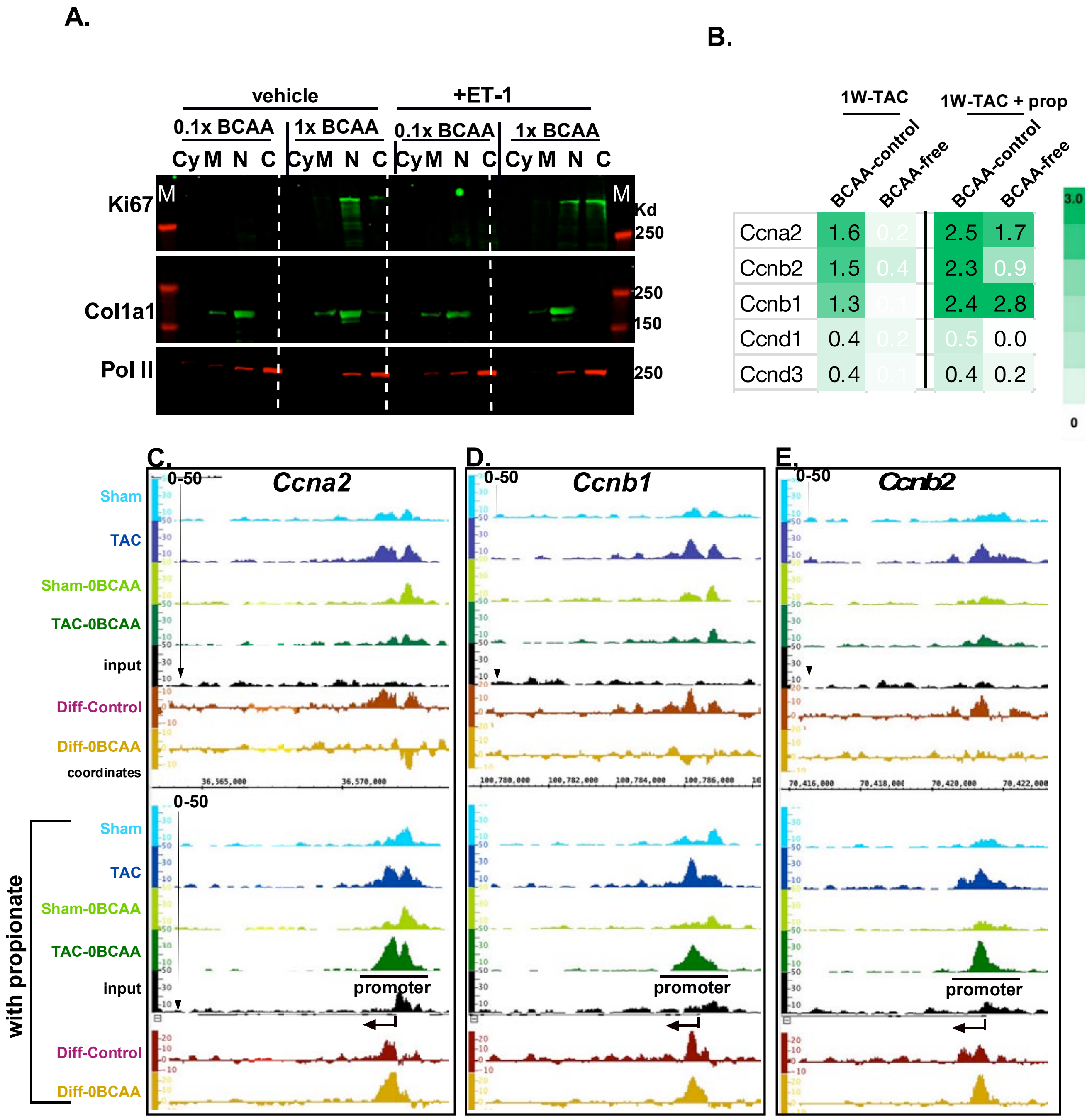

Figure 9S. Low BCAA reduces fibroblast proliferation and expression of Col1a1. Rat cardiac fibroblasts were freshly isolated and cultured in medium with the standard amount of BCAA (1x) or reduced BCAA (0.1x), in the presence or absence of 100 nM endothelin (ET-1). After 20h the protein was extracted, fractionated, and analyzed by Western blots with the Ab for the protein listed on the left. **Cy=cytoplasm, M=membrane, N=nucleoplasm, C=chromatin.** Please note that col1a1 protein is in the ER, which is in continuim with the nuclear membrane, hence its presence in the nucleoplasm fraction. **B.** The heatmaps show the LFC of TAC/Sham (control) and TAC/Sham (BCAA-free) of the mRNA for the proliferations genes Ccna2, Ccnb1, and Ccnb2. The values displayed in black are those with padj of ≤ 0.05. The color code bar is shown on the right. **C-E.** Integrated genome browser images showing H3K23Pr ChIP sequence Tags from the 1W sham and TAC hearts of mice on the different diets, aligned across *Ccna2, Ccnb1, and Ccnb2* gene coordinates. The labels on the left of each track indicate the surgical and diet conditions applied in mice; Sham and TAC surgeries with the BCAA-control diet (blue tracks), with the BCAA-free diets (green tracks); the differences (Diff) in the H3K23Pr sequence tags of TAC minus sham are shown in separate tracks for the control diet (Diff-Control, brown track) and BCAA-free diet (Diff-0BCAA, gold track).

Figure 9S

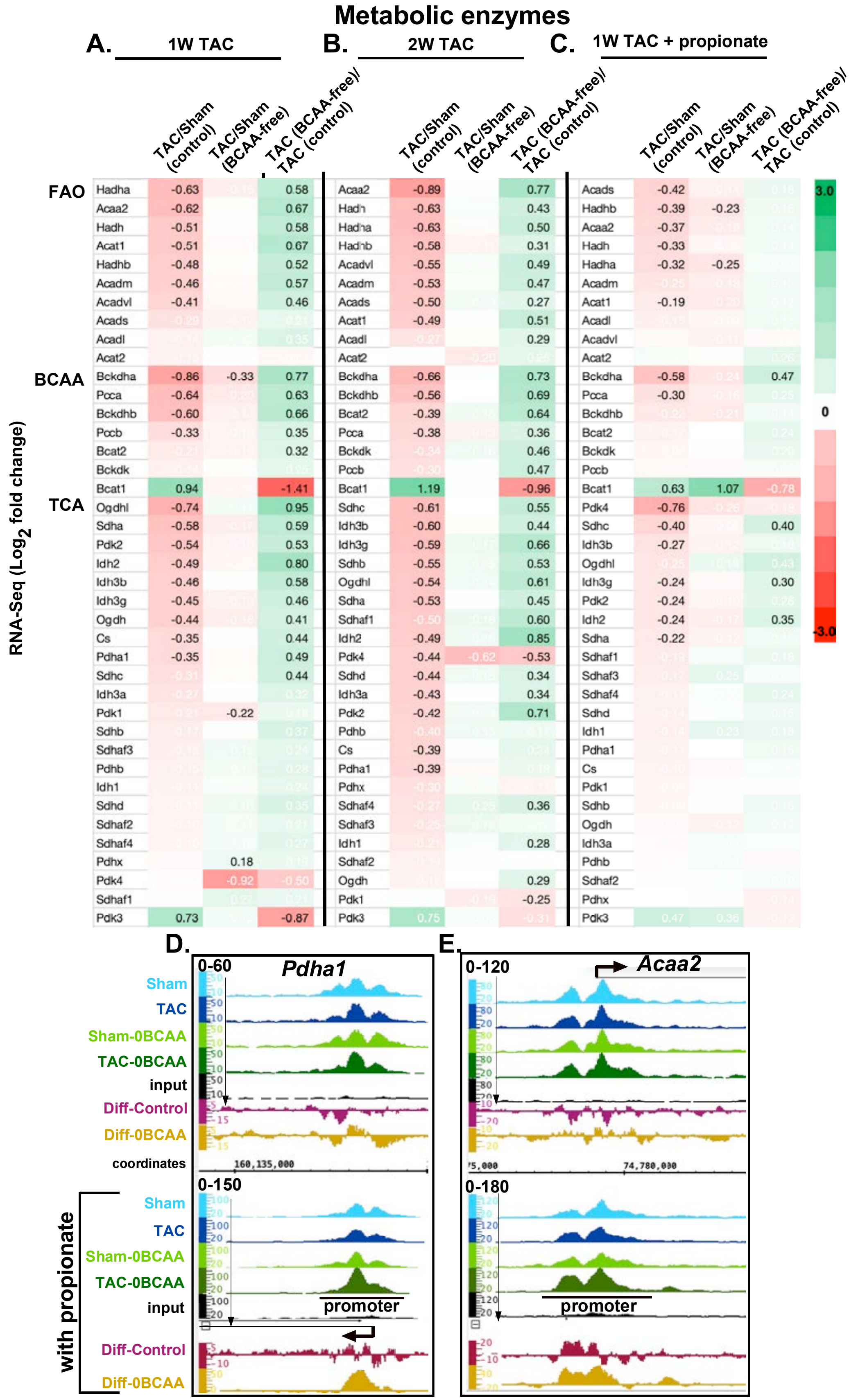

Figure 10S. Metabolic enzymes are downregulated by pressure overload, which is prevented by maintaining the mice on a BCAA-free diet. Mice were treated as described in figure 2 (in main text) **A., B.** without or **C.** with 1% dietary propionate supplement. **A., C.** 1Wk or B. 2W post-TAC, the hearts were isolated, RNA extracted and sequenced (n=3, each). The heatmaps show the LFC of TAC/Sham (control), TAC/Sham (BCAA-free), and TAC (BCAA-free)/TAC (BCAA-control) of the mRNA for metabolic enzymes.The values displayed in black are those with padj of ≤0.05. The color keycode bar is shown on the right. **D-F.** Integrated genome browser images showing H3K23Pr ChIP sequence Tags from the 1W sham and TAC hearts of mice on the different diets, aligned across *Pdha1* and *Acaa2* gene coordinates. See legend of Fig. 7S for track description.

Figure 10S

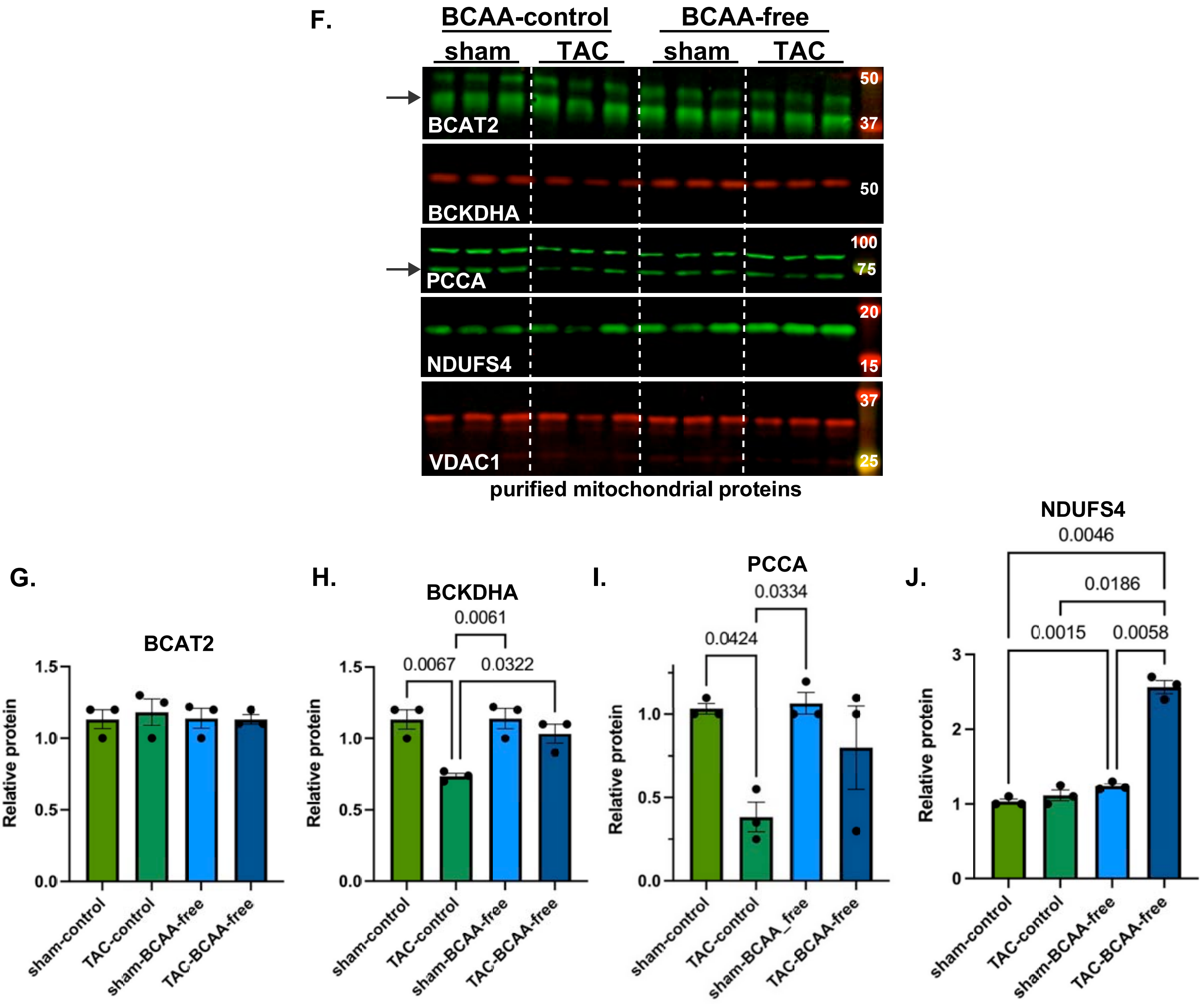

Figure 10S-F-H. Mitochondria were isolated and the protein extracted and analyzed by western blotting with the antibodies listed on the left in each panel. The signals were quantitated and normalized to VDAC1. **G-J.** The results were graphed as relative protein levels, after adjusting one of the the sham control values to 1. Data were analyzed by one-way Anova (n=3, each). The p values of ⩽ 0.05 are listed above the brackets encompassing the bars.

Figure 10S-cont'

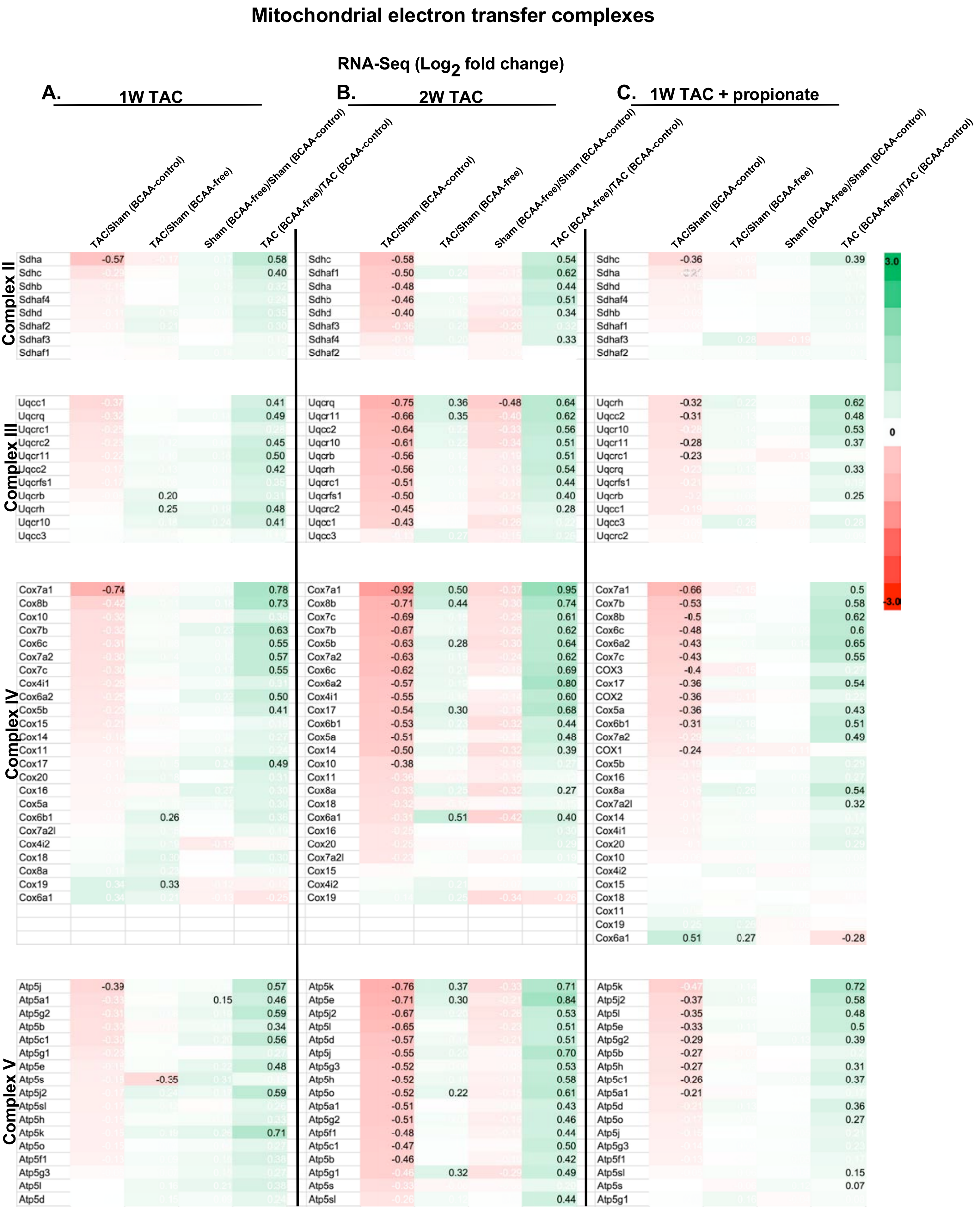
Mitochondrial electron transfer complexes
RNA-Seq (Log2 fold change)
A. 1W TAC
B. 2W TAC
C. 1W TAC + propionate
TAC/Sham (BCAA-control)
TAC/Sham (BCAA-free)
Sham (BCAA-free)/Sham (BCAA-control)
TAC (BCAA-free)/TAC (BCAA-control)
Complex II
Complex III
Complex IV
Complex V
3.0
0
-3.0

Figure 11S

Figure 11S. Mitochondrial ETC subunits are downregulated by pressure overload, which is prevented by maintaining the mice on a BCAA-free diet. Mice were treated as described in figure 2 (main text), **A., B.** without or **C.** with 1% dietary propionate supplement. **A., C.** One week or **B.** 2W post-TAC, the hearts were isolated, RNA extracted and sequenced (n=3, each). The heatmaps show the LFC of TAC/Sham (control), TAC/Sham (BCAA-free), Sham (BCAA-free)/Sham (BCAA-control) and TAC (BCAA-free)/TAC (BCAA-control) of the mRNA for ETC II-IV subunits. The values displayed in black are those with padj of ≤0.05. The color code bar is shown on the right.

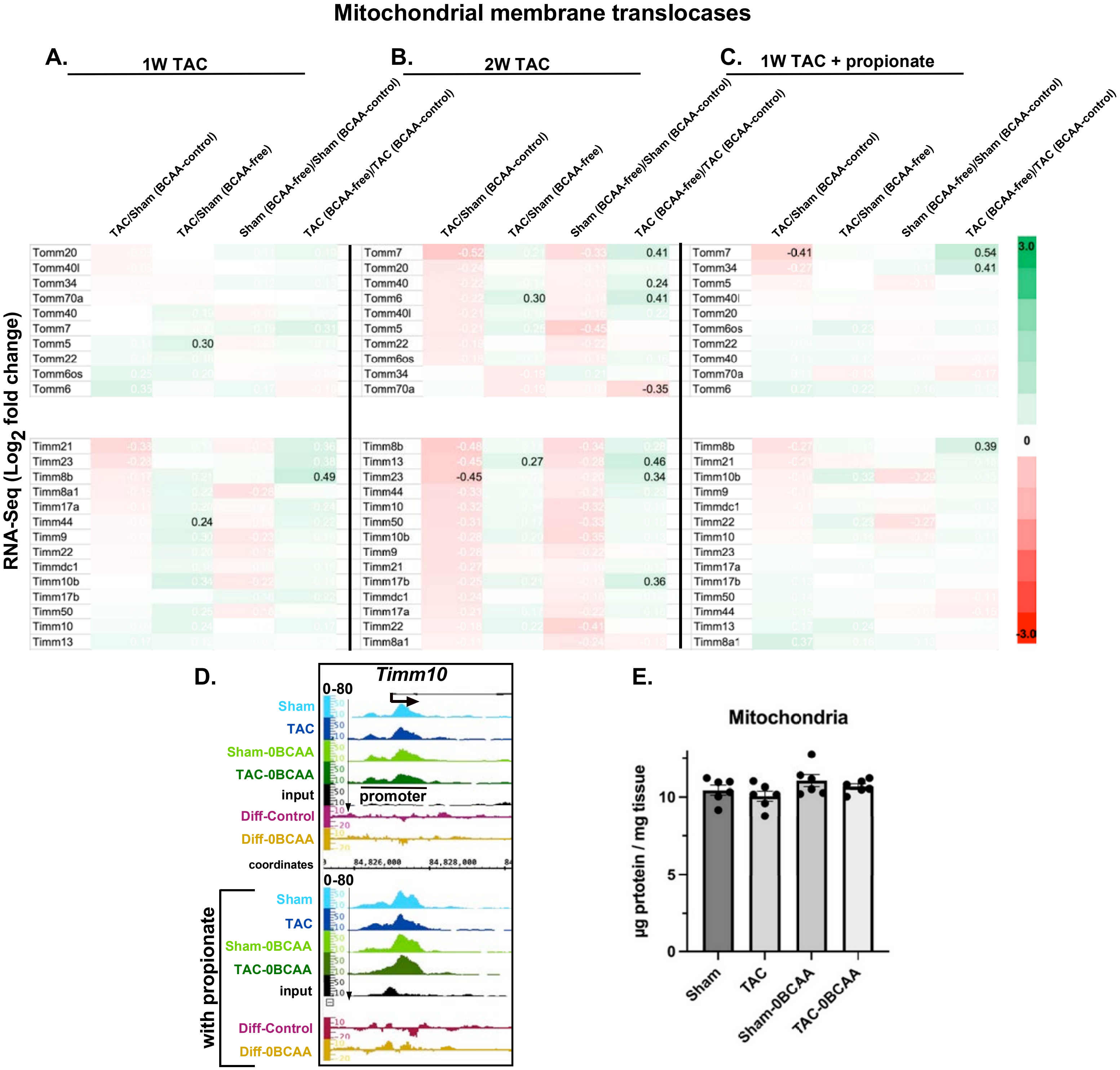

Figure 12S. There are no significant changes in mitochondrial membrane translocases mRNA expression, or total mitochondrial protein, after pressure overload. Mice were treated as described in figure 2 (in main text), **A., B.** without or **C.** with 1% dietary propionate supplement. **A., C.** One week or B. 2W post-TAC, the hearts were isolated, RNA extracted and sequenced (n=3, each). The heatmaps show the LFC of TAC/Sham (control), TAC/Sham (BCAA-free), Sham (BCAA-free)/Sham (BCAA-control) and TAC (BCAA-free)/TAC (BCAA-control) of the mRNA for mitochondrial Tomm and Timm isoforms. The color code bar is shown on the right. **D.** Integrated genome browser images showing H3K23Pr ChIP-sequence Tags from the 1W sham and TAC hearts of mice on the different diets, aligned across *Timm10* gene coordinates. The labels on the left of each track indicate the surgical and diet conditions applied in mice; Sham and TAC surgeries with the BCAA-control diet (blue tracks), with the BCAA-free diets (green tracks); the differences (Diff) in the H3K23Pr sequence tags of TAC minus sham are shown in separate tracks for the control diet (Diff-Control, brown track) and BCAA-free diet (Diff-0BCAA, gold track). **E.** Mitochondria were extracted from LV tissue, protein quantified, normalized to tissue mass, and graphed (n=6). Data were analyzed by one-way Anova, detecting no significant differences. See legend of Fig. 7S for track description.

Figure 12S

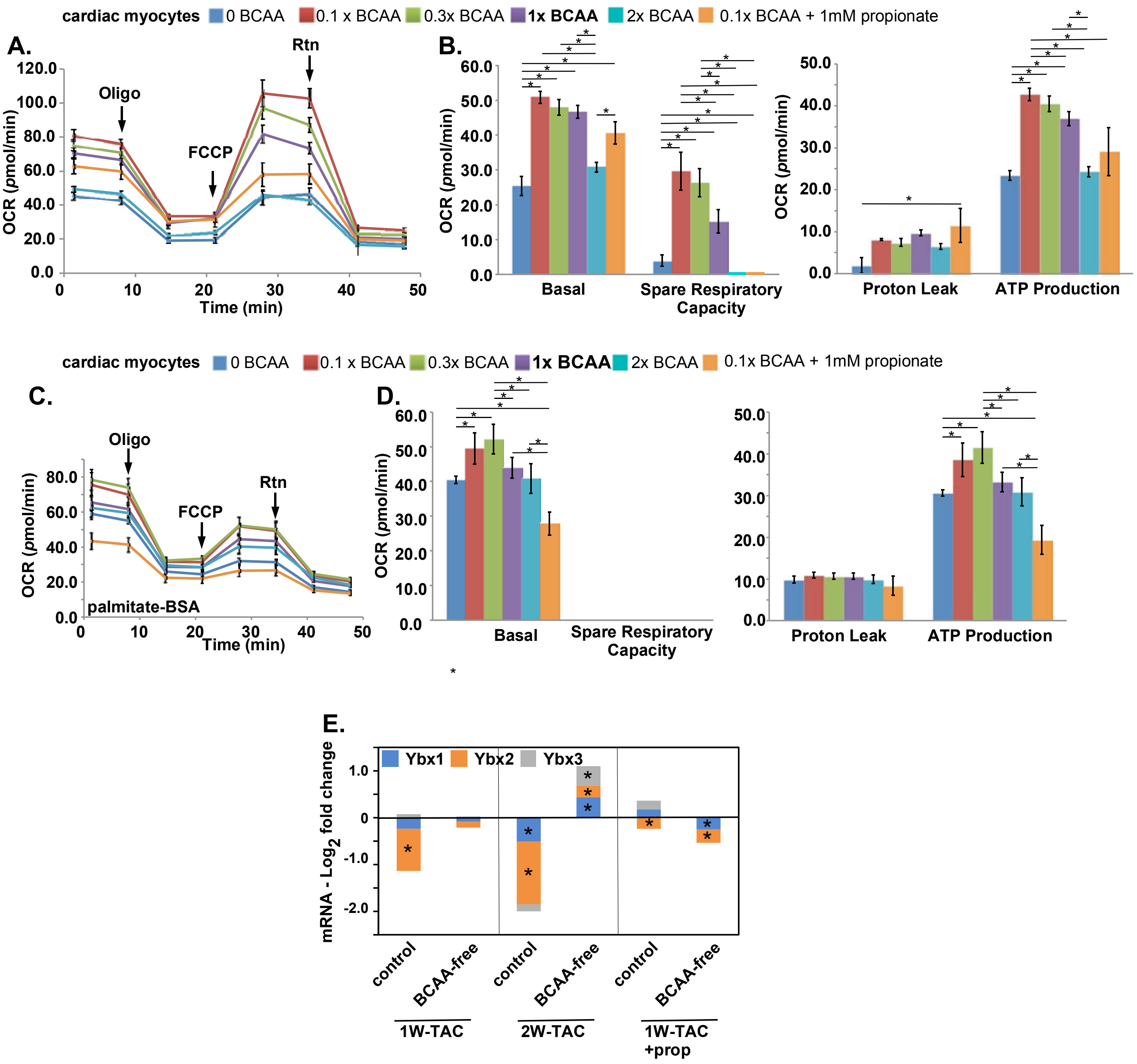

Figure 13S. Low BCAA enhances respiration. A. Neonatal rat cardiac myocytes were cultured in DMEM (with glucose) with either 1x or 0.1x BCAA, or 0.1x of the individual amino acids Leu, Ile, or Val, as indicated by the keycode. After 16 h, OCR (pmol/min, Y-axis) over time (X-axis) was measured by the Seahorse extracellular analyzer in the intact cells, before and after addition of oligomycin (oligo), FCCP, and rotenone (Rtn), where indicated by the arrows on the curve. **B.** The mitochondrial spare respiratory capacity, proton leak, and ATP-linked OCRs were calculated and graphed (n=3 independent cultures, n=10 replicas, each). Error bars represent S.E.M, *p ≤0.05, analyzed by one-way Anova.**C.** Neonatal rat cardiac myocytes were cultured in DMEM (with 100 μM palmitate-BSA, no glucose) with increasing doses of BCAA as indicated in the color keycode. After 16 h, OCR's (pmol/min, Y-axis) over time (X-axis) were measured by the Seahorse analyzer in the intact cells before and after addition of oligomycin (oligo), FCCP, and rotenone (Rtn), where indicated by the arrows on the curve. **D.** The mitochondrial basal, spare respiratory capacity, proton leak, and ATP-linked OCRs were calculated and graphed (n=3 independent cultures; n=10 measurements, each). Error bars represent S.E.M, *p ≤ 0.05, analyzed by one-way Anova. **C.** Stacked bar graph of the LFC of TAC/sham mRNA expression of the Ybx isoforms, with the different diets, 1W and 2W post-TAC, * = padj ≤ 0.05.

Figure 13S

CD surface markers

RNA-Seq (Log2-TAC/Sham)

Color scale: 3.0 / 0 / -3.0

A. 1W-TAC

	TAC/Sham (control)	TAC/Sham (BCAA-free)
Cd72	1.49935452	0.78390431
Cd300c2	1.44666415	0.56996036
Cd52	1.29793755	0.9130529
Cd80	1.27141798	0.6446058
Cd84	1.18165239	0.42799266
Cd300lb	1.18120776	0.72272213
Cd200r1	1.10820672	0.67518669
Cd68	0.9546788	0.50643049
Cd276	0.93603022	[illegible]
Cd180	0.93574094	[illegible]
Cd109	0.84442289	
Cd300a	0.82741947	[illegible]
Cd248	0.81718066	0.58168402
Cd24a	0.81359382	[illegible]
Cd53	0.74396309	0.4017447
Cd302	0.73077849	0.31461082
Cd34	0.65829212	0.306704
Cd44	0.63298335	[illegible]
Cd40	0.61586137	0.57483616
Cd48	0.61540414	[illegible]
Cd63	0.60071127	[illegible]
Cd9	0.57548401	0.31693279
Cd300ld	[illegible]	[illegible]
Cd14	0.55934308	[illegible]
Cd86	[illegible]	[illegible]
Cd37	[illegible]	[illegible]
Cd38	[illegible]	[illegible]
Cd3eap	[illegible]	[illegible]
Cd83	[illegible]	[illegible]
Cd200	[illegible]	0.2822309
Cd33	[illegible]	[illegible]
Cd93	[illegible]	
Cd163	[illegible]	[illegible]
Cd63-ps	[illegible]	[illegible]
Cd81	[illegible]	
Cd47	[illegible]	
Cd151	[illegible]	
Cd2bp2	[illegible]	
Cd320	[illegible]	
Cd55	[illegible]	[illegible]
Cd82	0.02725965	-0.4432003
Cd74		
Cd300lg		
Cd164	[illegible]	[illegible]
Cd1d1	[illegible]	[illegible]
Cd2ap	[illegible]	[illegible]
Cd99l2	[illegible]	-0.2137939
Cd59a	[illegible]	
Cd19	[illegible]	0.5629617
Cd59b	[illegible]	[illegible]
Cd36	-0.3796084	-0.1773157
Cd46	-0.6032461	[illegible]
Cd28	-0.7667597	-0.6284756
Cd274	-0.8339336	[illegible]

B. 2W-TAC

	TAC/Sham (control)	TAC/Sham (BCAA-free)
Cd200r1	1.87545715	[illegible]
Cd109	1.87154067	[illegible]
Cd72	1.47081871	[illegible]
Cd44	1.36809686	[illegible]
Cd52	0.9506423	[illegible]
Cd276	0.94597941	
Cd248	0.91228761	0.56237895
Cd74	0.85882564	0.4127959
Cd300c2	0.84878333	[illegible]
Cd300lb	0.74886571	
Cd34	0.71458034	[illegible]
Cd9	0.67602011	
Cd83	0.66697834	[illegible]
Cd84	0.66466318	[illegible]
Cd93	0.65406507	-0.2793776
Cd53	0.6491451	
Cd48	0.59219711	[illegible]
Cd80	0.58391335	[illegible]
Cd300a	0.57591967	[illegible]
Cd38	0.55976756	
Cd86	0.55037205	[illegible]
Cd180	0.53585034	[illegible]
Cd302	0.52105308	
Cd33	0.48214353	
Cd300ld	0.46092674	[illegible]
Cd55	0.40268643	
Cd14	0.38508342	[illegible]
Cd68	0.3344773	0.4321922
Cd63	0.33120912	0.27419687
Cd200	[illegible]	[illegible]
Cd40	[illegible]	0.39677862
Cd37	[illegible]	0.5557499
Cd63-ps	[illegible]	[illegible]
Cd24a	[illegible]	[illegible]
Cd47	[illegible]	[illegible]
Cd2ap	[illegible]	-0.3219998
Cd1d1	[illegible]	[illegible]
Cd2bp2		[illegible]
Cd300lg		[illegible]
Cd81		
Cd164		[illegible]
Cd3eap		
Cd19		[illegible]
Cd163		[illegible]
Cd151	[illegible]	[illegible]
Cd82	[illegible]	
Cd99l2	[illegible]	[illegible]
Cd274	[illegible]	[illegible]
Cd36	[illegible]	-0.31739
Cd46	[illegible]	-0.6552272
Cd28	[illegible]	[illegible]
Cd59a	-0.4654219	[illegible]
Cd320	[illegible]	[illegible]
Cd59b	-1.0860528	[illegible]

C. 1W-TAC+propionate

	TAC/Sham (control)	TAC/Sham (BCAA-free)
Cd72	1.18971249	1.12001013
Cd109	1.16587214	[illegible]
Cd84	0.89697512	0.82361162
Cd180	[illegible]	1.06277853
Cd68	0.65797777	0.74407307
Cd300c2	[illegible]	1.42830331
Cd44	0.65530665	[illegible]
Cd63-ps	[illegible]	
Cd14	[illegible]	0.76028149
Cd63	0.38406394	0.23863764
Cd34	0.33181926	0.41973639
Cd163	[illegible]	0.59271607
Cd53	[illegible]	[illegible]
Cd248	[illegible]	0.77388877
Cd52	[illegible]	1.22515166
Cd276	[illegible]	[illegible]
Cd37	[illegible]	[illegible]
Cd200	0.2315693	[illegible]
Cd40	[illegible]	[illegible]
Cd3eap	[illegible]	[illegible]
Cd33	[illegible]	0.67648124
Cd86	[illegible]	0.66037912
Cd300ld	[illegible]	0.71068998
Cd302	[illegible]	0.53771809
Cd47	[illegible]	[illegible]
Cd9		[illegible]
Cd93		[illegible]
Cd320		[illegible]
Cd151		[illegible]
Cd2bp2		[illegible]
Cd24a		[illegible]
Cd81		
Cd59a		[illegible]
Cd164	[illegible]	[illegible]
Cd55	[illegible]	[illegible]
Cd2ap	[illegible]	[illegible]
Cd82	[illegible]	[illegible]
Cd28	[illegible]	-0.6849186
Cd38	[illegible]	
Cd48	[illegible]	0.55188075
Cd1d1	[illegible]	[illegible]
Cd300lg	-0.3888625	[illegible]
Cd36	-0.3990763	[illegible]
Cd59b	[illegible]	
Cd300a	[illegible]	0.40093423
Cd74	-0.4802997	0.49232255
Cd83	-0.610113	0.70101299
Cd46	-0.6124606	
Cd274	-0.9417366	[illegible]
Cd8a	-1.7746362	-1.2292672

D.

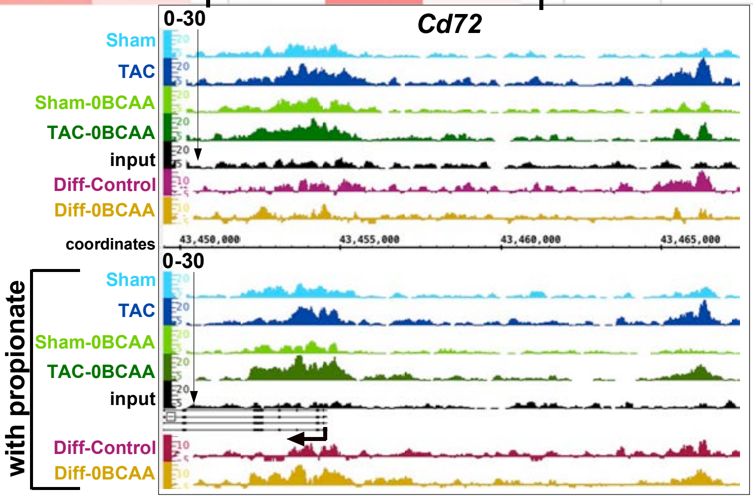

Figure 14S

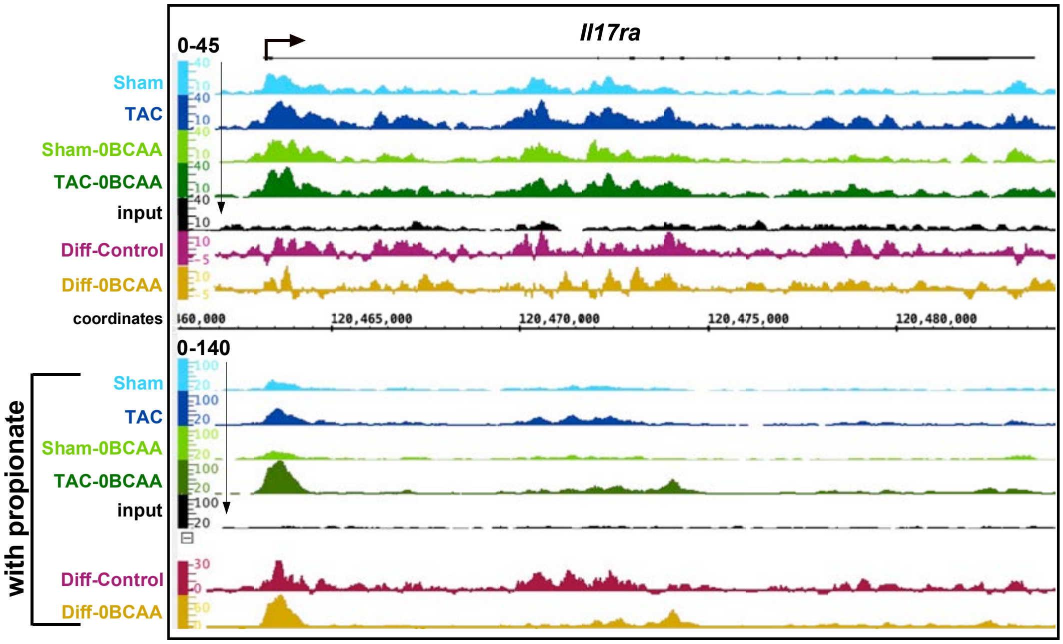

Figure 14S. CD surface markers of infiltrating cells increase after pressure overload and are diminished by a BCAA-free diet. Mice were treated as described in figure 2 (in main text), **A., B.** without or **C.** with 1% dietary propionate supplement. **A., C.** One week or **B.** 2W post-TAC, the hearts were isolated, RNA extracted and sequenced (n=3, each). The heatmaps show the LFC of TAC/Sham (control) and TAC/Sham (BCAA-free), of the mRNA for CD surface markers detect in the heart. The values displayed in black are those with padj of ≤0.05. The color keycode bar is shown on the right. **D-E.** Integrated genome browser images showing H3K23Pr ChIP-sequence Tags from the 1W sham and TAC hearts of mice on the different diets, aligned across *CD72* and *Il17ra* gene coordinates. The labels on the left of each track indicate the surgical and diet conditions applied in mice; Sham and TAC surgeries with the BCAA-control diet (blue tracks), with the BCAA-free diets (green tracks); the differences (Diff) in the H3K23Pr sequence tags of TAC minus sham are shown in separate tracks for the control diet (Diff-Control, brown track) and BCAA-free diet (Diff-0BCAA, gold track).

Figure 14S-F-G

Growth factors

RNA-Seq (Log_2 -TAC/Sham)

Color scale: 3.0 / 0 / -3.0

A. 1W-TAC

	TAC/Sham (control)	TAC/Sham (BCAA-free)
**Edn**		
Edn3	1.62530857	1.39387517
Ednrb	0.31399216	
Edn1	0.28172786	[illegible]
Ednra	[illegible]	[illegible]
**Igf**		
Igfbp2	3.64852668	4.71825557
Igf1	1.40546612	0.43969003
Igfbp7	1.35751524	0.88346843
Igfbp4	0.63791058	[illegible]
Igfbp5	0.50646763	0.24002208
Igfbp6	0.45274863	0.25809178
Igf1r	0.27756432	[illegible]
Igf2bp2	[illegible]	-0.2037872
Igf2	[illegible]	-0.2707961
Igfbp3	[illegible]	-0.1753515
Igf2r	-0.1667922	-0.211239
Igfals	-0.3282916	[illegible]
Igf2bp3	-0.6315656	
**Vegf**		
Vegfd	0.97744575	0.48446202
Vegfc	[illegible]	[illegible]
Vegfb	-0.1514513	[illegible]
Vegfa	[illegible]	0.18132571
**Adrenergic receptors**		
Adrb2	0.41164795	[illegible]
Adra2b	[illegible]	0.25159361
Adra2c	-0.2765987	[illegible]
Adrb1	-0.4218493	
Adra1b	-0.483103	-0.6169383
Adra1d	-0.6594267	-0.3916688
Adra1a	-1.1224723	-0.605635

B. 2W-TAC

	TAC/Sham (control)	TAC/Sham (BCAA-free)
**Edn**		
Edn3	1.65876559	0.81747889
Edn1	0.84513071	-0.3948743
Ednrb	0.67461344	
Ednra	[illegible]	[illegible]
**Igf**		
Igfbp2	5.5826357	3.99017863
Igfbp7	1.2547204	1.06807543
Igf1	1.0461402	[illegible]
Igfbp5	0.71872264	
Igf1r	0.67878979	-0.2524446
Igfbp6	0.62396191	0.39130075
Igf2bp2	0.50213379	-0.4084851
Igfbp3	0.29816394	[illegible]
Igfbp4	[illegible]	[illegible]
Igf2r	[illegible]	[illegible]
Igf2	[illegible]	[illegible]
Igf2bp3	-0.3135142	[illegible]
Igfals	-0.4717516	[illegible]
**Vegf**		
Vegfd	0.5841975	[illegible]
Vegfc	[illegible]	0.24414132
Vegfa	-0.3882536	[illegible]
Vegfb	-0.3918732	0.64009199
**Adrenergic receptors**		
Adra2b	0.50701715	[illegible]
Adrb2		0.37262458
Adra1d	[illegible]	
Adrb1	-0.5803879	0.3546499
Adra1a	-0.7933284	-0.3660873
Adra1b	-0.8378833	
Adra2c	-0.8471869	[illegible]

C. 1W-TAC+propionate

	TAC/Sham (control)	TAC/Sham (BCAA-free)
**Edn**		
Edn3	1.73588632	0.90697461
Ednra	[illegible]	
Ednrb	[illegible]	-0.2247706
Edn1	-0.2958148	[illegible]
**Igf**		
Igfbp2	5.34010016	4.5964414
Igfbp7	0.94223816	0.75049171
Igf1	0.86465908	1.2120946
Igf2bp3	0.28215287	[illegible]
Igfbp5	0.25271762	0.2361852
Igfbp4	[illegible]	0.33625298
Igf2bp2	[illegible]	[illegible]
Igf1r	[illegible]	[illegible]
Igfals	[illegible]	
Igf2r	[illegible]	
Igfbp6	[illegible]	0.66990566
Igfbp3	[illegible]	[illegible]
Igf2	-0.2403726	
**Vegf**		
Vegfd	0.64814903	0.60737685
Vegfa		-0.210018
Vegfb		[illegible]
Vegfc	-0.4578987	[illegible]
**Adrenergic receptors**		
Adrb2	[illegible]	
Adrb1	[illegible]	-0.4073444
Adra2b	[illegible]	[illegible]
Adra1a	[illegible]	-0.4286321
Adra1d	[illegible]	[illegible]
Adra1b	-0.3565986	-0.4810904
Adra2c	-0.4536829	[illegible]

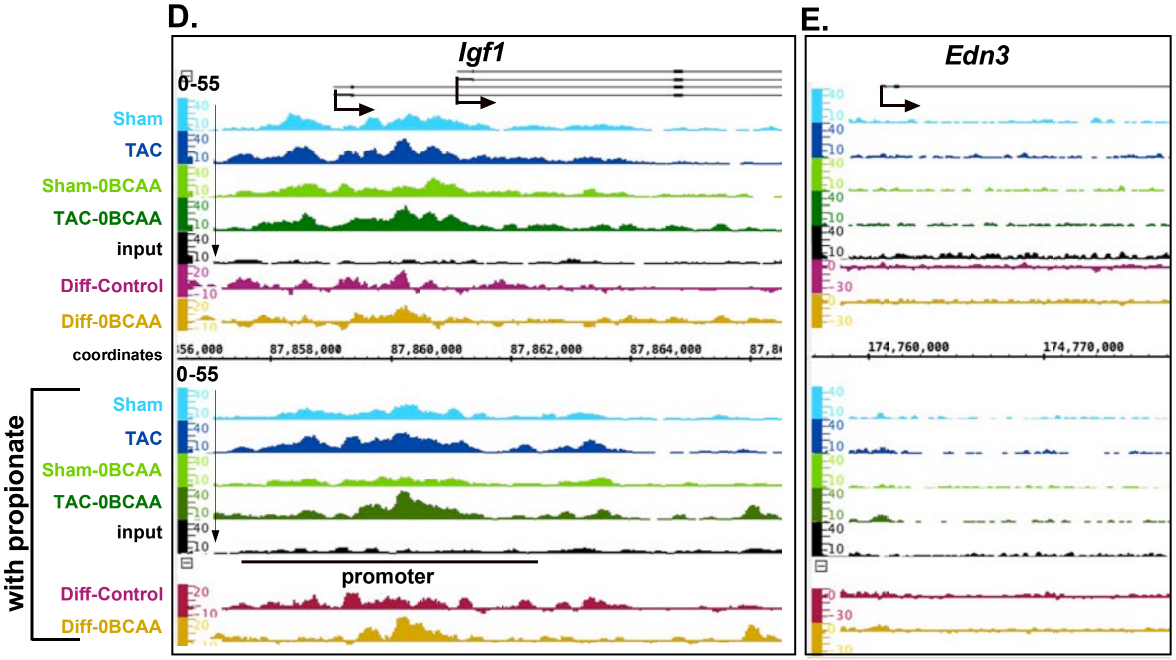

Figure 15S

F.

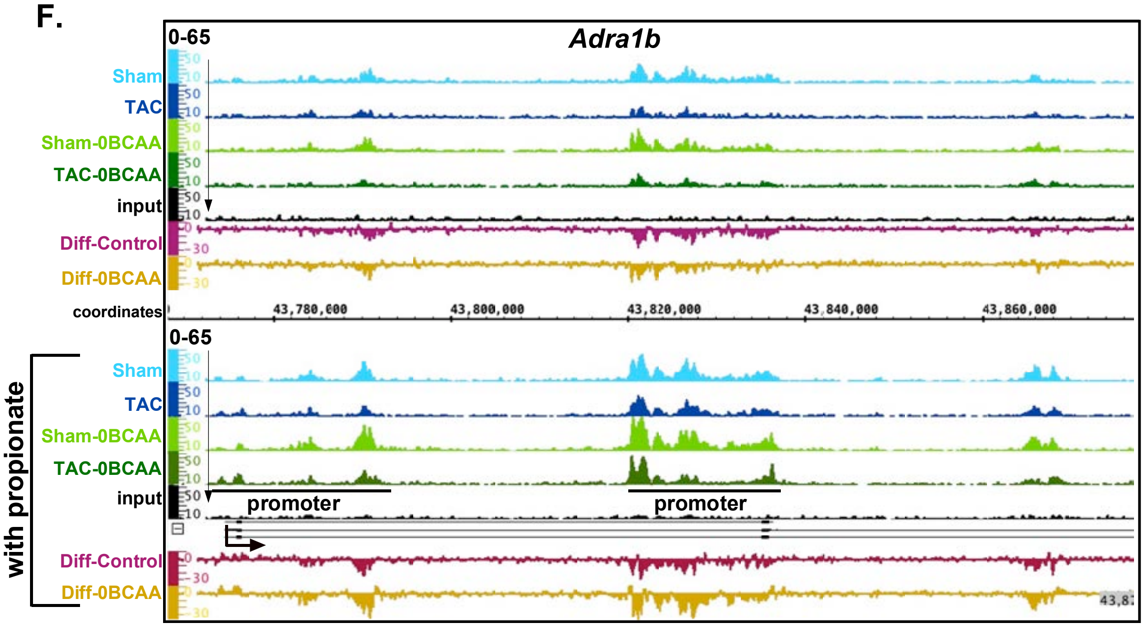

Figure 15S. Selective regulation of growth factors and their receptors by dietary BCAA. Mice were treated as described in figure 2 (in the main text), **A., B.** without or **C.** with 1% dietary propionate supplement. **A., C.** One week or **B.** 2W post-TAC, the hearts were isolated, RNA extracted and sequenced (n=3, each). The heatmaps show the LFC of TAC/Sham (control) and TAC/Sham (BCAA-free), of the mRNA for growth factors and their receptors detect in the heart. The values displayed in black are those with padj of ≤0.05. The color keycode bar is shown on the right. **D-F.** Integrated genome browser images showing H3K23Pr ChIP-sequence Tags from the 1W sham and TAC hearts of mice on the different diets, aligned across *Igf1, End3*, and *Adra1b* gene coordinates. The labels on the left of each track indicate the surgical and diet conditions applied in mice; Sham and TAC surgeries with the BCAA-control diet (blue tracks), with the BCAA-free diets (green tracks); the differences (Diff) in the H3K23Pr sequence tags of TAC minus sham are shown in separate tracks for the control diet (Diff-Control, brown track) and BCAA-free diet (Diff-0BCAA, gold track).

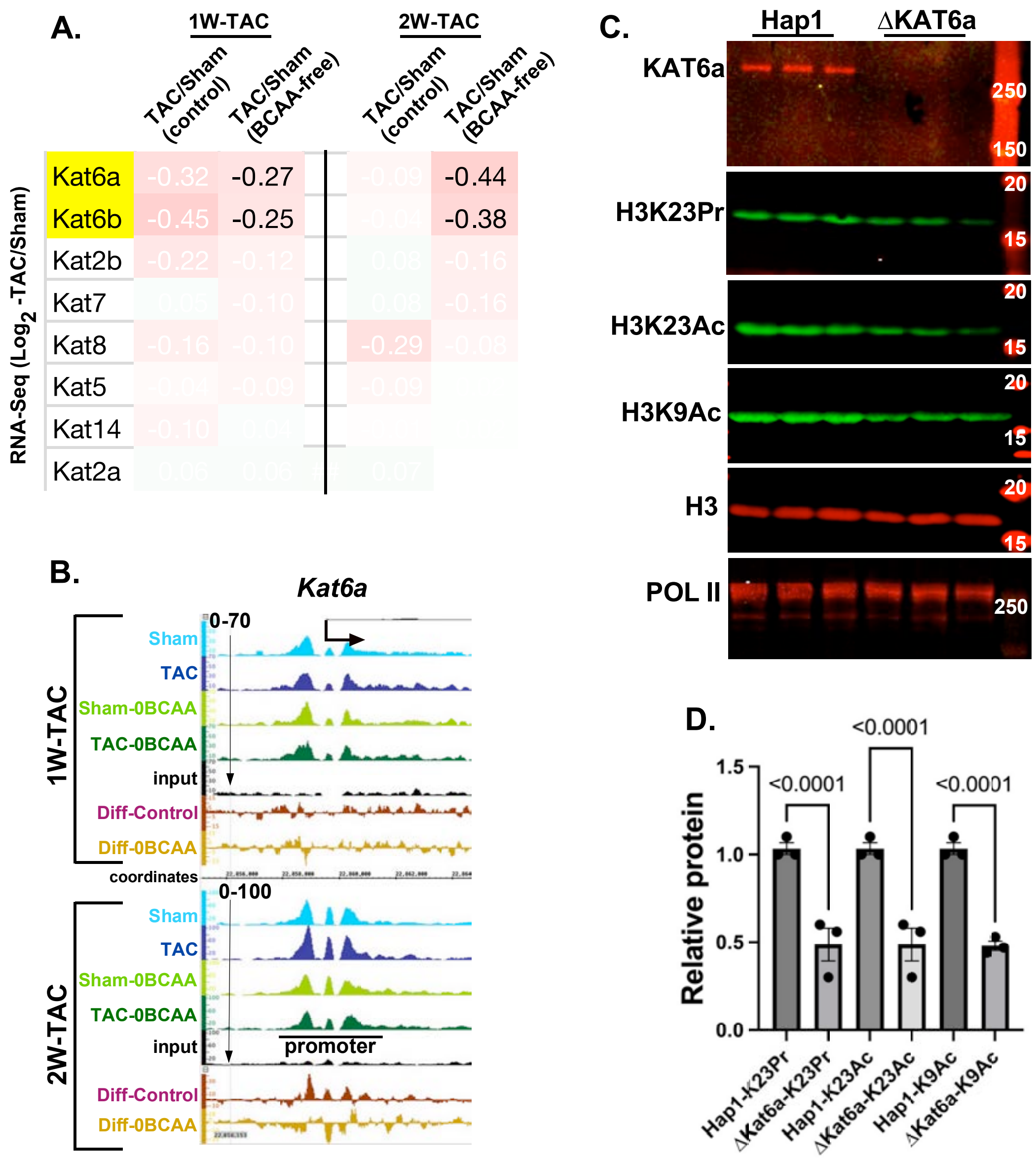

Figure 16S. TAC induce downregulation of Kat6a and Kat6b with a BCAA-free diet. A. Mice were treated as described in figure 2 (in main text). After 1W or 2W post-TAC, the hearts were isolated, RNA extracted and sequenced (n=3, each). The heatmaps show the LFC of TAC/Sham (control) and TAC/Sham (BCAA-free), of the mRNA for the Kat isoforms detect in the heart. The values displayed in black are those with padj of ≤0.05. The color code bar is shown on the right. **B.** Integrated genome browser images showing H3K23Pr ChIP-sequence Tags from the 1W and 2W sham and TAC hearts of mice on the different diets, aligned across *Kat6a* gene coordinates. The labels on the left of each track indicate the surgical and diet conditions applied in mice; Sham and TAC surgeries with the BCAA-control diet (blue tracks), with the BCAA-free diets (green tracks); the differences (Diff) in the H3K23Pr sequence tags of TAC minus sham are shown in separate tracks for the control diet (Diff-Control, brown track) and BCAA-free diet (Diff-0BCAA, gold track). **C.** Hap1 and HapΔKat6a cells were cultured in DMEM with 10% FBS. Cells were then harvested and chromatin- bound proteins were extracted and analyzed by Western bloting for the antibodies listed on the left. **D.** Western blot signals were quantitated and normalized to that of H3 (n=3). The relative values were calculated after adjusting one of the Hap1 signals to 1. Error bars represent SD. The results were analyzed by one-way Anova, comparing the means of Hap1 vs ΔKat6a for each of the H3 modifications (Šídák's multiple comparisons test). The p values are listed above the brackets encompassing the bars.

Figure 16S

Supplementary table 1

A12450K, A19121601, A21123001-02, and A22041404-05

Rodent Diet w/ 10 kcal% Fat

and Same With No Added BCAAs and Sodium Propionate

OpenSource DIETS®

Formulated by
Research Diets, Inc
Dushyant Kshatriya
4/14/2022

Product #	A12450K	A21123001	A22041404	A19121601	A21123002	A22041405
Ingredient	gm	gm	gm	gm	gm	gm
Casein, 80 Mesh	0	0	0	0	0	0
L-Cystine	4.2	4.2	4.2	4.2	4.2	4.2
L-Isoleucine	7.6	7.6	7.6	0	0	0
L-Leucine	15.8	15.8	15.8	0	0	0
L-Lysine	13.2	13.2	13.2	13.2	13.2	13.2
L-Methionine	5.1	5.1	5.1	5.1	5.1	5.1
L-Phenylalanine	8.4	8.4	8.4	8.4	8.4	8.4
L-Threonine	7.2	7.2	7.2	7.2	7.2	7.2
L-Tryptophan	2.1	2.1	2.1	2.1	2.1	2.1
L-Valine	9.3	9.3	9.3	0	0	0
L-Histidine	4.6	4.6	4.6	4.6	4.6	4.6
L-Alanine	5.1	5.1	5.1	5.1	5.1	5.1
L-Arginine	6.0	6.0	6.0	6.0	6.0	6.0
L-Aspartic Acid	12.1	12.1	12.1	12.1	12.1	12.1
L-Glutamic Acid	38.2	38.2	38.2	38.2	38.2	38.2
Glycine	3.0	3.0	3.0	3.0	3.0	3.0
L-Proline	17.8	17.8	17.8	17.8	17.8	17.8
L-Serine	10.0	10.0	10.0	10.0	10.0	10.0
L-Tyrosine	9.2	9.2	9.2	9.2	9.2	9.2
Corn Starch	550	550	550	**582.7**	**582.7**	**582.7**
Maltodextrin 10	150	150	150	150	150	150
Sucrose	0	0	0	0	0	0
Cellulose, BW200	50	50	50	50	50	50
Soybean Oil	25	25	25	25	25	25
Lard	20	20	20	20	20	20
Mineral Mix S10026	10	10	10	10	10	10
DiCalcium Phosphate	13	13	13	13	13	13
Calcium Carbonate	5.5	5.5	5.5	5.5	5.5	5.5
Potassium Citrate, 1 H2O	16.5	16.5	16.5	16.5	16.5	16.5
Sodium BiCarbonate	7.5	7.5	7.5	7.5	7.5	7.5
Vitamin Mix V10001	10	10	10	10	10	10
Choline Bitartrate	2	2	2	2	2	2
Sodium Propionate	**0**	**1.04**	**10.49**	**0**	**1.04**	**10.49**
FD&C Yellow Dye #5	0	0.05	0	0	0.025	0.025
FD&C Red Dye #40	0.025	0	0	0.05	0	0.025
FD&C Blue Dye #1	0.025	0	0.05	0	0.025	0
**Total**	**1038.45**	**1039.49**	**1048.94**	**1038.45**	**1039.49**	**1048.94**
**Diet #**	**A12450K**	**A21123001**	**Formula 1**	**A19121601**	**A21123002**	**Formula 2**
gm						
Protein	178.9	178.9	178.9	146.2	146.2	146.2
Carbohydrate	710.0	710.0	710.0	742.7	742.7	742.7
Fat	45.0	45.0	45.0	45.0	45.0	45.0
Fiber	50.0	50.0	50.0	50.0	50.0	50.0
gm%						
Protein	17.2	17.2	17.1	14.1	14.1	13.9
Carbohydrate	68.4	68.3	67.7	71.5	71.4	70.8
Fat	4.3	4.3	4.3	4.3	4.3	4.3
Fiber	4.8	4.8	4.8	4.8	4.8	4.8
kcal						
Protein	716	716	716	585	585	585
Carbohydrate	2840	2840	2840	2971	2971	2971
Fat	405	405	405	405	405	405
Total	3961	3961	3961	3961	3961	3961
kcal%						
Protein	18	18	18	15	15	15
Carbohydrate	72	72	72	75	75	75
Fat	10	10	10	10	10	10
Total	100	100	100	100	100	100
kcal / gm	3.8	3.8	3.8	3.8	3.8	3.8
Sodium Propionate (g/kg diet)	0	1.00	10.00	0	1.00	10.00

Research Diets, Inc.
20 Jules Lane
New Brunswick, NJ 08901 USA
info@researchdiets.com

AbdellatifM04.for

RESEARCH DIETS INC.
www.ResearchDiets.com

Supplementary table 2

Thermo Fisher SCIENTIFIC

Thermo Fisher Scientific
Life Sciences Solutions
3175 Staley Road
Grand Island, New York 14072 U.S.A.
(716) 774-6726

RUTGERS THE STATE UNIV OF NEW JERSEY

DMEM Custom Formulation, SKU ME20061L1

Components	Molecular Weight	Concentration (mg/L)	mM
**Amino Acids**			
Glycine	75.0	30.0	0.4
L-Arginine hydrochloride	211.0	84.0	0.39810428
L-Cystine 2HCl	313.0	63.0	0.20127796
L-Glutamine	146.0	584.0	4
L-Histidine hydrochloride-H2O	210.0	42.0	0.2
L-Lysine hydrochloride	183.0	146.0	0.7978142
L-Methionine	149.0	30.0	0.20134228
L-Phenylalanine	165.0	66.0	0.4
L-Serine	105.0	42.0	0.4
L-Threonine	119.0	95.0	0.79831934
L-Tryptophan	204.0	16.0	0.078431375
L-Tyrosine disodium salt dihydrate	261.0	104.0	0.39846742
**Vitamins**			
Choline chloride	140.0	4.0	0.028571429
D-Calcium pantothenate	477.0	4.0	0.008385744
Folic Acid	441.0	4.0	0.009070295
Niacinamide	122.0	4.0	0.032786883
Pyridoxine hydrochloride	206.0	4.0	0.019417476
Riboflavin	376.0	0.4	0.0010638298

Thiamine hydrochloride	337.0	4.0	0.011869436
i-Inositol	180.0	7.2	0.04

DMEM Custom Formulation, SKU ME20061L1
(continued)

Components	Molecular Weight	Concentration (mg/L)	mM
**Inorganic Salts**			
Calcium Chloride (CaCl2) (anhyd.)	111.0	200.0	1.8018018
Ferric Nitrate (Fe(NO3)3"9H2O)	404.0	0.1	2.4752476E-04
Magnesium Sulfate (MgSO4) (anhyd.)	120.0	97.67	0.8139166
Potassium Chloride (KCl)	75.0	400.0	5.3333335
Sodium Bicarbonate (NaHCO3)	84.0	3700.0	44.04762
Sodium Chloride (NaCl)	58.0	6400.0	110.344826
Sodium Phosphate monobasic (NaH2PO4-H2O)	138.0	125.0	0.9057971
**Other Components**			
Phenol Red	376.4	15.0	0.039851222

Supplementary table 2. Formula for the BCAA-free DMEM. The BCAA's were added to generate 1x BCAA (0.8 mM of each, Ile, Leu, Val) , 0.1XBCAA (0.08 mM of each, Ile, Leu, and Val), 0.1XIle (0.08 mM Ile, 0.8 mM of each, Leu and Val), 0.1xLeu (0.08 mM Leu, 0.8 mM of each Ile and Val), or 0.1xVal (0.08 mM Val, 0.8 mM of each Ile and Leu), as indicated in the figure legends.

Supplementary table 3 - antibodies

**Antibody**	**Company, catalog #**
anti-H3K23Pr	Abcam, ab241466 for **ChIP-Seq**
anti-H3K23Pr	Active Motif, 61397
anti-H3K18Pr	PTM BIO, PTM-213
anti-H3K56Pr	PTM BIO, PTM-220RM
anti-H3K23Ac	Millipore Sigma, 07-355
anti-Lys-propionyl (K-pr)	PTM BIO, PTM-201
anti-H3	Active Motif, 61475
anti-Bcat2	Millipore Sigma, HPA054091
anti-Bcat1	Cell Signaling Technology, 88785
anti-Col1a1	Cell Signaling Technology, 72026
anti-Ki67	Abcam, ab16667
anti-Alpha smooth muscle actin	Millipore Sigma, A5228
Anti-alpha smooth muscle actin	Cell Signaling Technology, 19245
anti-Alpha-tubulin	Abcam, ab7291
anti-Myh7	Millipore Sigma, M8421
anti-Clic1	Abcam, ab229917
anti-Akt	Millipore Sigma, 07-416
anti-Myoz2	Millipore Sigma, SAB1401710
anti-Ankrd1	Santa Cruz Biotechnology, sc-30181
anti-Tnnt2	Millipore Sigma, SAB2108239
anti-Vdac1	Genscript, A01419
anti-Vdac2	Millipore Sigma, AV35113
anti-RNA polymerase II	Active Motif, 102660
anti-Timm23	Millipore Sigma, HPA031408

Full unedited gels for Figure 1A

ΔBCAT2 +/- BCAAs + Glucose: H3K23Pr (Active Motif 61397) 1:2000 Low Exposure

- 3ug of protein loaded per well
- Expected size of H3K23Ac: ~17 kDa
  - Well 1: Hap-1 -BCAAs +G 1
  - Well 2: Hap-1 -BCAAs +G 2
  - Well 3: Hap-1 -BCAAs +G 3
  - Well 4: Hap-1 +BCAAs +G 1
  - Well 5: Hap-1 +BCAAs +G 2
  - Well 6: Hap-1 +BCAAs +G 3
  - Well 7: ΔBCAT2 -BCAAs +G 1
  - Well 8: ΔBCAT2 -BCAAs +G 2
  - Well 9: ΔBCAT2 -BCAAs +G 3
  - Well 10: ΔBCAT2 +BCAAs +G 1
  - Well 11: ΔBCAT2 +BCAAs +G 2
  - Well 12: ΔBCAT2 +BCAAs +G 3
  - Well 13: Ladder

  - **G=glucose**

Full unedited gels for **Figure 1A**

Histone extracts

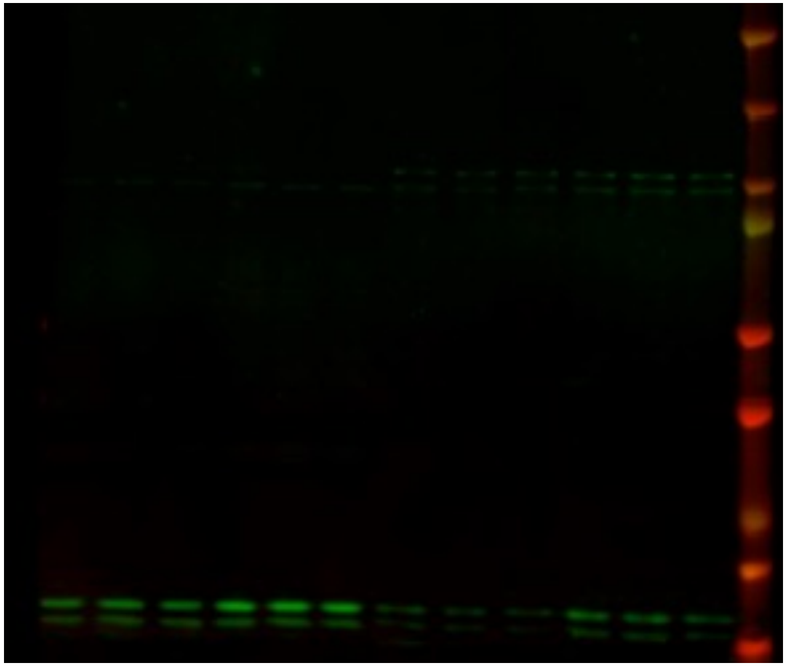

ΔBCAT2 +/- BCAAs + Glucose: H3K23Ac (Millipore 07-355-S) 1:1000 Low Exposure

- 3ug of protein loaded per well
- Expected size of H3K23Ac: ~17 kDa
  - Well 1: Hap-1 -BCAAs +G 1
  - Well 2: Hap-1 -BCAAs +G 2
  - Well 3: Hap-1 -BCAAs +G 3
  - Well 4: Hap-1 +BCAAs +G 1
  - Well 5: Hap-1 +BCAAs +G 2
  - Well 6: Hap-1 +BCAAs +G 3
  - Well 7: ΔBCAT2 -BCAAs +G 1
  - Well 8: ΔBCAT2 -BCAAs +G 2
  - Well 9: ΔBCAT2 -BCAAs +G 3
  - Well 10: ΔBCAT2 +BCAAs +G 1
  - Well 11: ΔBCAT2 +BCAAs +G 2
  - Well 12: ΔBCAT2 +BCAAs +G 3
  - Well 13: Ladder

  - **G=glucose**

Full unedited gels for **Figure 1A**

Histone extracts

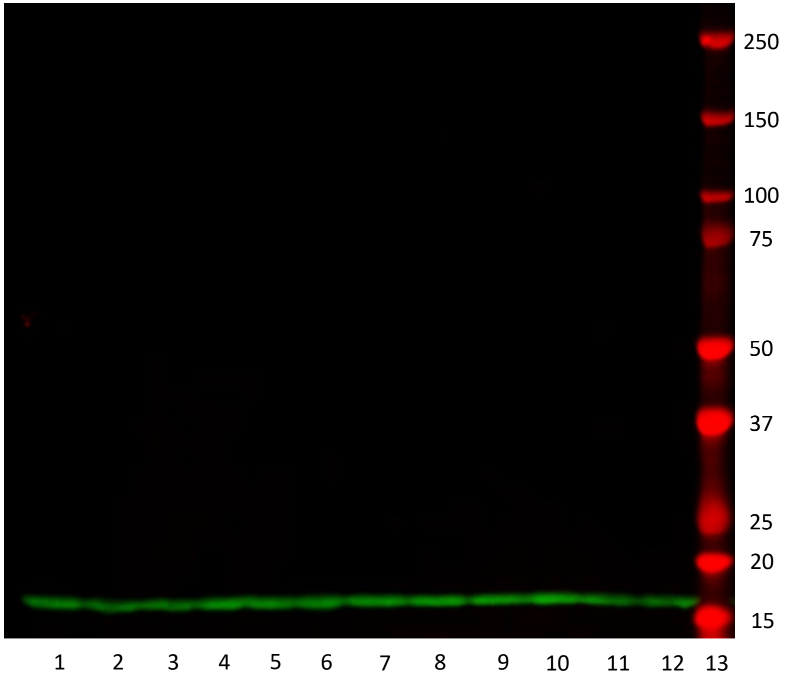

ΔBCAT2 +/- BCAAs + Glucose: H3 (Active Motif 61475) 1:30,000 Low Exposure Reduced Background

- 3ug of protein loaded per well
- Expected size of H3: ~ 17 kDa
    - Well 14: Hap-1 -BCAAs +G 1
    - Well 15: Hap-1 -BCAAs +G 2
    - Well 16: Hap-1 -BCAAs +G 3
    - Well 17: Hap-1 +BCAAs +G 1
    - Well 18: Hap-1 +BCAAs +G 2
    - Well 19: Hap-1 +BCAAs +G 3
    - Well 20: ΔBCAT2 -BCAAs +G 1
    - Well 21: ΔBCAT2 -BCAAs +G 2
    - Well 22: ΔBCAT2 -BCAAs +G 3
    - Well 23: ΔBCAT2 +BCAAs +G 1
    - Well 24: ΔBCAT2 +BCAAs +G 2
    - Well 25: ΔBCAT2 +BCAAs +G 3
    - Well 26: Ladder
    - **G=glucose**

Full unedited gels for Figure 1A
Histone extracts

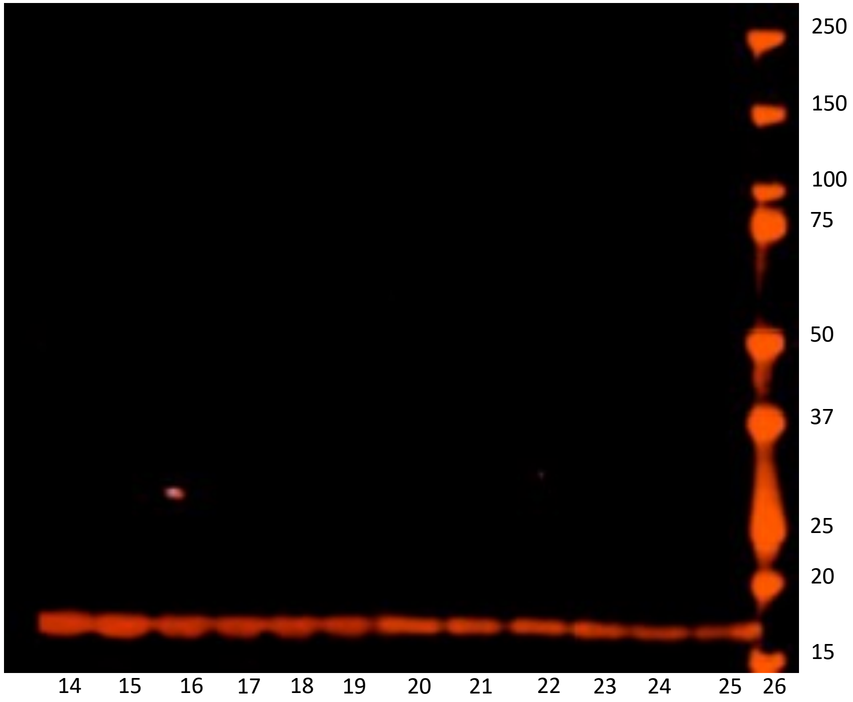

Full unedited gels for Figure 1C

Blot 1 ΔPCCA 0.1x LIV Histones: H3K23Pr (Active Motif 61397) 1:1000

- Expected size: ~17 kDa
- Volume of sample loaded per well determined by protein quantification using Thermo Micro BCA Protein Assay Kit; 3ug of protein
- Well 1: Hap-1 0.1X LIV 1
- Well 2: Hap-1 0.1X LIV 2
- Well 3: Hap-1 0.1X LIV 3
- Well 4: ΔPCCA 0.1X LIV 1
- Well 5: ΔPCCA 0.1X LIV 2
- Well 6: ΔPCCA 0.1X LIV 3
- Wells 7: contains the Ladder; 2.0 µL
- **LIV = Leu, Ile, Val (BCAA)**

Full unedited gel for **Figure 1C**

Histone extracts

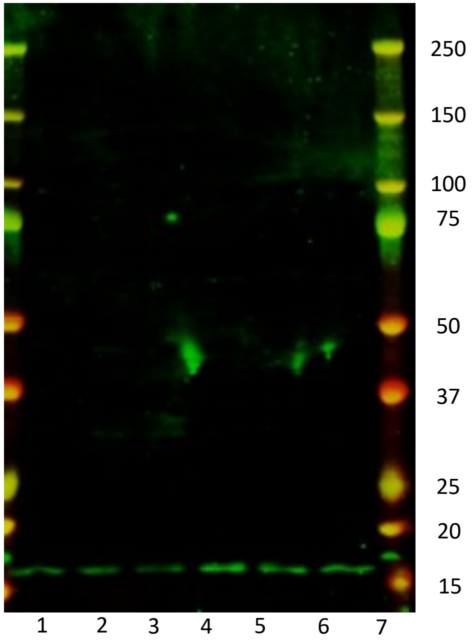

Blot 2 ΔPCCA 0.1X LIV Histones: H3K18Pr (PTM BIO PTM-213) 1:1000 Low Exposure

- Expected size: ~17 kDa
- Volume of sample loaded per well determined by protein quantification using Thermo Micro BCA Protein Assay Kit; 3ug of protein
- Well 1: Hap-1 0.1X LIV 1
- Well 2: Hap-1 0.1X LIV 2
- Well 3: Hap-1 0.1X LIV 3
- Well 4: ΔPCCA 0.1X LIV 1
- Well 5: ΔPCCA 0.1X LIV 2
- Well 6: ΔPCCA 0.1X LIV 3
- Wells 7: contains the Ladder; 2.0 µL
- **LIV = Leu, Ile, Val (BCAA)**

Full unedited gel for Figure 1C

Histone extracts

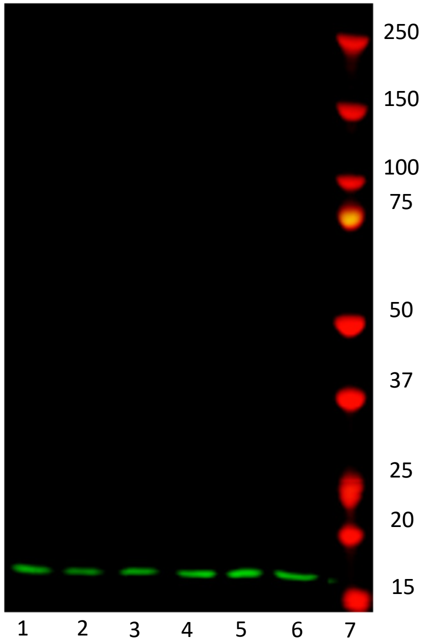

Blot 3 ΔPCCA 0.1X LIV
Histones: H3K56Pr (PTM BIO PTM-220RM) 1:1000
Low Exposure

- Expected size: ~17 kDa
- Volume of sample loaded per well determined by protein quantification using Thermo Micro BCA Protein Assay Kit; 3ug of protein
- Well 1: Hap-1 0.1X LIV 1
- Well 2: Hap-1 0.1X LIV 2
- Well 3: Hap-1 0.1X LIV 3
- Well 4: ΔPCCA 0.1X LIV 1
- Well 5: ΔPCCA 0.1X LIV 2
- Well 6: ΔPCCA 0.1X LIV 3
- Wells 7: contains the Ladder; 2.0 µL
- **LIV = Leu, Ile, Val (BCAA)**

Full unedited gel for Figure 1C

Histone extracts

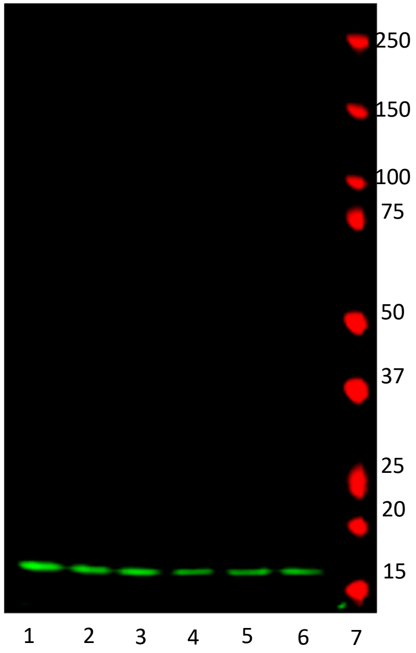

Blot 4 ΔPCCA 0.1x LIV
Histones: H3K23Ac
(Millipore 07-355) 1:1000
Low Exposure Reduced
Background

- Expected size: ~17 kDa
- Volume of sample loaded per well determined by protein quantification using Thermo Micro BCA Protein Assay Kit; 3ug of protein
- Well 1: Hap-1 0.1X LIV 1
- Well 2: Hap-1 0.1X LIV 2
- Well 3: Hap-1 0.1X LIV 3
- Well 4: ΔPCCA 0.1X LIV 1
- Well 5: ΔPCCA 0.1X LIV 2
- Well 6: ΔPCCA 0.1X LIV 3
- Wells 7: contains the Ladder; 2.0 µL
- **LIV = Leu, Ile, Val (BCAA)**

Full unedited gel for Figure 1C

Histone extracts

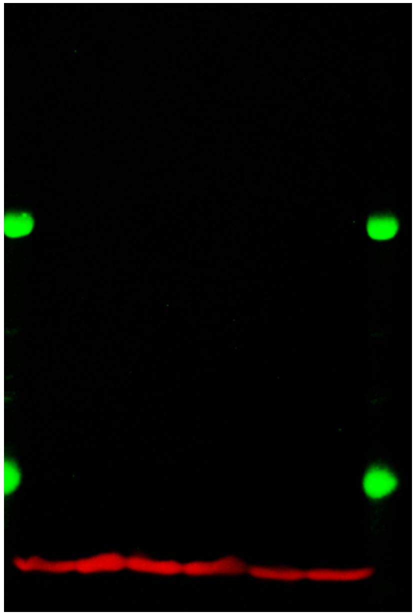

Blot 6 ΔPCCA 0.1X LIV
Histones: H3 (Active Motif 61475) 1:30,000 Low Exposure

- Expected size: ~17 kDa
- Volume of sample loaded per well determined by protein quantification using Thermo Micro BCA Protein Assay Kit; 3ug of protein
- Well 1: Hap-1 0.1X LIV 1
- Well 2: Hap-1 0.1X LIV 2
- Well 3: Hap-1 0.1X LIV 3
- Well 4: ΔPCCA 0.1X LIV 1
- Well 5: ΔPCCA 0.1X LIV 2
- Well 6: ΔPCCA 0.1X LIV 3
- Wells 7: contains the Ladder; 2.0 µL
- **LIV = Leu, Ile, Val (BCAA)**

Full unedited gel for Figure 1C

Histone extracts

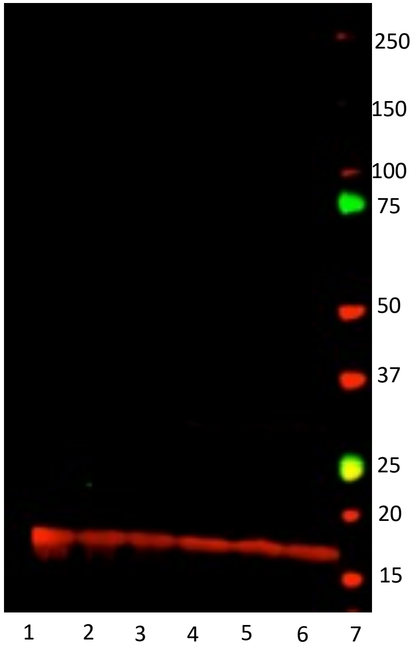

Full unedited gels for Figure 6F

Cardiac Myocytes -BCAAs + Glucose + Sodium Propionate : H3K23Pr (Active Motif 61397) 1:2000

- 3ug of protein loaded per well
- Expected size of H3K23Pr: ~ 17 kDa
  - Well 1: -BCAAs +Glucose 1
  - Well 2: -BCAAs +Glucose 2
  - Well 3: -BCAAs +Glucose + 0.25mM Sodium Propionate 1
  - Well 4: -BCAAs +Glucose + 0.25mM Sodium Propionate 2
  - Well 5: -BCAAs +Glucose + 0.5mM Sodium Propionate 1
  - Well 6: -BCAAs +Glucose + 0.5mM Sodium Propionate 2
  - Well 7: -BCAAs +Glucose + 0.75mM Sodium Propionate 1
  - Well 8: -BCAAs +Glucose + 0.75mM Sodium Propionate 2
  - Well 9: -BCAAs +Glucose + 1.0mM Sodium Propionate 1
  - Well 10: -BCAAs +Glucose + 1.0mM Sodium Propionate 2
  - Well 11 -BCAAs +Glucose + 1.5mM Sodium Propionate 1
  - Well 12: -BCAAs +Glucose + 1.5mM Sodium Propionate 2
  - Well 13: Ladder

Full unedited gel for Figure 6F

Histone extracts

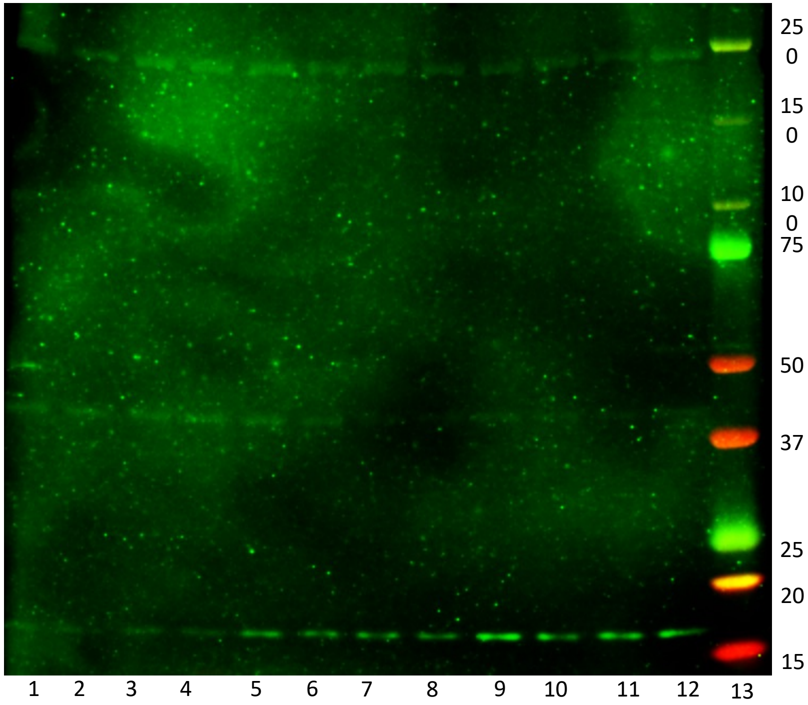

Cardiac Myocytes -BCAAs + Glucose + Sodium Propionate: KPR (PTM BIO PTM-201) 1:1000 Low Exposure

- 3ug of protein loaded per well
- Expected size of KPR: 15-17 kDa
  - Well 1: -BCAAs +Glucose 1
  - Well 2: -BCAAs +Glucose 2
  - Well 3: -BCAAs +Glucose + 0.25mM Sodium Propionate 1
  - Well 4: -BCAAs +Glucose + 0.25mM Sodium Propionate 2
  - Well 5: -BCAAs +Glucose + 0.5mM Sodium Propionate 1
  - Well 6: -BCAAs +Glucose + 0.5mM Sodium Propionate 2
  - Well 7: -BCAAs +Glucose + 0.75mM Sodium Propionate 1
  - Well 8: -BCAAs +Glucose + 0.75mM Sodium Propionate 2
  - Well 9: -BCAAs +Glucose + 1.0mM Sodium Propionate 1
  - Well 10: -BCAAs +Glucose + 1.0mM Sodium Propionate 2
  - Well 11 -BCAAs +Glucose + 1.5mM Sodium Propionate 1
  - Well 12: -BCAAs +Glucose + 1.5mM Sodium Propionate 2
  - Well 13: Ladder

Full unedited gel for Figure 6F

Histone extracts

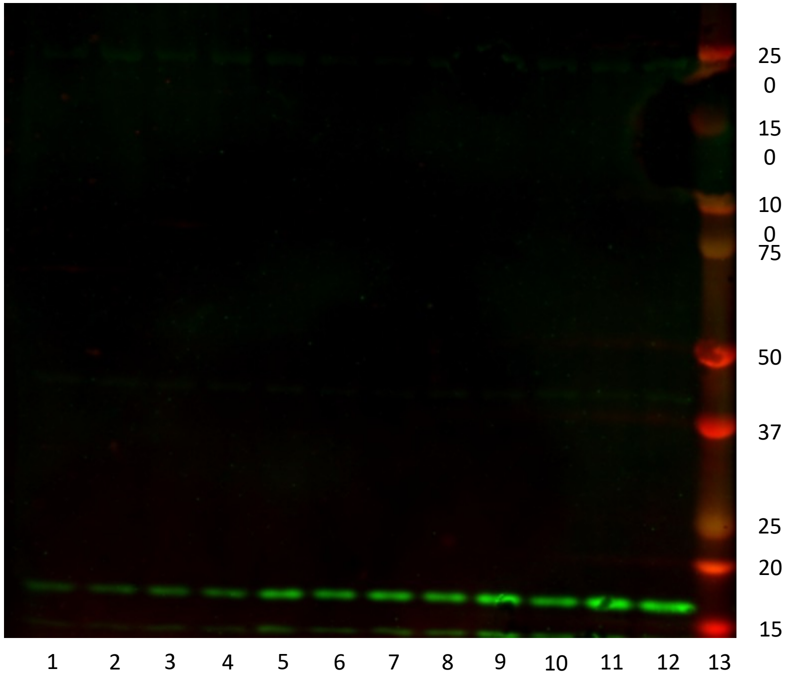

Cardiac Myocytes -BCAAs + Glucose + Sodium Propionate: H3K23Ac (Millipore 07-355-S) 1:2000 Low Exposure

- 3ug of protein loaded per well
- Expected size of H3K23Ac: ~17 kDa
  - Well 1: -BCAAs +Glucose 1
  - Well 2: -BCAAs +Glucose 2
  - Well 3: -BCAAs +Glucose + 0.25mM Sodium Propionate 1
  - Well 4: -BCAAs +Glucose + 0.25mM Sodium Propionate 2
  - Well 5: -BCAAs +Glucose + 0.5mM Sodium Propionate 1
  - Well 6: -BCAAs +Glucose + 0.5mM Sodium Propionate 2
  - Well 7: -BCAAs +Glucose + 0.75mM Sodium Propionate 1
  - Well 8: -BCAAs +Glucose + 0.75mM Sodium Propionate 2
  - Well 9: -BCAAs +Glucose + 1.0mM Sodium Propionate 1
  - Well 10: -BCAAs +Glucose + 1.0mM Sodium Propionate 2
  - Well 11 -BCAAs +Glucose + 1.5mM Sodium Propionate 1
  - Well 12: -BCAAs +Glucose + 1.5mM Sodium Propionate 2
  - Well 13: Ladder

Full unedited gel for Figure 6F
Histone extracts

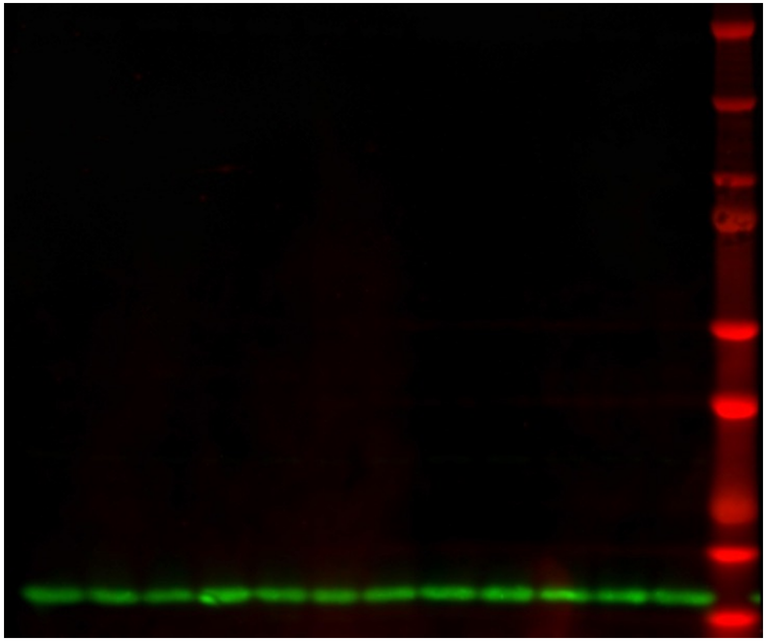

Full unedited gels for Figure 7A

Heart Tissue +/-BCAAs: Col 1A1 (Cell Signaling Technology 72026S) 1:1000

- Expected size: ~138-220 kDa
- Wells 4 through 8 contain all fractions for 1w-Sham-BCAA-control
  - The fractions are organized : cytoplasmic bound proteins, membrane bound proteins, nuclear bound proteins, chromatin bound, and cytoskeletal bound.
- Wells 9 through 13 contain all fractions for 1w-TAC-BCAA-control
  - The fractions are organized: cytoplasmic bound proteins, membrane bound proteins, nuclear bound proteins, chromatin bound, and cytoskeletal bound.
- Wells 14 through 18 contain all fractions for 1w-Sham-BCAA-free
  - The fractions are organized: cytoplasmic bound proteins, membrane bound proteins, nuclear bound proteins, chromatin bound, and cytoskeletal bound.
- Wells 19 through 23 contain all fractions for 1w-TAC-BCAA-free
  - The fractions are organized: cytoplasmic bound proteins, membrane bound proteins, nuclear bound proteins, chromatin bound, and cytoskeletal bound.
- Wells 3 and 24 contain the Ladder; 2.0 µL

Full unedited gel for **Figure 7A**

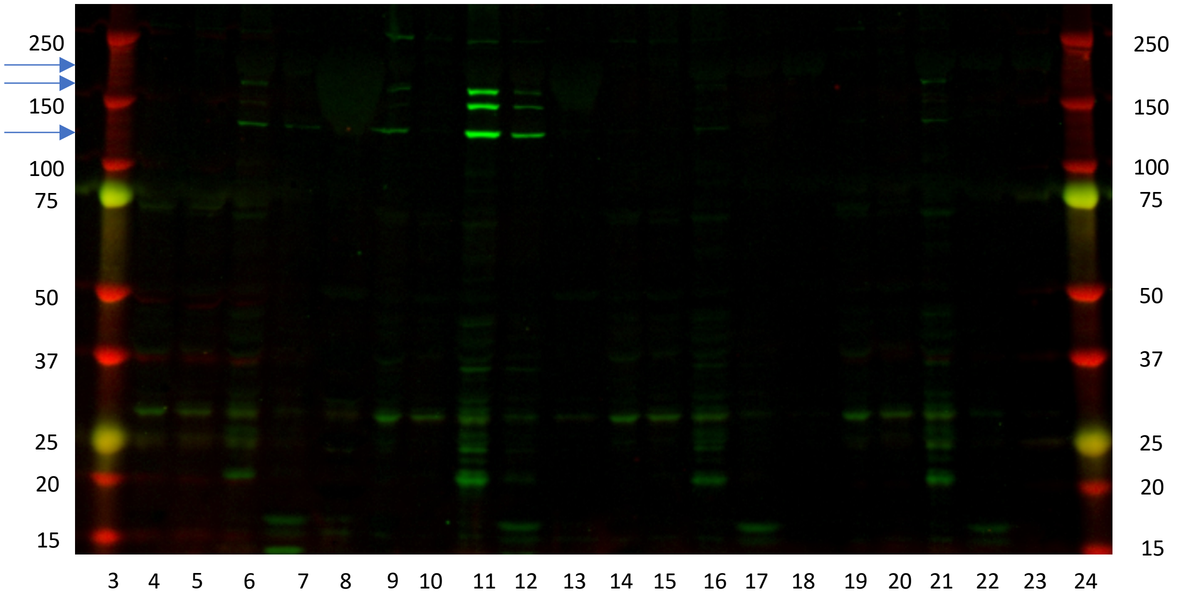

Heart Tissue +/-BCAAs: Beta MHC (Sigma M8421) 1:1000

- Expected size: ~200 kDa
- Wells 4 through 8 contain all fractions for 1w-Sham- BCAAs Control
  - The fractions are organized : cytoplasmic bound proteins, membrane bound proteins, nuclear bound proteins, chromatin bound, and cytoskeletal bound.
- Wells 9 through 13 contain all fractions for 1w-TAC- BCAAs Control
  - The fractions are organized: cytoplasmic bound proteins, membrane bound proteins, nuclear bound proteins, chromatin bound, and cytoskeletal bound.
- Wells 14 through 18 contain all fractions for 1w-Sham-BCAA-free
  - The fractions are organized: cytoplasmic bound proteins, membrane bound proteins, nuclear bound proteins, chromatin bound, and cytoskeletal bound.
- Wells 19 through 23 contain all fractions for 1w-TAC-BCAA-free
  - The fractions are organized: cytoplasmic bound proteins, membrane bound proteins, nuclear bound proteins, chromatin bound, and cytoskeletal bound.
- Wells 3 and 24 contain the Ladder; 2.0 µL

Full unedited gel for **Figure 7A**

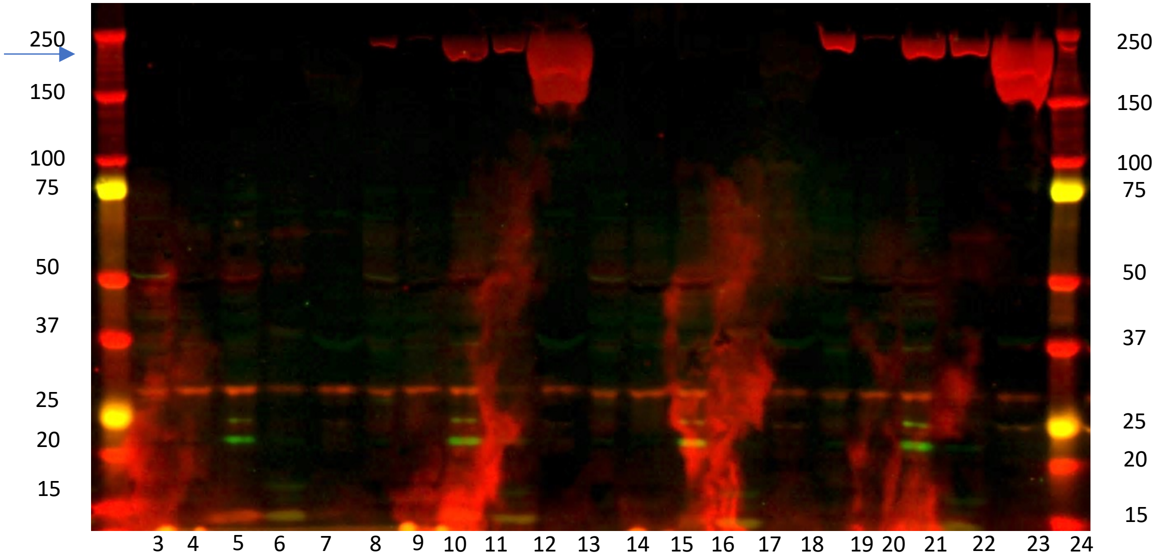

Heart Tissue +/-BCAAs: VDAC1 (Genescript A01419) 1:1000 + RNA Pol II (Active Motif 102660) 1:2000

- Expected size of VDAC1: ~34 kDa
- Expected size of RNA Pol II: ~ 240 kDa
- Wells 4 through 8 contain all fractions for 1w-Sham-BCAA-control
  - The fractions are organized : cytoplasmic bound proteins, membrane bound proteins, nuclear bound proteins, chromatin bound, and cytoskeletal bound.
- Wells 9 through 13 contain all fractions for 1w-TAC-BCAA-control
  - The fractions are organized: cytoplasmic bound proteins, membrane bound proteins, nuclear bound proteins, chromatin bound, and cytoskeletal bound.
- Wells 14 through 18 contain all fractions for 1w-Sham-BCAA-free
  - The fractions are organized: cytoplasmic bound proteins, membrane bound proteins, nuclear bound proteins, chromatin bound, and cytoskeletal bound.
- Wells 19 through 23 contain all fractions for 1w-TAC-BCAA-free
  - The fractions are organized: cytoplasmic bound proteins, membrane bound proteins, nuclear bound proteins, chromatin bound, and cytoskeletal bound.
- Wells 3 and 24 contain the Ladder; 2.0 µL

Full unedited gel for Figure 7A

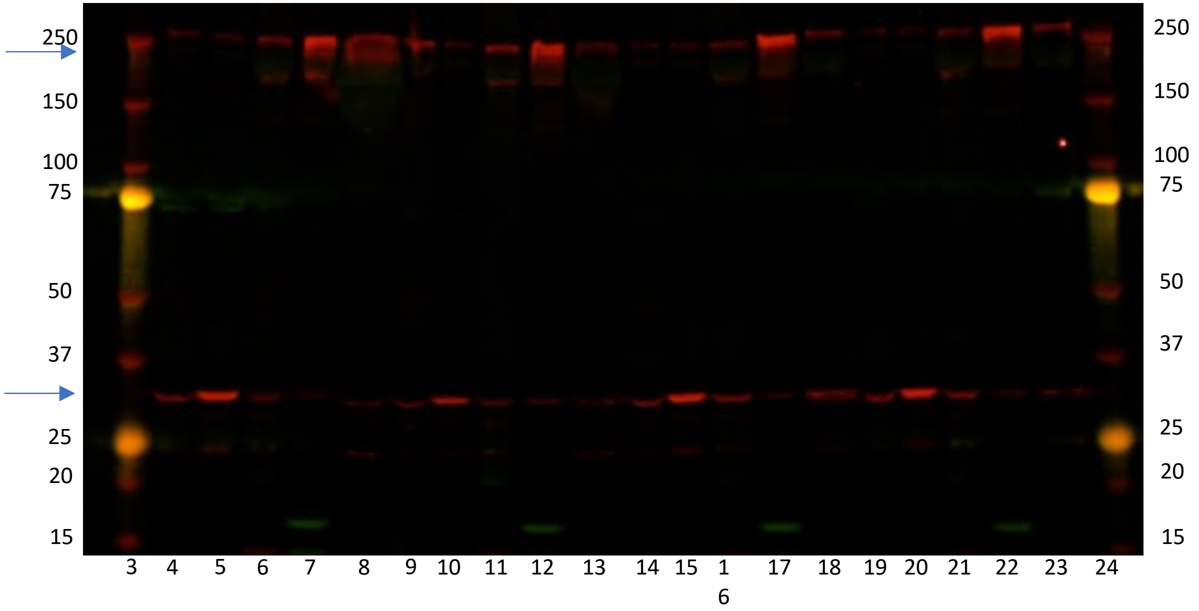

Heart Tissue +/-BCAAs:
CLIC1 (abcam ab229917)
1:1000

- Expected size: ~27 kDa
- Wells 4 through 8 contain all fractions for 1w-Sham-BCAA-control
  - The fractions are organized : cytoplasmic bound proteins, membrane bound proteins, nuclear bound proteins, chromatin bound, and cytoskeletal bound.
- Wells 9 through 13 contain all fractions for 1w-TAC-BCAA-control
  - The fractions are organized: cytoplasmic bound proteins, membrane bound proteins, nuclear bound proteins, chromatin bound, and cytoskeletal bound.
- Wells 14 through 18 contain all fractions for 1w-Sham-BCAA-free
  - The fractions are organized: cytoplasmic bound proteins, membrane bound proteins, nuclear bound proteins, chromatin bound, and cytoskeletal bound.
- Wells 19 through 23 contain all fractions for 1w-TAC-BCAA-free
  - The fractions are organized: cytoplasmic bound proteins, membrane bound proteins, nuclear bound proteins, chromatin bound, and cytoskeletal bound.
- Wells 3 and 24 contain the Ladder; 2.0 µL

Full unedited gel for Figure 7A

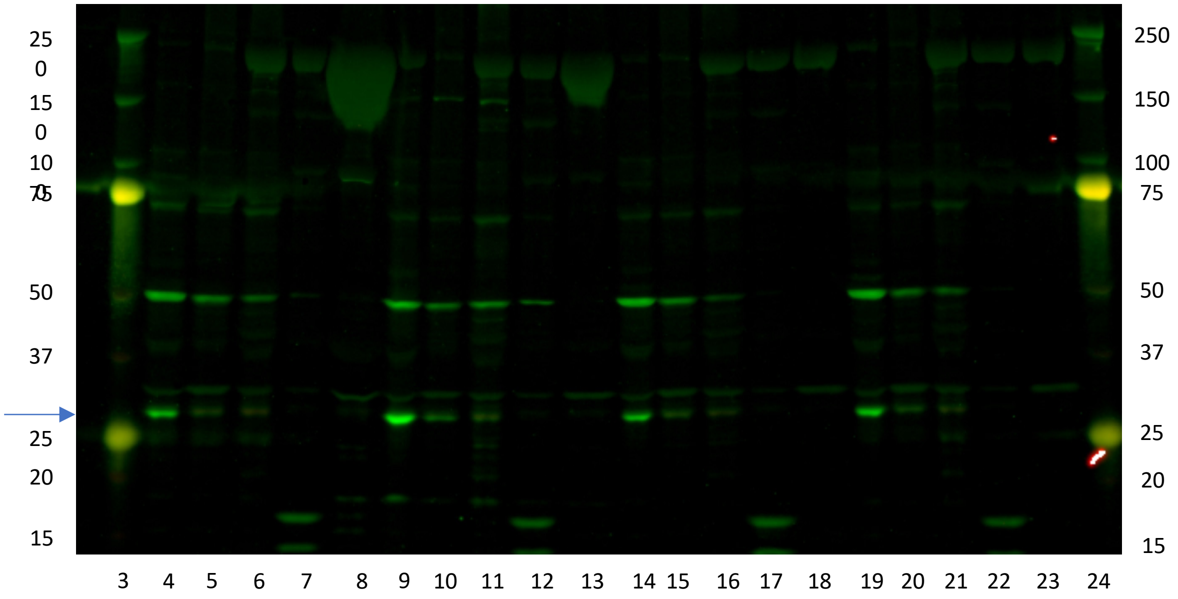

1W Heart Tissue +/-BCAAs: AKT1 (Cell Signaling Technology 9272S) 1:1000 + TNNT2 (Sigma SAB2108239) 1:2000

- Expected size of Akt1: ~60 kDa
- Expected size of TNNT2: ~36 kDa
- Wells 4 through 8 contain all fractions for 1w-Sham- BCAAs Control
    - The fractions are organized : cytoplasmic bound proteins, membrane bound proteins, nuclear bound proteins, chromatin bound, and cytoskeletal bound.
- Wells 9 through 13 contain all fractions for 1w-TAC-BCAAs Control
    - The fractions are organized: cytoplasmic bound proteins, membrane bound proteins, nuclear bound proteins, chromatin bound, and cytoskeletal bound.
- Wells 14 through 18 contain all fractions for 1w-Sham-BCAA-free
    - The fractions are organized: cytoplasmic bound proteins, membrane bound proteins, nuclear bound proteins, chromatin bound, and cytoskeletal bound.
- Wells 19 through 23 contain all fractions for 1w-TAC-BCAA-free
    - The fractions are organized: cytoplasmic bound proteins, membrane bound proteins, nuclear bound proteins, chromatin bound, and cytoskeletal bound.
- Wells 3 and 24 contain the Ladder; 2.0 µL

Full unedited gel for Figure 7A

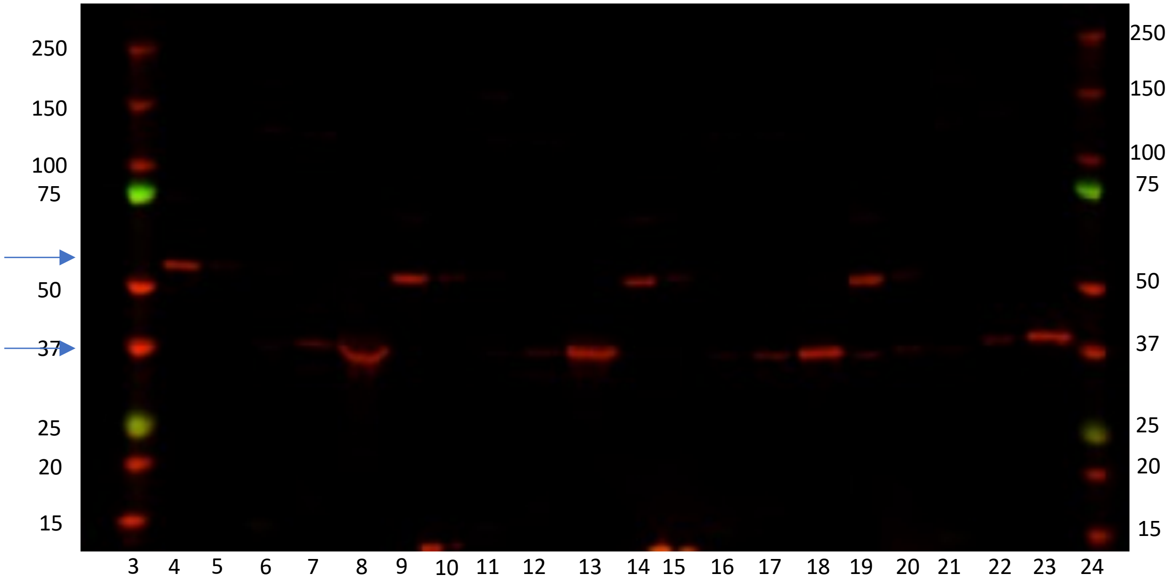

Heart Tissue +/-BCAAs: ANKRD1 (Santa Cruz Biotechnology) 1:1000

- Expected size: ~36 kDa
- Wells 4 through 8 contain all fractions for 1w-Sham- BCAAs Control
    - The fractions are organized : cytoplasmic bound proteins, membrane bound proteins, nuclear bound proteins, chromatin bound, and cytoskeletal bound.
- Wells 9 through 13 contain all fractions for 1w-TAC- BCAAs Control
    - The fractions are organized: cytoplasmic bound proteins, membrane bound proteins, nuclear bound proteins, chromatin bound, and cytoskeletal bound.
- Wells 14 through 18 contain all fractions for 1w-Sham-BCAA-free
    - The fractions are organized: cytoplasmic bound proteins, membrane bound proteins, nuclear bound proteins, chromatin bound, and cytoskeletal bound.
- Wells 19 through 23 contain all fractions for 1w-TAC-BCAA-free
    - The fractions are organized: cytoplasmic bound proteins, membrane bound proteins, nuclear bound proteins, chromatin bound, and cytoskeletal bound.
- Wells 3 and 24 contain the Ladder; 2.0 µL

Full unedited gel for Figure 7A

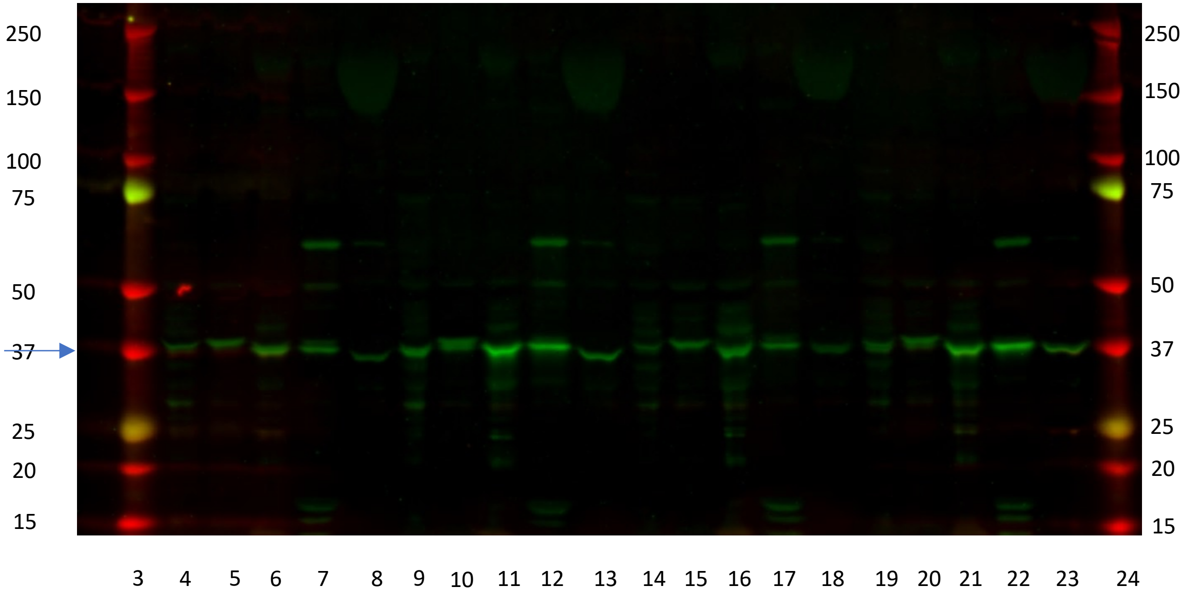

1W Heart Tissue +/- BCAA:
H3K23Pr (Abcam, ab241466) +
~~HDAC9 (abcam ab109446) 1:500~~
(not included in figures)

- Expected size of H3K23Pr: ~17 kDa
- Expected size of HDAC9: ~111 kDa
- Wells 4 through 8 contain all fractions for 1w-Sham- BCAAs Control
    - The fractions are organized : cytoplasmic bound proteins, membrane bound proteins, nuclear bound proteins, chromatin bound, and cytoskeletal bound.
- Wells 9 through 13 contain all fractions for 1w-TAC-BCAAs Control
    - The fractions are organized: cytoplasmic bound proteins, membrane bound proteins, nuclear bound proteins, chromatin bound, and cytoskeletal bound.
- Wells 14 through 18 contain all fractions for 1w-Sham-BCAA-free
    - The fractions are organized: cytoplasmic bound proteins, membrane bound proteins, nuclear bound proteins, chromatin bound, and cytoskeletal bound.
- Wells 19 through 23 contain all fractions for 1w-TAC-BCAA-free
    - The fractions are organized: cytoplasmic bound proteins, membrane bound proteins, nuclear bound proteins, chromatin bound, and cytoskeletal bound.
- Wells 3 and 24 contain the Ladder; 2.0 µL

Full unedited gel for **Figure 7A**

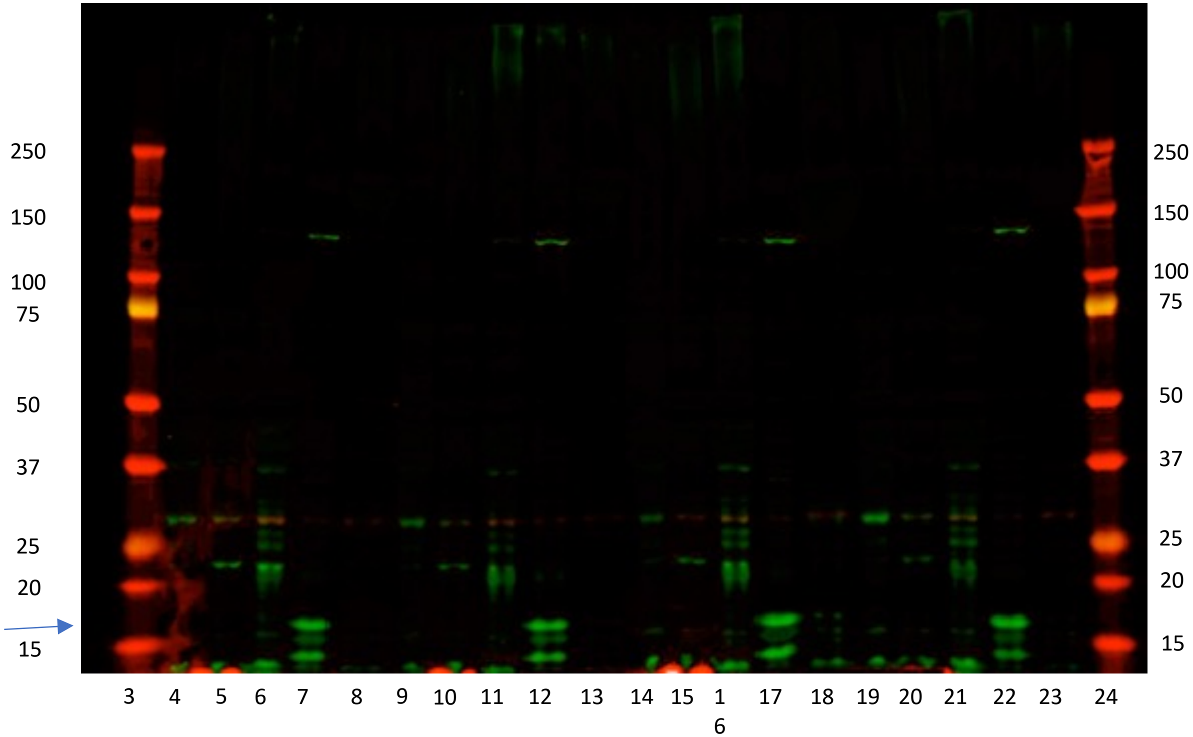

Blot 1 Heart Tissue +/- BCAAs: H3K23Ac (Millipore, 07-355) 1:10,000, ~~Col VI (abcam ab199720) 1:1000~~(not included in figure)

- Expected size: H3K23ac ~17 Kd
- Expected size: Col VI ~138-220 kDa
- Wells 4 through 8 contain all fractions for 1w-Sham- BCAAs Control
  - The fractions are organized : cytoplasmic bound proteins, membrane bound proteins, nuclear bound proteins, chromatin bound, and cytoskeletal bound.
- Wells 9 through 13 contain all fractions for 1w-TAC-BCAAs Control
  - The fractions are organized: cytoplasmic bound proteins, membrane bound proteins, nuclear bound proteins, chromatin bound, and cytoskeletal bound.
- Wells 14 through 18 contain all fractions for 1w-Sham-BCAA-free
  - The fractions are organized: cytoplasmic bound proteins, membrane bound proteins, nuclear bound proteins, chromatin bound, and cytoskeletal bound.
- Wells 19 through 23 contain all fractions for 1w-TAC-BCAA-free
  - The fractions are organized: cytoplasmic bound proteins, membrane bound proteins, nuclear bound proteins, chromatin bound, and cytoskeletal bound.
- Wells 3 and 24 contain the Ladder; 2.0 µL

Full unedited gel for Figure 7A

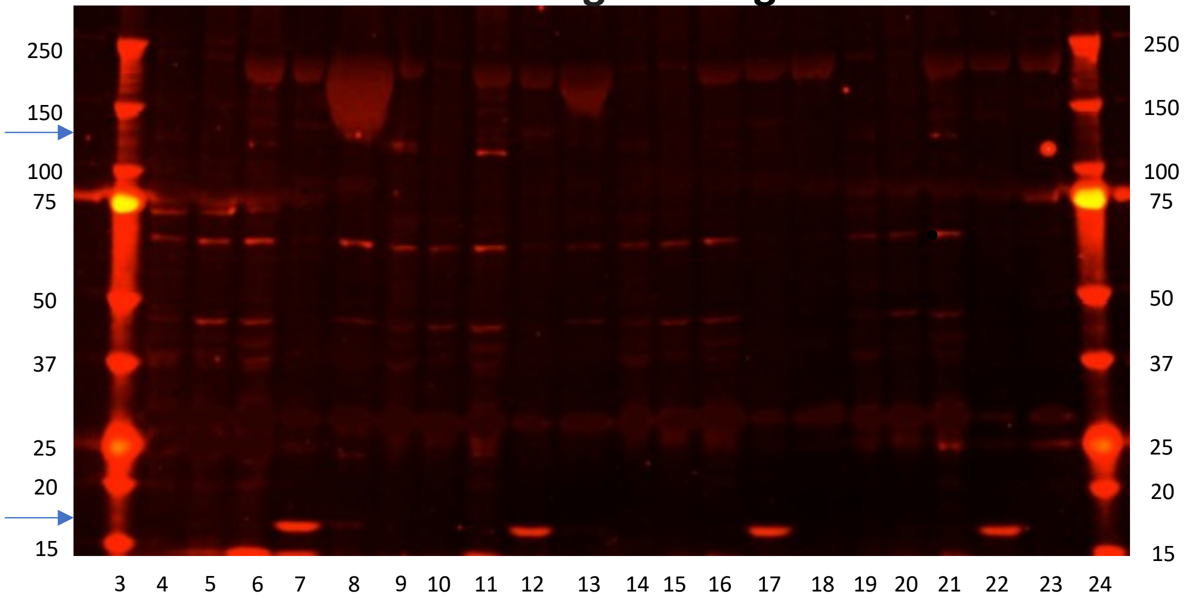

Blot 3 Heart Tissue +/- BCAAs: BCAT2 (Sigma HPA054091) 1:1000

- Expected size: ~39 kDa
- Wells 4 through 8 contain all fractions for 1w-Sham- BCAAs Control
  - The fractions are organized : cytoplasmic bound proteins, membrane bound proteins, nuclear bound proteins, chromatin bound, and cytoskeletal bound.
- Wells 9 through 13 contain all fractions for 1w-TAC-BCAAs Control
  - The fractions are organized: cytoplasmic bound proteins, membrane bound proteins, nuclear bound proteins, chromatin bound, and cytoskeletal bound.
- Wells 14 through 18 contain all fractions for 1w-Sham-BCAA-free
  - The fractions are organized: cytoplasmic bound proteins, membrane bound proteins, nuclear bound proteins, chromatin bound, and cytoskeletal bound.
- Wells 19 through 23 contain all fractions for 1w-TAC-BCAA-free
  - The fractions are organized: cytoplasmic bound proteins, membrane bound proteins, nuclear bound proteins, chromatin bound, and cytoskeletal bound.
- Wells 3 and 24 contain the Ladder; 2.0 µL

Full unedited gel for **Figure 7A**

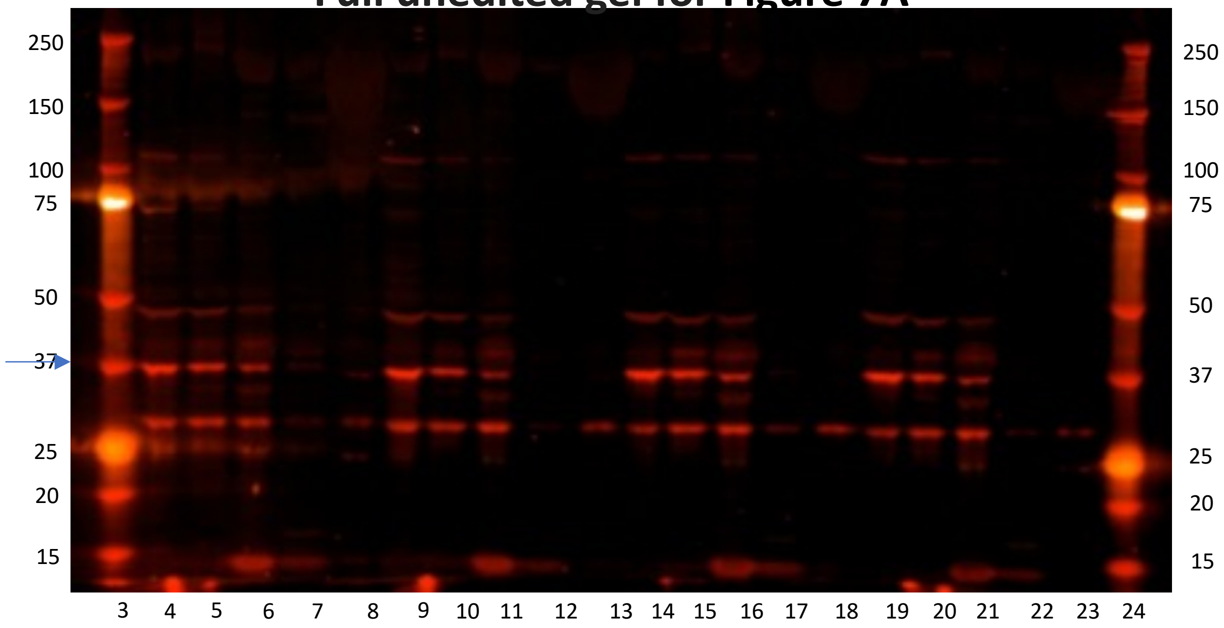

Full unedited gels for Figure 8A

1W Mouse Heart Tissue + 1% propionate +/-BCAAs: COL 1A1 (Cell Signaling Technology 72026S) + ~~Ndufs4 (Sigma HPA003884) 1:1000 –~~ignore- was not included in figure

- Expected size of Col 1A1: ~138-220 kDa
- Expected size of Ndufs4: ~ 17 kDa -ignore
- Wells 4 through 8 contain all fractions for 1w-Sham propionate BCAA-control
    - The fractions are organized : cytoplasmic bound proteins, membrane bound proteins, nuclear bound proteins, chromatin bound, and cytoskeletal bound.
- Wells 9 through 13 contain all fractions for 1w-TAC propionate BCAA-control
    - The fractions are organized: cytoplasmic bound proteins, membrane bound proteins, nuclear bound proteins, chromatin bound, and cytoskeletal bound.
- Wells 14 through 18 contain all fractions for 1w-Sham propionate BCAA-free
    - The fractions are organized: cytoplasmic bound proteins, membrane bound proteins, nuclear bound proteins, chromatin bound, and cytoskeletal bound.
- Wells 19 through 23 contain all fractions for 1w-TAC propionate BCAA-free
    - The fractions are organized: cytoplasmic bound proteins, membrane bound proteins, nuclear bound proteins, chromatin bound, and cytoskeletal bound.
- Wells 3 and 24 contain the Ladder; 2.0 µL

Full unedited gel for Figure 8B

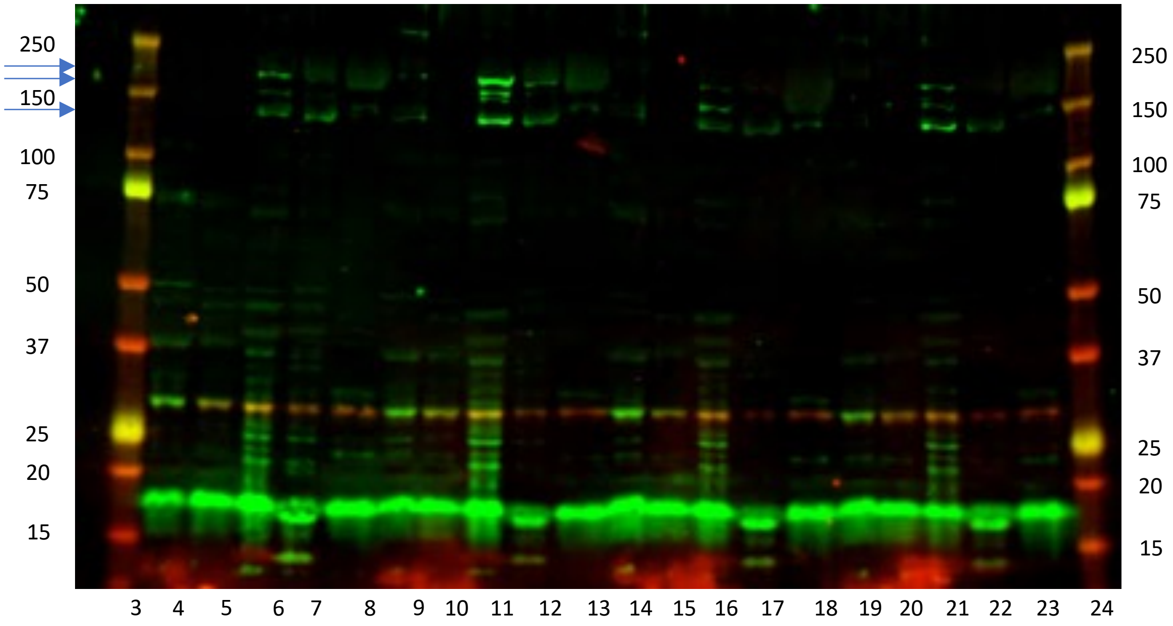

1W Heart Tissue + 1% Propionate: MYH7 (Sigma M8421) 1:1000 + TNNT2 (Sigma SAB2108239) 1:1000

- Expected size of MYH7: ~200 kDa
- Expected size of TNNT2: ~36 kDa
- Wells 4 through 8 contain all fractions for 1w-Sham Prop BCAA-control
  - The fractions are organized : cytoplasmic bound proteins, membrane bound proteins, nuclear bound proteins, chromatin bound, and cytoskeletal bound.
- Wells 9 through 13 contain all fractions for 1w-TAC Prop BCAA-control
  - The fractions are organized: cytoplasmic bound proteins, membrane bound proteins, nuclear bound proteins, chromatin bound, and cytoskeletal bound.
- Wells 14 through 18 contain all fractions for 1w-Sham Prop BCAA-free
  - The fractions are organized: cytoplasmic bound proteins, membrane bound proteins, nuclear bound proteins, chromatin bound, and cytoskeletal bound.
- Wells 19 through 23 contain all fractions for 1w-TAC Prop BCAA-free
  - The fractions are organized: cytoplasmic bound proteins, membrane bound proteins, nuclear bound proteins, chromatin bound, and cytoskeletal bound.
- Wells 3 and 24 contain the Ladder; 2.0 µL

Full unedited gel for **Figure 8B**

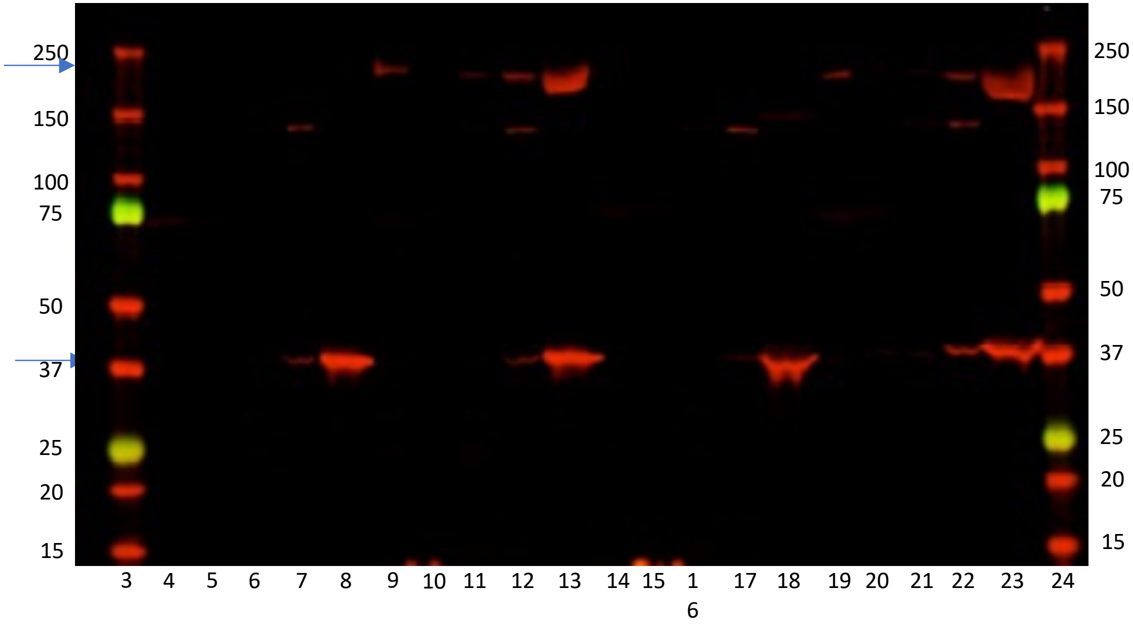

1W Heart Tissue + 1% Propionate: ANKRD1 (Santa Cruz Biotechnology sc-30181) 1:1000 + RNA Pol II (Active Motif 102660) 1:2000

- Expected size of AnKrd1: ~36 kDa
- Expected size of RNA Pol II: ~240 kDa
- Wells 4 through 8 contain all fractions for 1w-Sham Prop BCAA-control
    - The fractions are organized : cytoplasmic bound proteins, membrane bound proteins, nuclear bound proteins, chromatin bound, and cytoskeletal bound.
- Wells 9 through 13 contain all fractions for 1w-TAC Prop BCAA-control
    - The fractions are organized: cytoplasmic bound proteins, membrane bound proteins, nuclear bound proteins, chromatin bound, and cytoskeletal bound.
- Wells 14 through 18 contain all fractions for 1w-Sham Prop BCAA-free
    - The fractions are organized: cytoplasmic bound proteins, membrane bound proteins, nuclear bound proteins, chromatin bound, and cytoskeletal bound.
- *Wells 19 through 23 contain all fractions for 1w-TAC Prop BCAA-free
    - The fractions are organized: cytoplasmic bound proteins, membrane bound proteins, nuclear bound proteins, chromatin bound, and cytoskeletal bound.
- Wells 3 and 24 contain the Ladder; 2.0 µL

Full unedited gel for Figure 8B

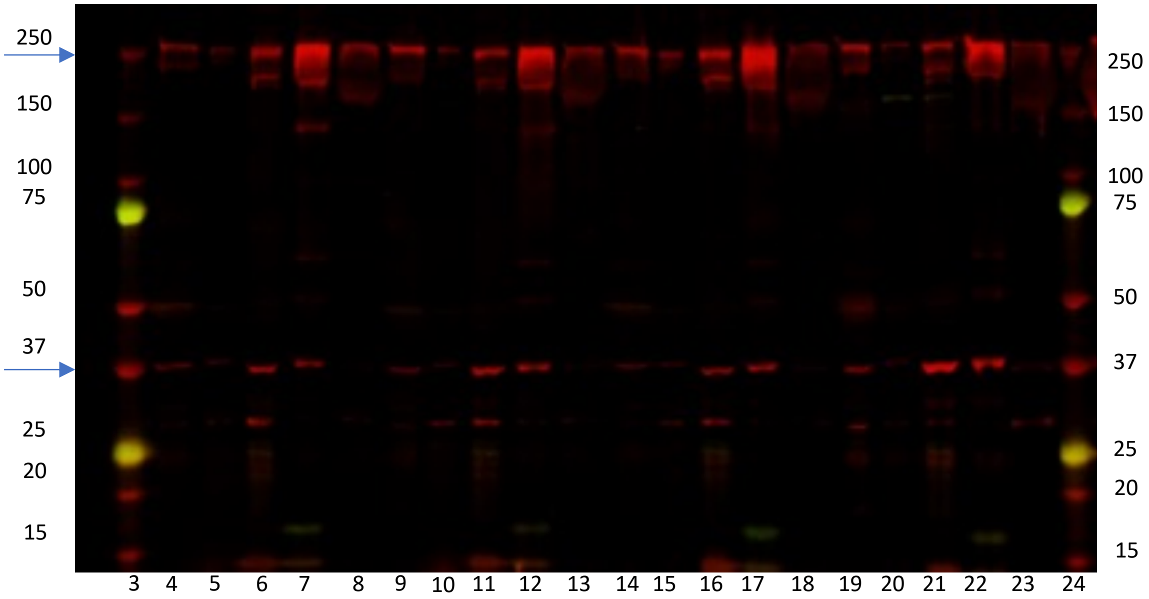

1W Heart Tissue + 1% Propionate: Clic1 (abcam ab229917) 1:1000 Low Exposure

- Expected size of Clic1: ~27 kDa
- Wells 6 through 10 contain all fractions for 1w-Sham Prop BCAA-control
  - The fractions are organized : cytoplasmic bound proteins, membrane bound proteins, nuclear bound proteins, chromatin bound, and cytoskeletal bound.
- Wells 11 through 15 contain all fractions for 1w-TAC Prop BCAA-control
  - The fractions are organized: cytoplasmic bound proteins, membrane bound proteins, nuclear bound proteins, chromatin bound, and cytoskeletal bound.
- Wells 16 through 20 contain all fractions for 1w-Sham Prop BCAA-free
  - The fractions are organized: cytoplasmic bound proteins, membrane bound proteins, nuclear bound proteins, chromatin bound, and cytoskeletal bound.
- Wells 21 through 25 contain all fractions for 1w-TAC Prop BCAA-free
  - The fractions are organized: cytoplasmic bound proteins, membrane bound proteins, nuclear bound proteins, chromatin bound, and cytoskeletal bound.
- Wells 5 and 26 contain the Ladder; 2.0 µL

Full unedited gel for Figure 8B

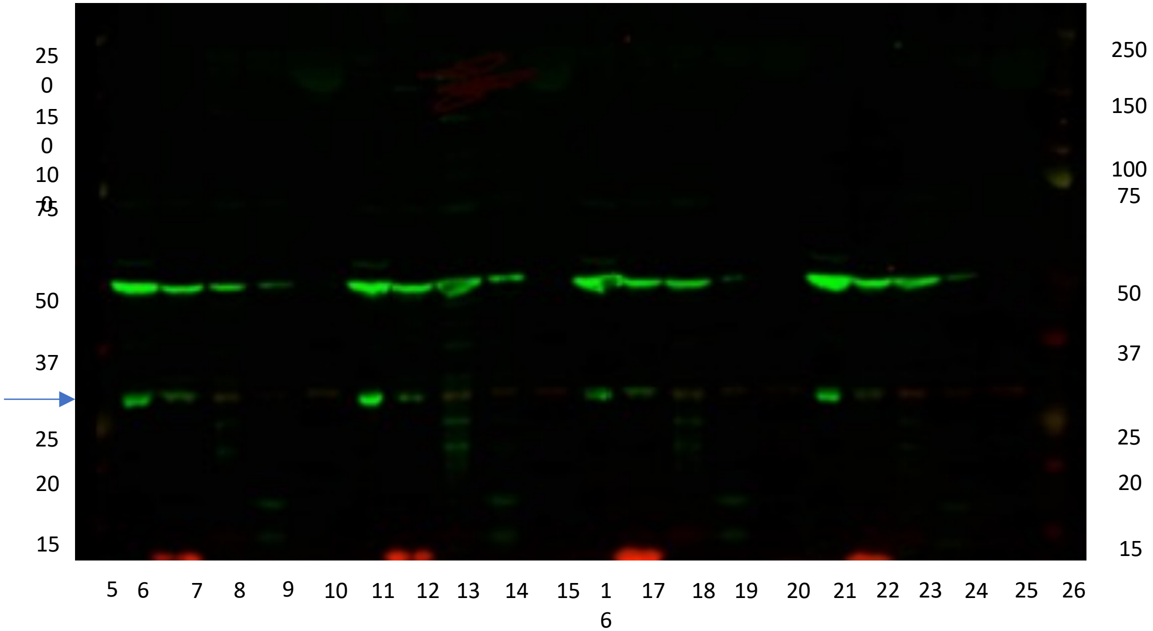

1W Heart Tissue + 1% Propionate: AKT1 (Cell Signaling Technology 9272S) 1:1000 + VDAC1 (Genescript A01914) 1:2000 Low Exposure

- Expected size of Akt1: ~60 kDa
- Expected size of Vdac1: ~34 kDa
- Wells 4 through 8 contain all fractions for 1w-Sham Prop BCAA-control
    - The fractions are organized : cytoplasmic bound proteins, membrane bound proteins, nuclear bound proteins, chromatin bound, and cytoskeletal bound.
- Wells 9 through 13 contain all fractions for 1w-TAC Prop BCAA-control
    - The fractions are organized: cytoplasmic bound proteins, membrane bound proteins, nuclear bound proteins, chromatin bound, and cytoskeletal bound.
- Wells 14 through 18 contain all fractions for 1w-Sham Prop BCAA-free
    - The fractions are organized: cytoplasmic bound proteins, membrane bound proteins, nuclear bound proteins, chromatin bound, and cytoskeletal bound.
- Wells 19 through 23 contain all fractions for 1w-TAC Prop BCAA-free
    - The fractions are organized: cytoplasmic bound proteins, membrane bound proteins, nuclear bound proteins, chromatin bound, and cytoskeletal bound.
- Wells 3 and 24 contain the Ladder; 2.0 µL

Full unedited gel for Figure 8B

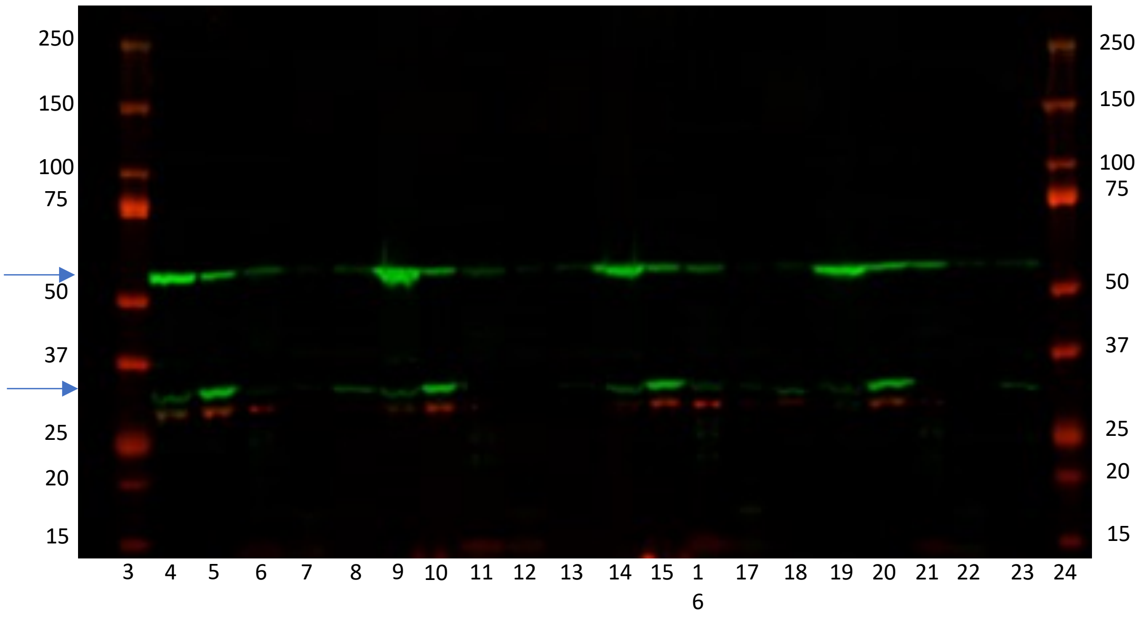

1W Heart Tissue + 1% Propionate: H3K23Pr (Abcam, ab241466) 1:1000

- Expected size: 17~kDa

- Wells 4 through 8 contain all fractions for 1w-Sham- BCAAs Control
    - The fractions are organized : cytoplasmic bound proteins, membrane bound proteins, nuclear bound proteins, chromatin bound, and cytoskeletal bound.
- Wells 9 through 13 contain all fractions for 1w-TAC-BCAAs Control
    - The fractions are organized: cytoplasmic bound proteins, membrane bound proteins, nuclear bound proteins, chromatin bound, and cytoskeletal bound.
- Wells 14 through 18 contain all fractions for 1w-Sham-BCAA-free
    - The fractions are organized: cytoplasmic bound proteins, membrane bound proteins, nuclear bound proteins, chromatin bound, and cytoskeletal bound.
- Wells 19 through 23 contain all fractions for 1w-TAC-BCAA-free
    - The fractions are organized: cytoplasmic bound proteins, membrane bound proteins, nuclear bound proteins, chromatin bound, and cytoskeletal bound.
- Wells 3 and 24 contain the Ladder; 2.0 µL

Full unedited gel for **Figure 8B**

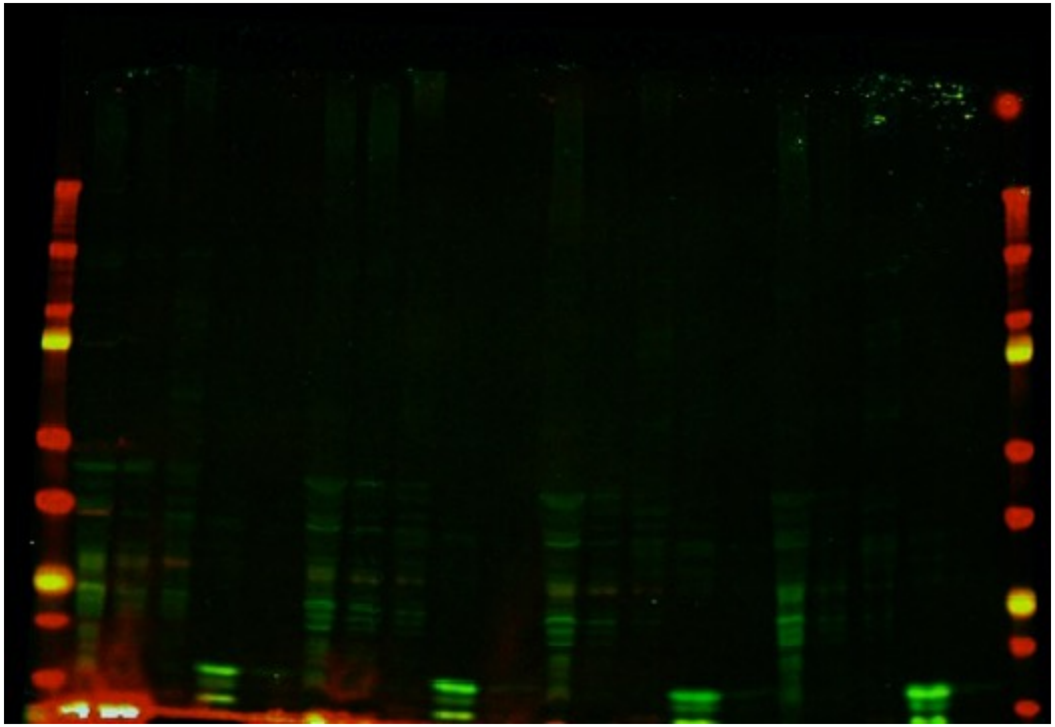

1W Heart Tissue + 1% Propionate : H3K23Ac (Millipore, 07-355) 1:10,000

- Expected size of H3K23Pr: ~17 kDa
- Expected size: 17~kDa
- Wells 4 through 8 contain all fractions for 1w-Sham- BCAAs Control
  - The fractions are organized : cytoplasmic bound proteins, membrane bound proteins, nuclear bound proteins, chromatin bound, and cytoskeletal bound.
- Wells 9 through 13 contain all fractions for 1w-TAC-BCAAs Control
  - The fractions are organized: cytoplasmic bound proteins, membrane bound proteins, nuclear bound proteins, chromatin bound, and cytoskeletal bound.
- Wells 14 through 18 contain all fractions for 1w-Sham-BCAA-free
  - The fractions are organized: cytoplasmic bound proteins, membrane bound proteins, nuclear bound proteins, chromatin bound, and cytoskeletal bound.
- Wells 19 through 23 contain all fractions for 1w-TAC-BCAA-free
  - The fractions are organized: cytoplasmic bound proteins, membrane bound proteins, nuclear bound proteins, chromatin bound, and cytoskeletal bound.
- Wells 3 and 24 contain the Ladder; 2.0 µL

Full unedited gel for **Figure 8B**

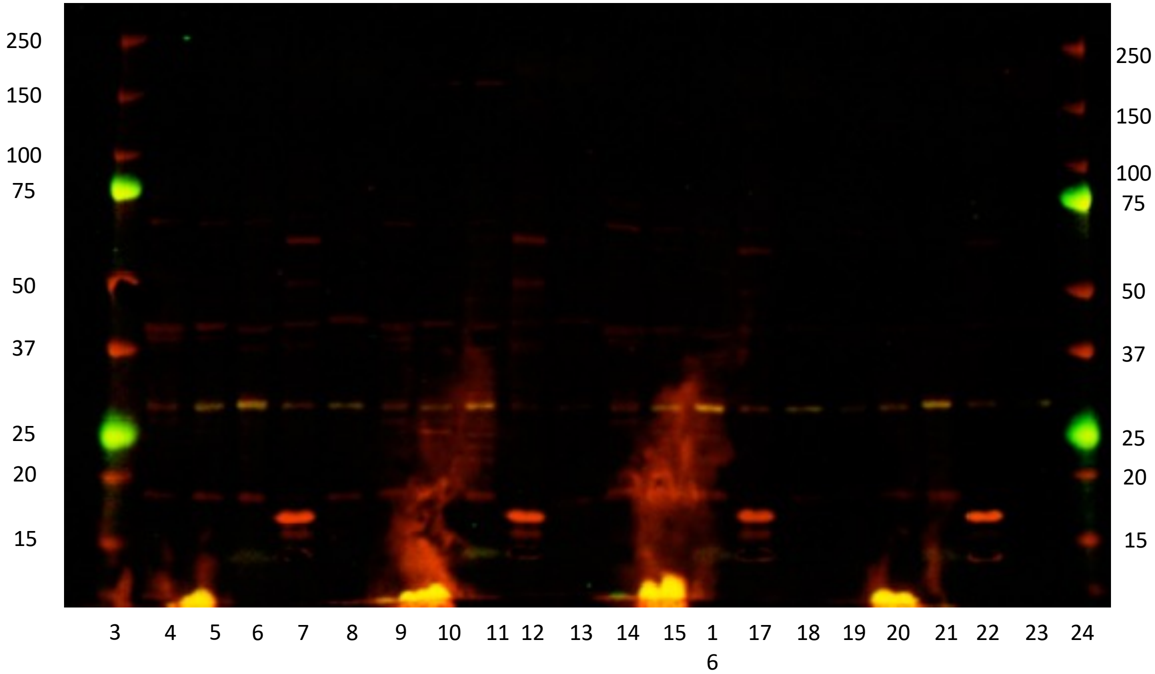

Full unedited gels for Figure 11C

Blot 2 Fibroblasts: ~~COL1A1 (Cell Signaling 72026s) 1:1000~~ (not used in figure) + MKI67 (abcam ab16667) 1:500

- Expected size of Col 1A1: ~138-220 kDa **(this was not used for the figure)**
- Expected size of Ki67: ~358 kDa
- 15uL of sample loaded per well
- Samples are organized cytoplasmic fraction, membrane bound fraction, nuclear bound fraction, chromatin bound fraction and cytoskeletal fraction as follows:
  - Wells 4 through 8 contain all fractions for Fib BF + 0.1X LIV
  - Wells 9 through 13 contain all fractions for Fib BF + 1X LIV
  - Wells 14 through 18 contain all fractions for Fib BF + 0.1X LIV + TGFb-1
  - Wells 19 through 23 contain all fractions for Fib BF + 1X LIV + TGFb-1
  - Wells 3 and 24 contain the Ladder; 2.0 µL

Full unedited gel for **Figure 11C**

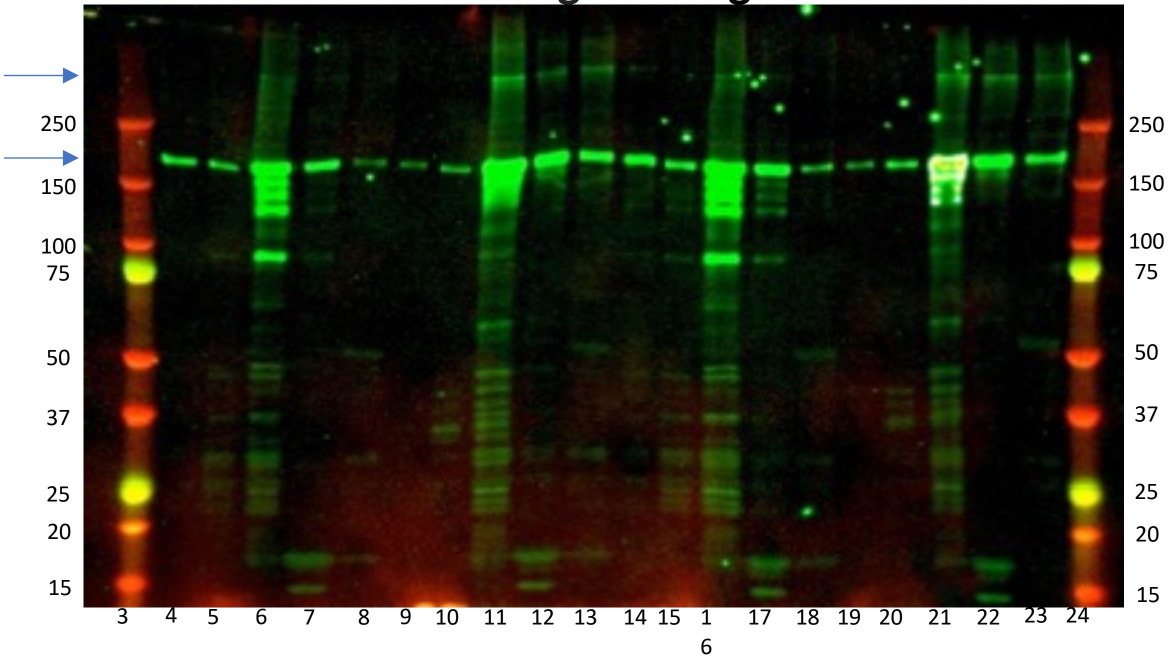

Fibroblasts: COL1A1 (Cell Signaling 72026s) 1:1000 + aSMA (Sigma A5228) 1:1000

- Expected size of Col 1A1: ~138-220 kDa
- Expected size of αSMA: ~42 kDa
- 15uL of sample loaded per well
- Samples are organized cytoplasmic fraction, membrane bound fraction, nuclear bound fraction, chromatin bound fraction and cytoskeletal fraction as follows:
    - Wells 4 through 8 contain all fractions for Fib BF + 0.1X LIV
    - Wells 9 through 13 contain all fractions for Fib BF + 1X LIV
    - Wells 14 through 18 contain all fractions for Fib BF + 0.1X LIV + TGFb-1
    - Wells 19 through 23 contain all fractions for Fib BF + 1X LIV + TGFb-1
    - Wells 3 and 24 contain the Ladder; 2.0 µL
    - **LIV = Leu, Ile, Val**

Full unedited gel for **Figure 11C**

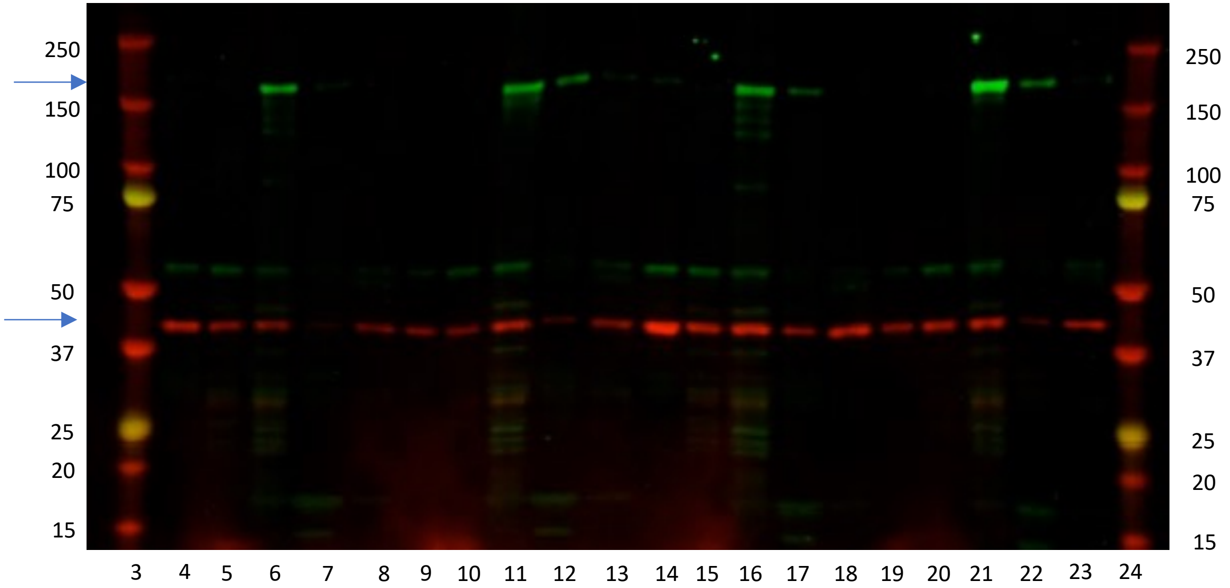

Fibroblasts: alpha Tubulin
(TUBA, abcam ab7291)
1:1000

- Expected size of alpha Tubulin: ~50 kDa
- 15uL of sample loaded per well
- Samples are organized cytoplasmic fraction, membrane bound fraction, nuclear bound fraction, chromatin bound fraction and cytoskeletal fraction as follows:
    - Wells 4 through 8 contain all fractions for Fib BF + 0.1X LIV
    - Wells 9 through 13 contain all fractions for Fib BF + 1X LIV
    - Wells 14 through 18 contain all fractions for Fib BF + 0.1X LIV + TGFb-1
    - Wells 19 through 23 contain all fractions for Fib BF + 1X LIV + TGFb-1
    - Wells 3 and 24 contain the Ladder; 2.0 µL
    - **LIV = Leu, Ile, Val**

Full unedited gel for **Figure 11C**

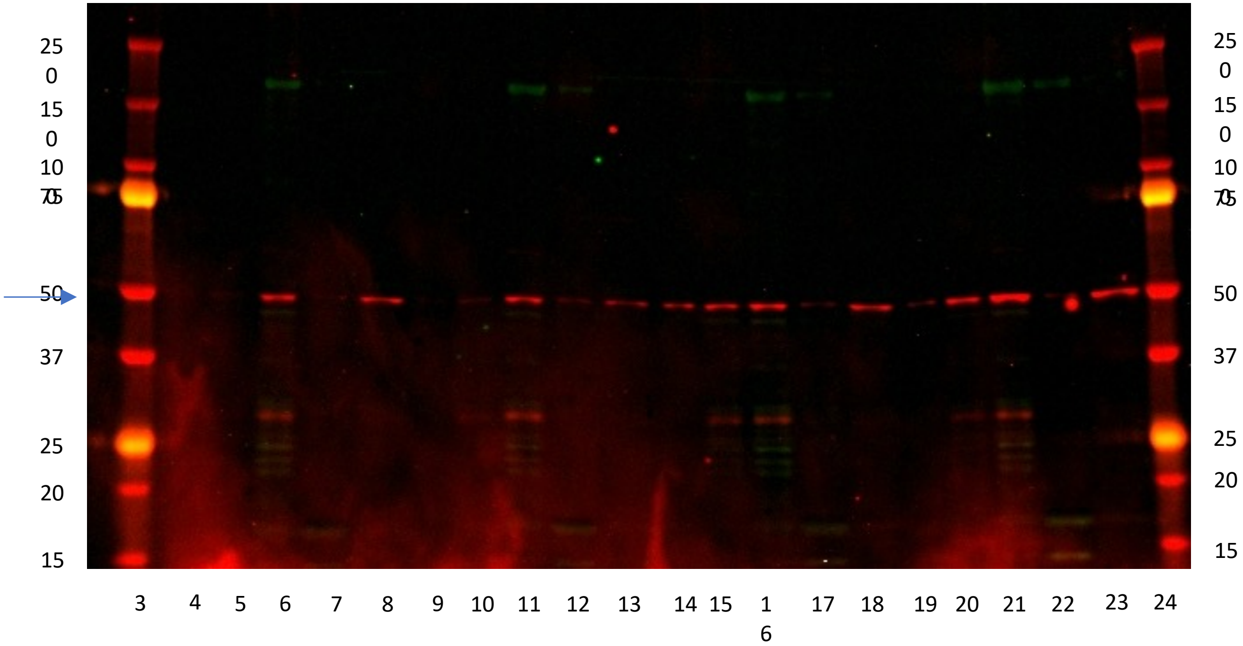

Fibroblasts: VDAC1 (Sigma SAB5700655) 1:1000 + RNA Pol II (Active Motif 102660) 1:2000

- Expected size of Vdac1: ~34 kDa
- Expected size of RNA Pol II: ~240 kDa
- 15uL of sample loaded per well
- Samples are organized cytoplasmic fraction, membrane bound fraction, nuclear bound fraction, chromatin bound fraction and cytoskeletal fraction as follows:
    - Wells 4 through 8 contain all fractions for Fib BF + 0.1X LIV
    - Wells 9 through 13 contain all fractions for Fib BF + 1X LIV
    - Wells 14 through 18 contain all fractions for Fib BF + 0.1X LIV + TGFb-1
    - Wells 19 through 23 contain all fractions for Fib BF + 1X LIV + TGFb-1
    - Wells 3 and 24 contain the Ladder; 2.0 µL
    - **LIV = Leu, Ile, Val**

Full unedited gel for **Figure 11C**

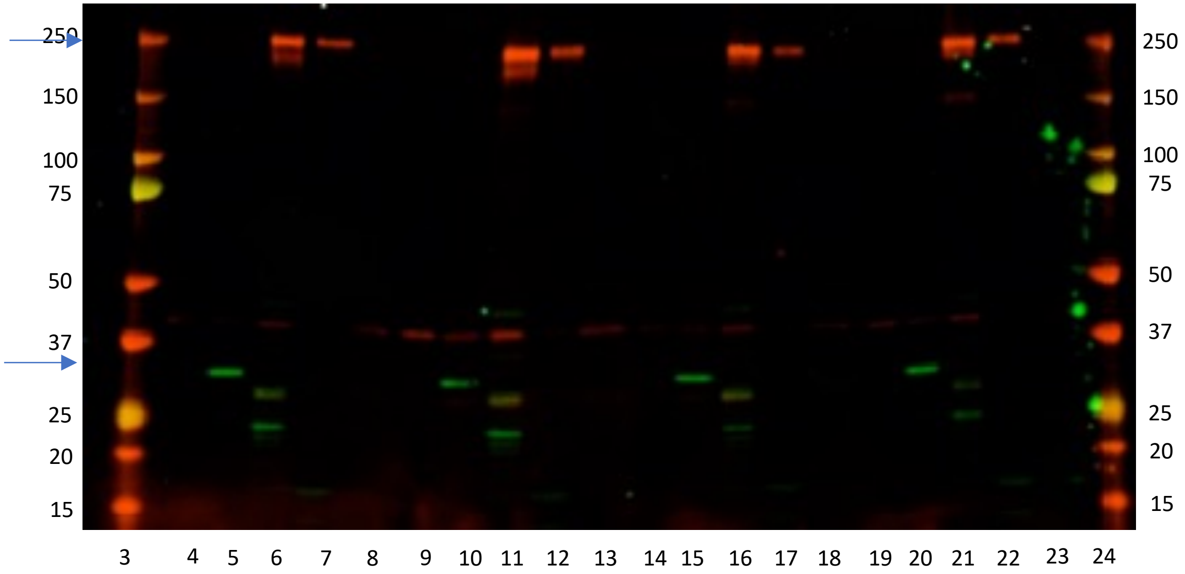

Fibroblast: H3K23Pr (Abcam, ab241466) 1:1000

- Expected size of Vdac1: ~34 kDa
- Expected size of RNA Pol II: ~240 kDa
- 15uL of sample loaded per well
- Samples are organized cytoplasmic fraction, membrane bound fraction, nuclear bound fraction, chromatin bound fraction and cytoskeletal fraction as follows:
    - Wells 4 through 8 contain all fractions for Fib BF + 0.1X LIV
    - Wells 9 through 13 contain all fractions for Fib BF + 1X LIV
    - Wells 14 through 18 contain all fractions for Fib BF + 0.1X LIV + TGFb-1
    - Wells 19 through 23 contain all fractions for Fib BF + 1X LIV + TGFb-1
    - Wells 3 and 24 contain the Ladder; 2.0 µL
    - **LIV = Leu, Ile, Val**

Full unedited gel for Figure 11C

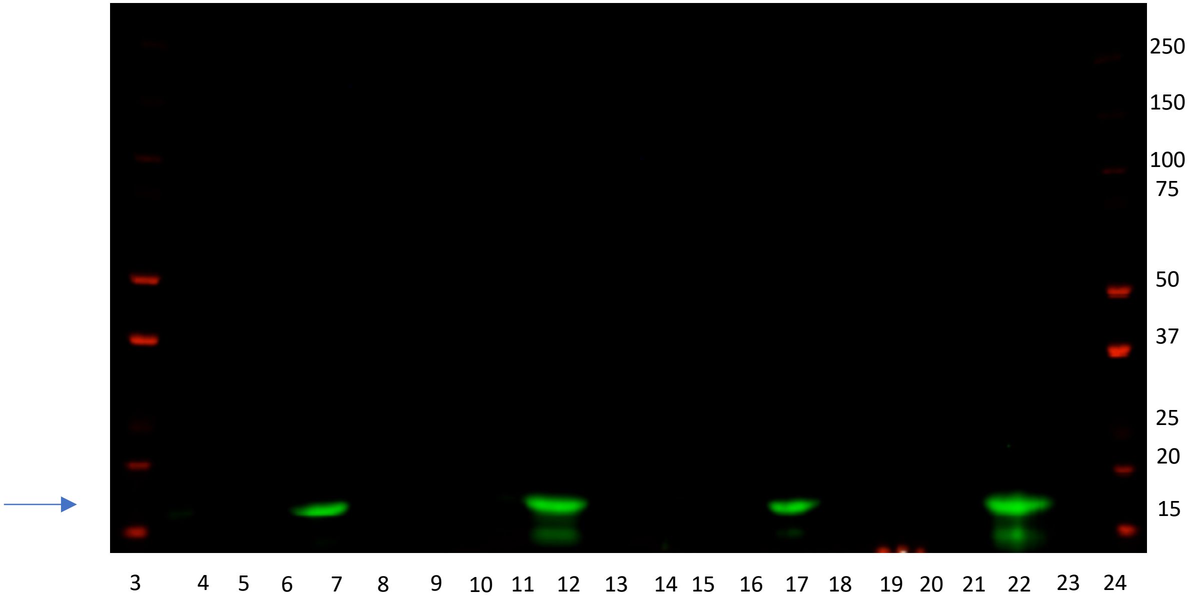

Full unedited gels for Supplementary Fig. 2S-A

BCAT2 Knockouts: BCAT2 (Sigma HPA054091) 1:1000

- Expected size: ~ 39 kDa
  - HAP-1 1
  - HAP-1 2
  - HAP-1 3
  - ΔBCAT2 1
  - ΔBCAT2 2
  - ΔBCAT2 3
- Wells 2 through 7 contain the cytoplasmic bound protein layer
- Wells 8 through 13 contain the membrane bound protein layer
- Wells 14 through 19 contain the nuclear soluble protein layer
- Wells 20 through 25 contain the chromatin bound protein layer
- Wells 1 and 26 contain the Ladder; 2.0 µL

Full unedited gel for Supplementary Fig. 2S-A

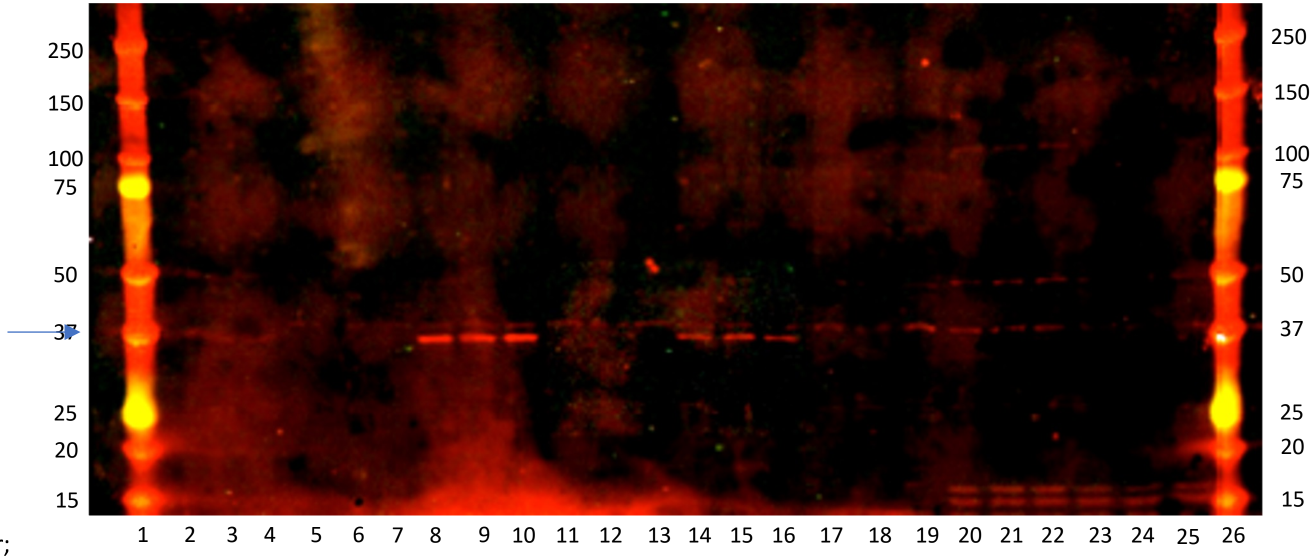

BCAT2 Knockouts: BCAT1 (Cell Signaling Technology 8875S) 1:1000

- Expected size: ~ 43 kDa
    - HAP-1 1
    - HAP-1 2
    - HAP-1 3
    - ΔBCAT2 1
    - ΔBCAT2 2
    - ΔBCAT2 3
- Wells 2 through 7 contain the cytoplasmic bound protein layer
- Wells 8 through 13 contain the membrane bound protein layer
- Wells 14 through 19 contain the nuclear soluble protein layer
- Wells 20 through 25 contain the chromatin bound protein layer
- Wells 1 and 26 contain the Ladder; 2.0 µL

Full unedited gel for Supplementary Fig. 2S-A

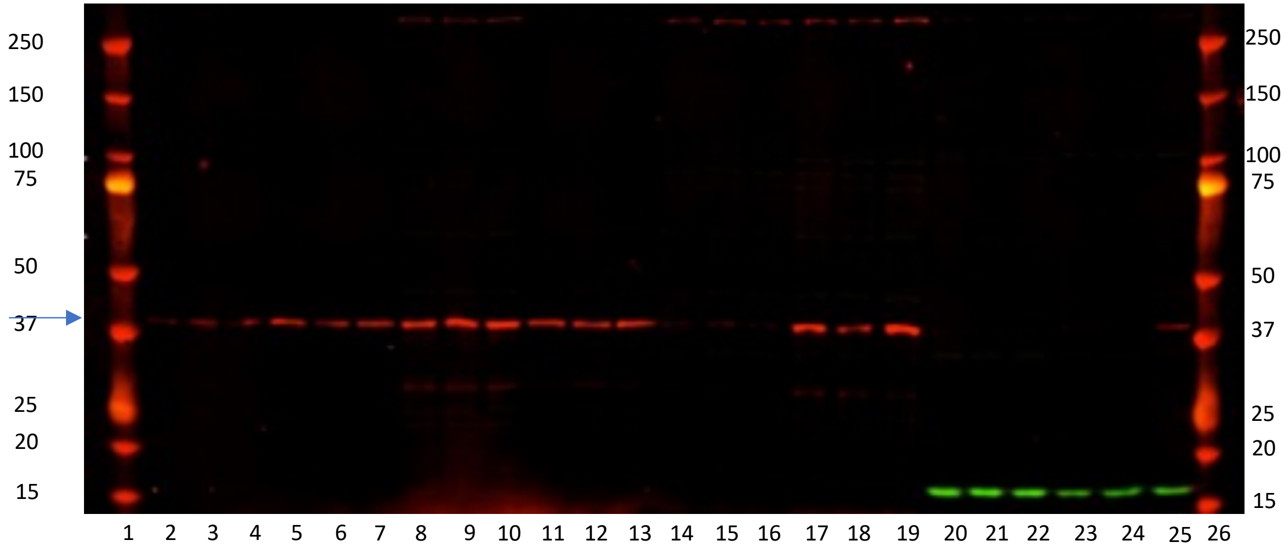

BCAT2 Knockouts: VDAC1 (Genescript A01419) 1:1000

- Expected size: ~34 kDa
  - HAP-1 1
  - HAP-1 2
  - HAP-1 3
  - ΔBCAT2 1
  - ΔBCAT2 2
  - ΔBCAT2 3
- Wells 2 through 7 contain the cytoplasmic bound protein layer
- Wells 8 through 13 contain the membrane bound protein layer
- Wells 14 through 19 contain the nuclear soluble protein layer
- Wells 20 through 25 contain the chromatin bound protein layer
- Wells 1 and 26 contain the Ladder; 2.0 µL

Full unedited gel for **Supplementary Fig. 2S-A**

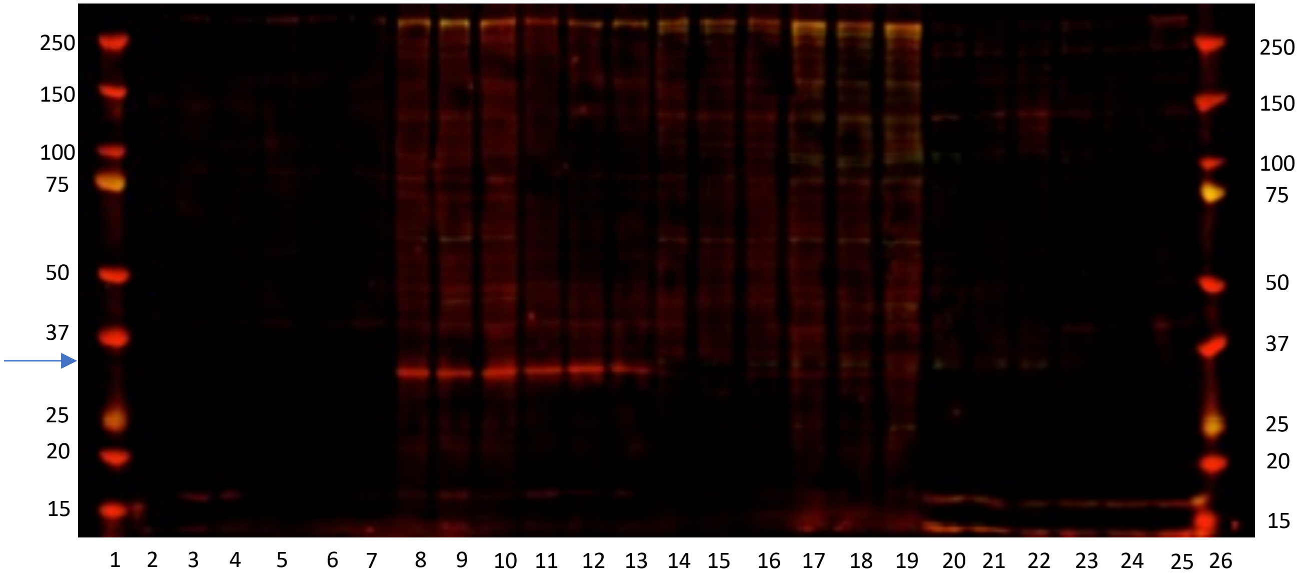

BCAT2 Knockouts: RNA Pol II (Active Motif 102660) 1:2000 ~~+ H3 (Active Motif 61475) 1:30,000~~(not used in figure)

- Expected size of RNA Pol II: ~ 240 KDa
- Expected size of H3: ~ 17 kDa
    - HAP-1 1
    - HAP-1 2
    - HAP-1 3
    - ΔBCAT2 1
    - ΔBCAT2 2
    - ΔBCAT2 3
- Wells 2 through 7 contain the cytoplasmic bound protein layer
- Wells 8 through 13 contain the membrane bound protein layer
- Wells 14 through 19 contain the nuclear soluble protein layer
- Wells 20 through 25 contain the chromatin bound protein layer
- Wells 1 and 26 contain the Ladder; 2.0 µL

Full unedited gel for **Supplementary Fig. 2S-A**

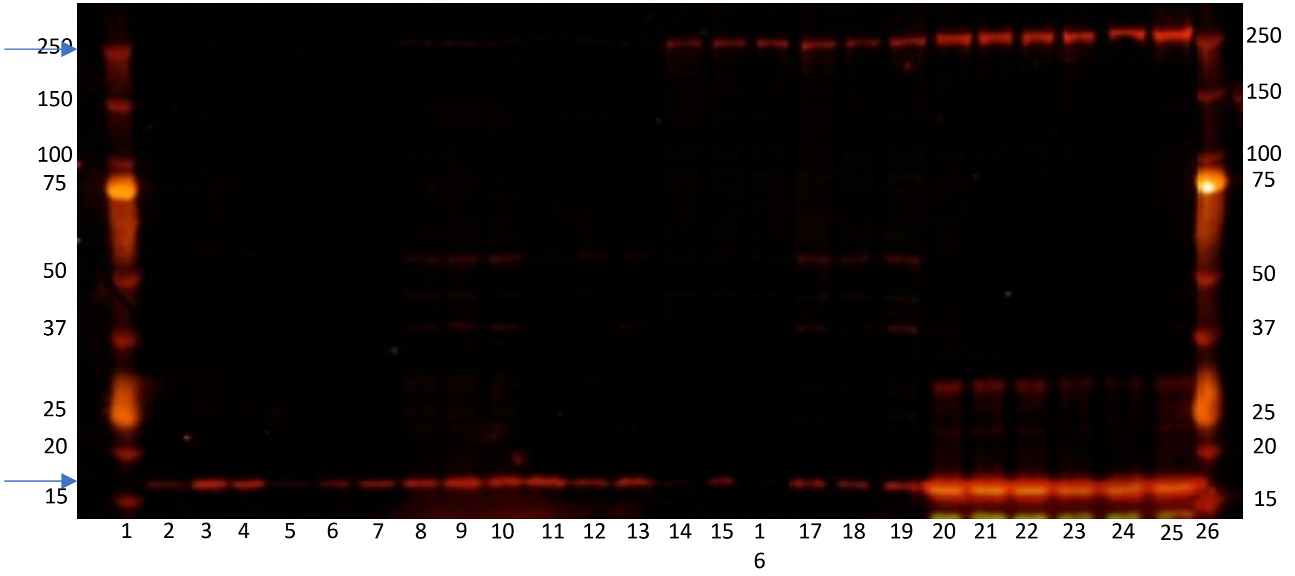

Full unedited gels for Supplementary Fig. 2S-D

ΔPCCA Cells 0.1X LIV: PCCA (abcam ab187686) 1:1000

- Expected size of PCCA: ~70 kDa
- Samples are organized as follows for all factions:
  - Hap-1 0.1X LIV 1
  - Hap-1 0.1X LIV2
  - Hap-1 0.1X LIV 3
  - ΔPCAA 0.1X LIV 1
  - ΔPCAA 0.1X LIV 2
  - ΔPCAA 0.1X LIV 3
- Wells 2 through 7 contain the cytoplasmic bound layer
- Wells 8 through 13 contain the membrane bound protein layer
- Wells 14 through 19 contain the nuclear bound protein layer
- Wells 20 through 25 contain the chromatin bound protein layer
- Wells 1 and 26 contain the Ladder; 2.0 µL

Full unedited gel for Supplementary Fig. 2S-D

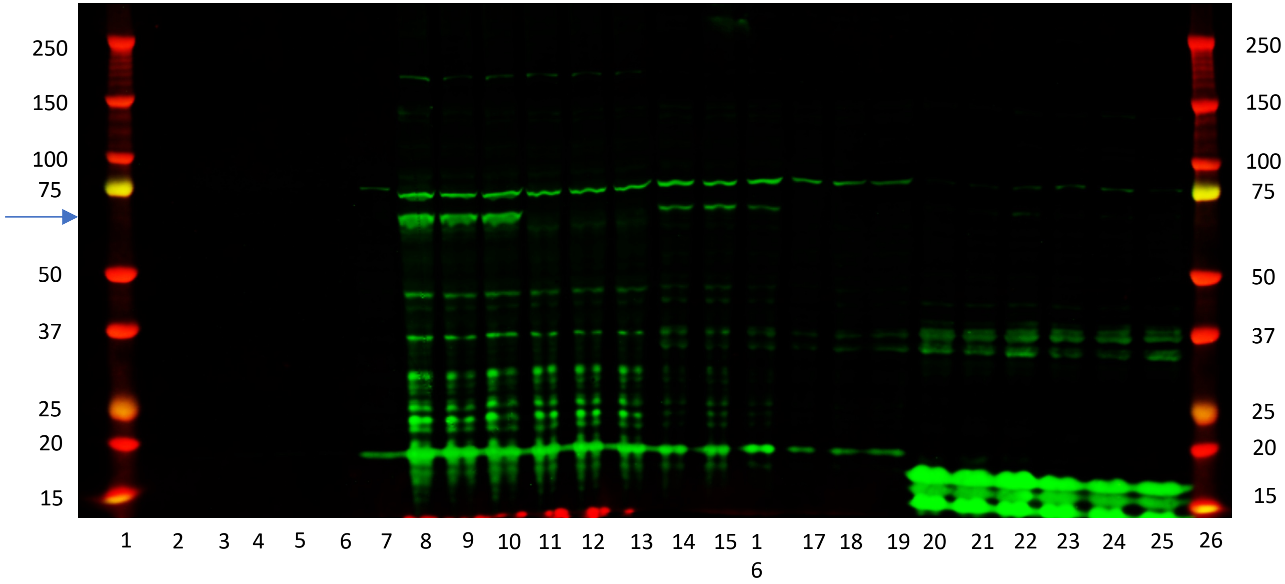

ΔPCCA Cells 0.1X LIV: Vdac1 (Genscript A01419) 1:1000 + RNA Pol II (Active Motif 102660) 1:2000 Low Exposure

- Expected size of Vdac1: ~34 kDa
- Expected size of RNA Pol II: ~240 kDa
- Samples are organized as follows for all factions:
  - Hap-1 0.1X LIV 1
  - Hap-1 0.1X LIV2
  - Hap-1 0.1X LIV 3
  - ΔPCAA 0.1X LIV 1
  - ΔPCAA 0.1X LIV 2
  - ΔPCAA 0.1X LIV 3
- Wells 2 through 7 contain the cytoplasmic bound layer
- Wells 8 through 13 contain the membrane bound protein layer
- Wells 14 through 19 contain the nuclear bound protein layer
- Wells 20 through 25 contain the chromatin bound protein layer
- Wells 1 and 26 contain the Ladder; 2.0 µL

Full unedited gel for Supplementary Fig. 2S-D

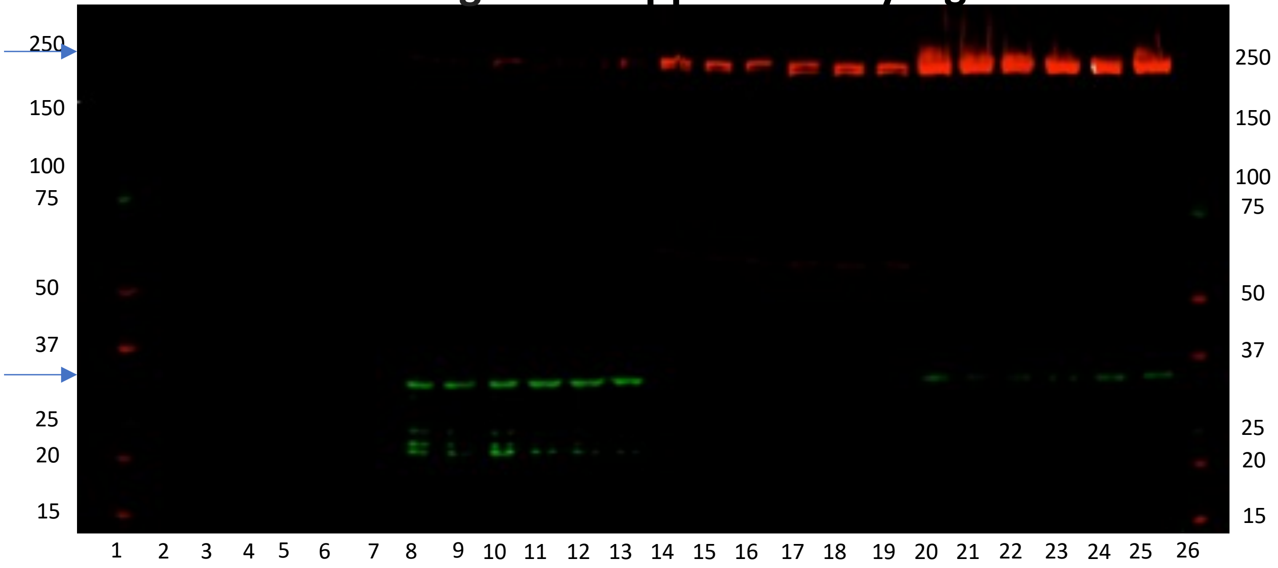

Full unedited gels for Supplementary Fig. 9S-A

Blot 3 Fibroblasts: Ki67 (abcam ab16667) 1:500

- Expected size of Ki67: ~358 kDa
- 15uL of sample loaded per well
- Samples are organized cytoplasmic fraction, membrane bound fraction, nuclear bound fraction and chromatin bound fraction as follows:
  - Wells 6 through 9 contain all fractions for Fib BF + 0.1X LIV
  - Wells 10 through 13 contain all fractions for Fib BF + 1X LIV
  - Wells 14 through 17 contain all fractions for Fib BF + 0.1X LIV + ET-1
  - Wells 18 through 21 contain all fractions for Fib BF + 1X LIV + ET-1
  - Wells 5 and 22 contain the Ladder; 2.0 µL

Full unedited gel for **Supplementary Fig. 9S-A**

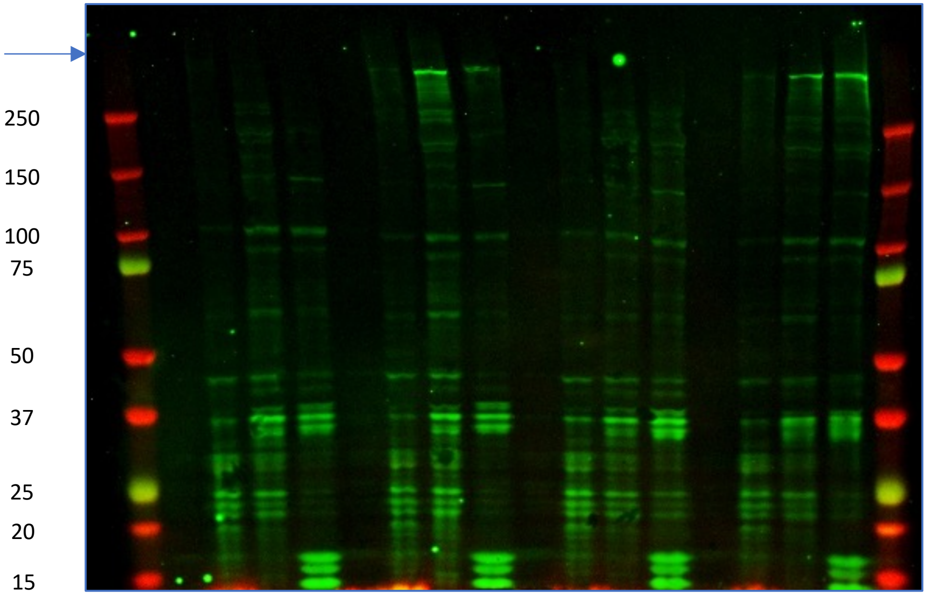

Blot 4 Fibroblasts: Col 1A1 (Cell Signaling Technology 72026s) 1:1000 Low Exposure

- Expected size of Col 1A1: ~138-220 kDa
- 15uL of sample loaded per well
- Samples are organized cytoplasmic fraction, membrane bound fraction, nuclear bound fraction and chromatin bound fraction as follows:
    - Wells 6 through 9 contain all fractions for Fib BF + 0.1X LIV
    - Wells 10 through 13 contain all fractions for Fib BF + 1X LIV
    - Wells 14 through 17 contain all fractions for Fib BF + 0.1X LIV + ET-1
    - Wells 18 through 21 contain all fractions for Fib BF + 1X LIV + ET-1
    - Wells 5 and 22 contain the Ladder; 2.0 µL
    - **LIV = Leu, Ile, Val (BCAA)**

Full unedited gel for **Supplementary Fig. 9S-A**

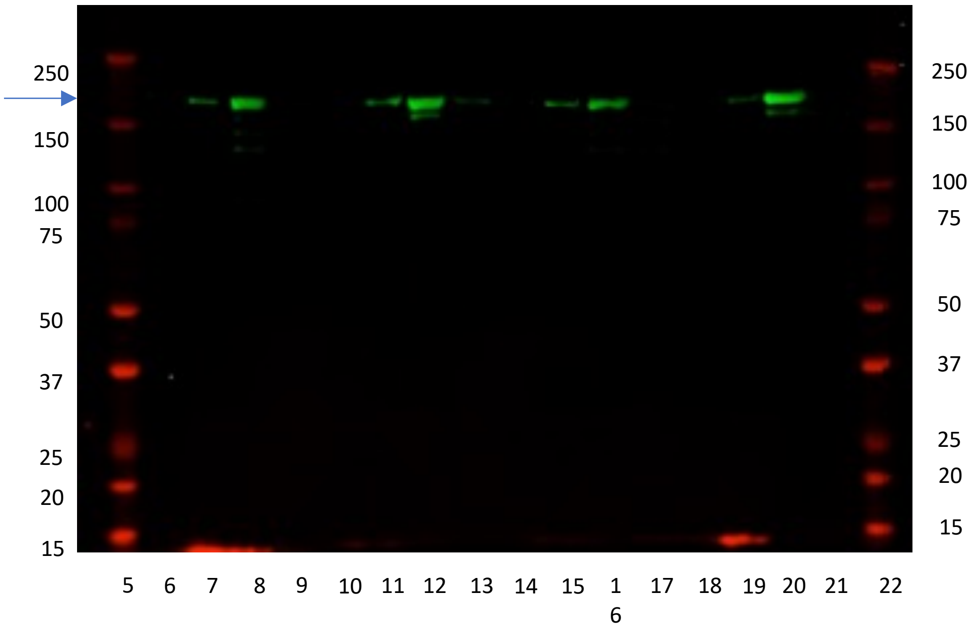

Blot 1 Fibroblasts: RNA Pol II (Active Motif 102660) 1:2000 + VAC1 (Sigma SAB5700655) 1:1000

- Expected size of RNA Pol II: ~240 kDa
- Expected size of Vdac1: ~34 kDa
- 15uL of sample loaded per well
- Samples are organized cytoplasmic fraction, membrane bound fraction, nuclear bound fraction and chromatin bound fraction as follows:
  - Wells 6 through 9 contain all fractions for Fib BF + 0.1X LIV
  - Wells 10 through 13 contain all fractions for Fib BF + 1X LIV
  - Wells 14 through 17 contain all fractions for Fib BF + 0.1X LIV + ET-1
  - Wells 18 through 21 contain all fractions for Fib BF + 1X LIV + ET-1
  - Wells 5 and 22 contain the Ladder; 2.0 µL
  - **LIV = Leu, Ile, Val (BCAA)**

Full unedited gel for Supplementary Fig. 9S-A

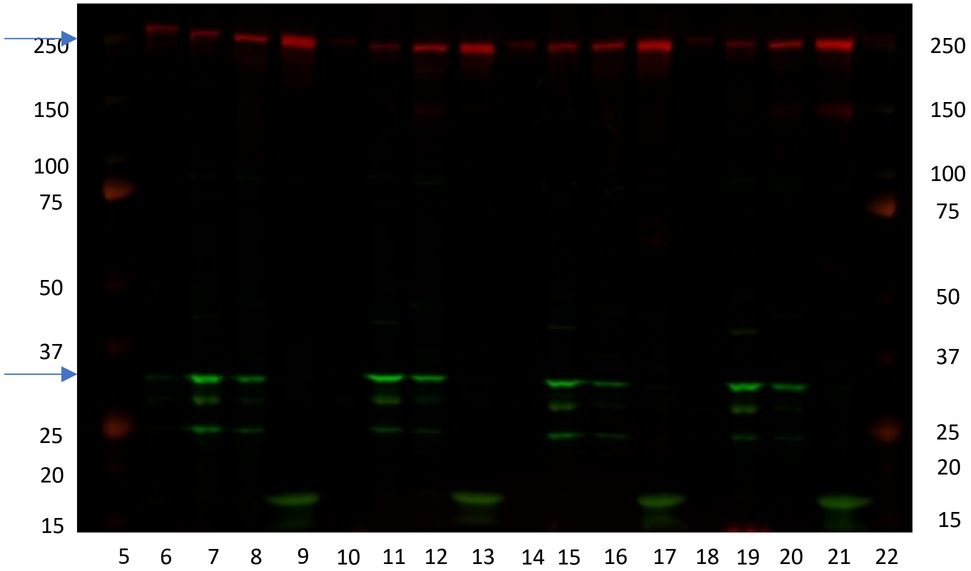

Full unedited gels for Supplementary Fig. 10S-F

Blot 1 Mouse Heart Mitochondria +/- BCAAs: BCAT2 (Sigma HPA054091) 1:1000 Low Exposure

- Expected size: ~39 kDa
- Volume of sample loaded per well determined by protein quantification using Thermo Micro BCA Protein Assay Kit; 10ug of protein
- Well 1: 1W-SHAM-BCAA-control
- Well 2: 1W-SHAM-BCAA-control
- Well 3: 1W-SHAM-BCAA-control
- Well 4: 1W-TAC-BCAA-control
- Well 5: 1W-TAC-BCAA-control
- Well 6: 1W-TAC-BCAA-control
- Well 7: 1W-SHAM-BCAA-free
- Well 8: 1W-TAC-BCAA-control
- Well 9: 1W-TAC-BCAA-control
- Well 10: 1W-TAC-BCAA-free
- Well 11: 1W-TAC-BCAA-free
- Well 12: 1W-TAC-BCAA-free
- Wells 13 contains the Ladder; 2.0 µL

Full unedited gel for **Supplementary Fig. 10S-F**

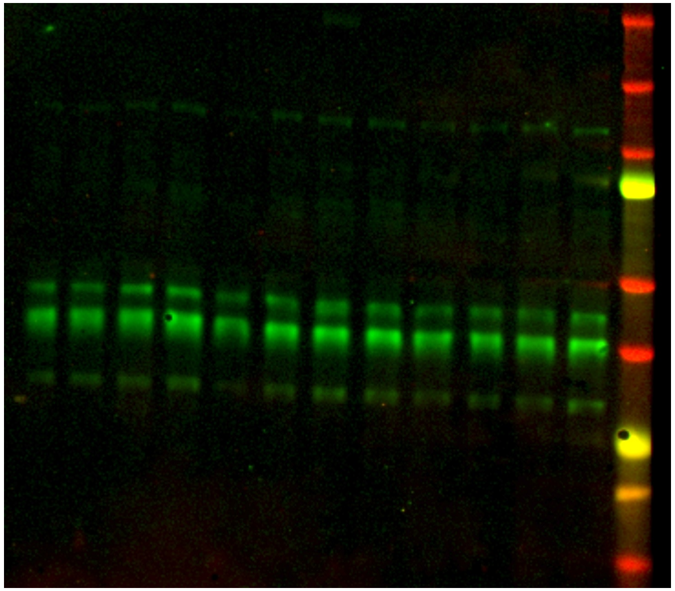

Blot 4 Mouse Heart +/- BCAAs: VDAC1 (Genescript A01419) 1:1000

- Expected size: ~34 kDa
- Volume of sample loaded per well determined by protein quantification using Thermo Micro BCA Protein Assay Kit; 10ug of protein
- Well 1: 1W-SHAM-BCAA-control
- Well 2: 1W-SHAM-BCAA-control
- Well 3: 1W-SHAM-BCAA-control
- Well 4: 1W-TAC-BCAA-control
- Well 5: 1W-TAC-BCAA-control
- Well 6: 1W-TAC-BCAA-control
- Well 7: 1W-SHAM-BCAA-free
- Well 8: 1W-TAC-BCAA-control
- Well 9: 1W-TAC-BCAA-control
- Well 10: 1W-TAC-BCAA-free
- Well 11: 1W-TAC-BCAA-free
- Well 12: 1W-TAC-BCAA-free
- Wells 13 contains the Ladder; 2.0 µL
- Wells 13 contains the Ladder; 2.0 µL

Full unedited gel for **Supplementary Fig. 10S-F**

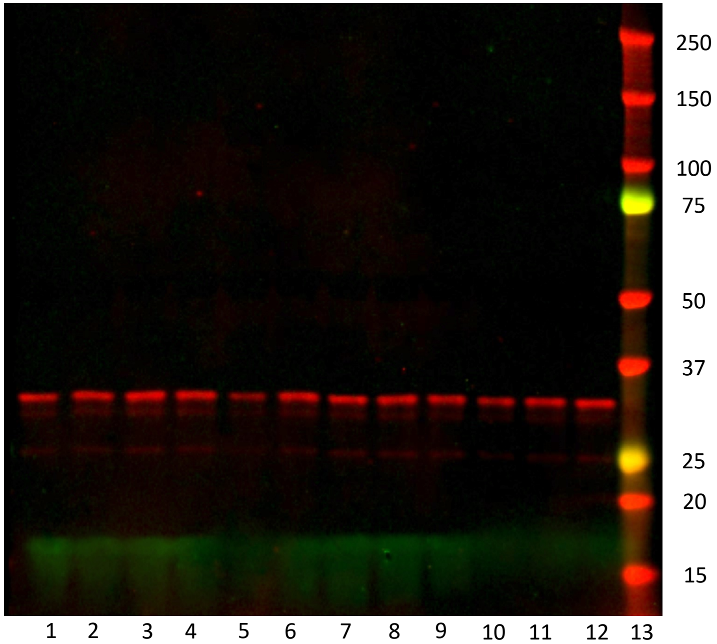

Blot 3 Mouse Heart +/-BCAAs:
NDUFS4 (Sigma HPOA003884)
1:1000 Low Exposure Reduced
Background

- Expected size: ~17 kDa
- Volume of sample loaded per well determined by protein quantification using Thermo Micro BCA Protein Assay Kit; 10ug of protein
- Well 1: 1W-SHAM-BCAA-control
- Well 2: 1W-SHAM-BCAA-control
- Well 3: 1W-SHAM-BCAA-control
- Well 4: 1W-TAC-BCAA-control
- Well 5: 1W-TAC-BCAA-control
- Well 6: 1W-TAC-BCAA-control
- Well 7: 1W-SHAM-BCAA-free
- Well 8: 1W-TAC-BCAA-control
- Well 9: 1W-TAC-BCAA-control
- Well 10: 1W-TAC-BCAA-free
- Well 11: 1W-TAC-BCAA-free
- Well 12: 1W-TAC-BCAA-free
- Wells 13 contains the Ladder; 2.0 µL
- Wells 13 contains the Ladder; 2.0 µL

Full unedited gel for Supplementary Fig. 10S-F

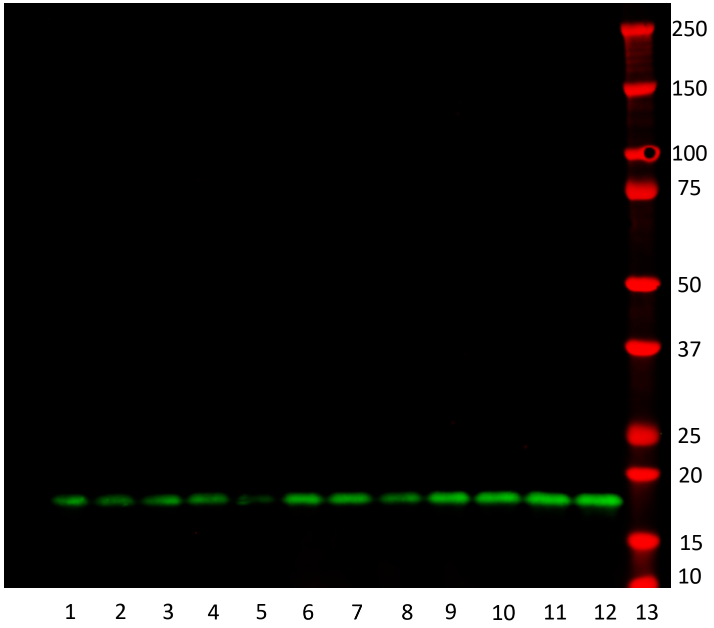

Blot 2 Mouse Heart +/- BCAAs: : BCKDHA (Sigma SAB2104058) 1:1000

- Expected size: ~50 kDa
- Volume of sample loaded per well determined by protein quantification using Thermo Micro BCA Protein Assay Kit; 10ug of protein
- Well 1: 1W-SHAM-BCAA-control
- Well 2: 1W-SHAM-BCAA-control
- Well 3: 1W-SHAM-BCAA-control
- Well 4: 1W-TAC-BCAA-control
- Well 5: 1W-TAC-BCAA-control
- Well 6: 1W-TAC-BCAA-control
- Well 7: 1W-SHAM-BCAA-free
- Well 8: 1W-TAC-BCAA-control
- Well 9: 1W-TAC-BCAA-control
- Well 10: 1W-TAC-BCAA-free
- Well 11: 1W-TAC-BCAA-free
- Well 12: 1W-TAC-BCAA-free
- Wells 13 contains the Ladder; 2.0 µL

Full unedited gel for **Supplementary Fig. 10S-F**

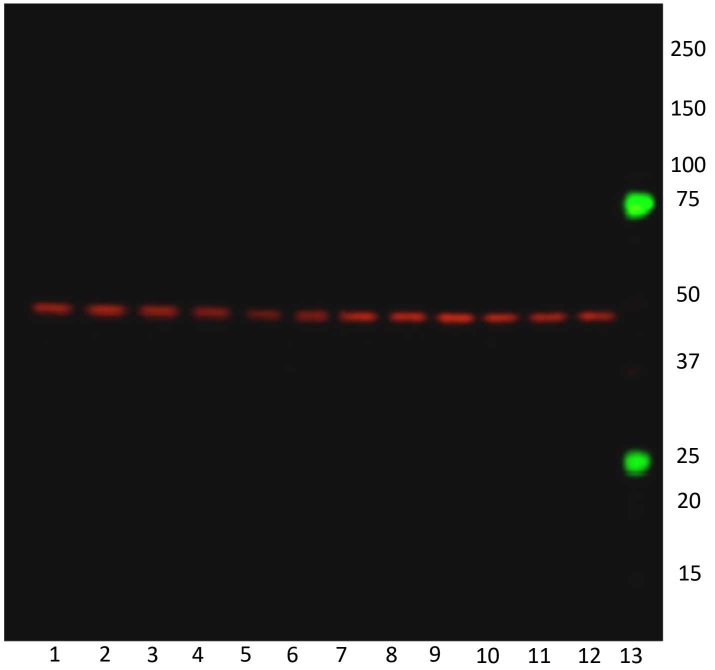

Blot 1 Mouse Heart Mitochondria +/- BCAAs: PCCA 1:1000

- Expected size: ~70 kDa
- Volume of sample loaded per well determined by protein quantification using Thermo Micro BCA Protein Assay Kit; 10ug of protein
- Well 1: 1W-SHAM-BCAA-control
- Well 2: 1W-SHAM-BCAA-control
- Well 3: 1W-SHAM-BCAA-control
- Well 4: 1W-TAC-BCAA-control
- Well 5: 1W-TAC-BCAA-control
- Well 6: 1W-TAC-BCAA-control
- Well 7: 1W-SHAM-BCAA-free
- Well 8: 1W-TAC-BCAA-control
- Well 9: 1W-TAC-BCAA-control
- Well 10: 1W-TAC-BCAA-free
- Well 11: 1W-TAC-BCAA-free
- Well 12: 1W-TAC-BCAA-free
- Wells 13 contains the Ladder; 2.0 µL

Full unedited gel for Supplementary Fig. 10S-F

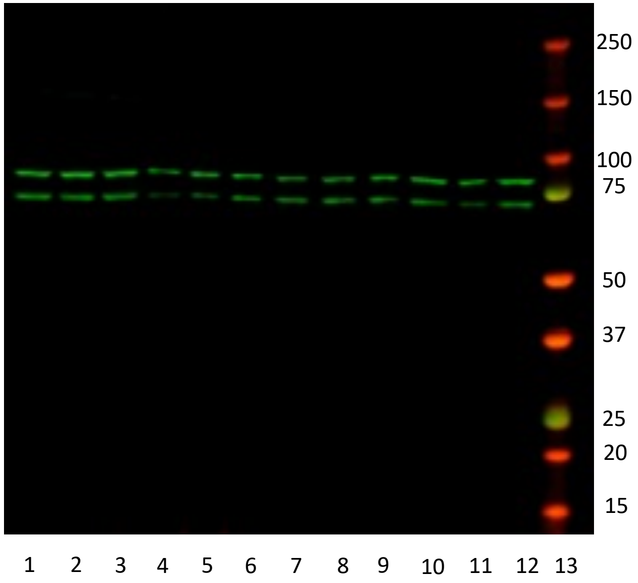

Supplement: Supplemental data [file jci-133-169399-s015.pdf]
